# An optimization method for untargeted MS-based isotopic tracing investigations of metabolism

**DOI:** 10.1007/s11306-022-01897-5

**Published:** 2022-06-16

**Authors:** Noémie Butin, Cécilia Bergès, Jean-Charles Portais, Floriant Bellvert

**Affiliations:** 1 RESTORE, CNRS ERL5311, EFS, ENVT, Inserm U1031, UPS, Université de Toulouse, Toulouse, France; 2 Toulouse Biotechnology Institute, TBI-INSA de Toulouse INSA/ CNRS 5504-UMR INSA/INRA 792, 5504 Toulouse, France; 3 MetaboHUB-MetaToul, National Infrastructure of Metabolomics and Fluxomics, 31077 Toulouse, France

**Keywords:** Isotope labelling experiments, Untargeted analysis, Parameter optimization, LC/MS

## Abstract

**Introduction:**

Stable isotope tracer studies are increasingly applied to explore metabolism from the detailed analysis of tracer incorporation into metabolites. Untargeted LC/MS approaches have recently emerged and provide potent methods for expanding the dimension and complexity of the metabolic networks that can be investigated. A number of software tools have been developed to process the highly complex MS data collected in such studies; however, a method to optimize the extraction of valuable isotopic data is lacking.

**Objectives:**

To develop and validate a method to optimize automated data processing for untargeted MS-based isotopic tracing investigations of metabolism.

**Methods:**

The method is based on the application of a suitable reference material to rationally perform parameter optimization throughout the complete data processing workflow. It was applied in the context of ^13^C-labelling experiments and with two different software, namely geoRge and X13CMS. It was illustrated with the study of a *E. coli* mutant impaired for central metabolism.

**Results:**

The optimization methodology provided significant gain in the number and quality of extracted isotopic data, independently of the software considered. Pascal triangle samples are well suited for such purpose since they allow both the identification of analytical issues and optimization of data processing at the same time.

**Conclusion:**

The proposed method maximizes the biological value of untargeted MS-based isotopic tracing investigations by revealing the full metabolic information that is encoded in the labelling patterns of metabolites.

**Supplementary Information:**

The online version contains supplementary material available at 10.1007/s11306-022-01897-5.

## Introduction

Stable-isotope labelling experiments coupled with mass spectrometry (MS) are increasingly used to obtain a comprehensive understanding of metabolism in many fields of biology, biotechnology, and medicine (Wittman, 2002, Chokkathukalam et al., 2014; Zaimenko et al., 2017). In such investigations, an isotope tracer (most commonly ^13^C in metabolic studies) is fed to a biological system of interest (cells, tissues, whole organisms). The incorporation of the labelled atom into metabolites is measured by MS and provides valuable information on metabolic pathways (pathway profiling) and metabolic fluxes (fluxomics) (Wittman, 2002; Wiechert et al., 2001; Wiechert, 2001; Zamboni et al., 2009). These approaches were initially developed by exploiting targeted MS methods in which the labelling patterns of selected metabolites—hence of selected metabolic pathways—could be measured (Chokkathukalam et al., 2014; Stuani et al.,; 2018). Progress in MS instrumentation and methods has led to the recent emergence of untargeted approaches with the potential to access the labelling patterns of a much larger number of metabolites, resulting in significant gains in the coverage of cellular and tissular metabolic processes (Creek et al., 2012; Zamboni et al., 2015). Similar to untargeted metabolomics, which aims at maximizing the number of detected metabolites, untargeted isotopic profiling aims at maximizing the number of isotopic data—i.e. the number of measured isotopologue abundances—collected from isotopically labelled material using appropriate analytical methods and data processing tools (Hiller et al., 2010; Chokkathukalam et al. 2012; Bueschl et al., 2014, Kluger et al., 2014; Capellades et al., 2016, Weindl et al., 2016). Data processing in untargeted isotopic tracing studies is a real challenge, firstly because the MS spectra collected on labelled material are much more complex than those of (the same) unlabelled material. Potentially all the isotopologues of each metabolite in the labelled samples can be generated and detected. Given the high molecular complexity of typical biological samples, the total number of peaks in the MS spectra is drastically increased. Moreover, since the total intensity of the MS signal from a given analyte is the same whether a compound is labelled or not, the MS spectra of labelled compounds contain more signals each with lower intensities than in the corresponding unlabelled spectra. The MS spectra of labelled material therefore contain more peaks with lower intensities than those of equivalent unlabelled samples.

The untargeted processing of MS data from labelled material is also more complex. The extraction of isotopologues from the raw MS data is basically the same process as in unlabelled metabolomics so that the same tools—such as XCMS (Kessner et al., 2008), MS-Dial (Tsugawa et al., 2015), MZmine 2 (Pluskal et al., 2010)—can be used in both cases. However, the task of regrouping isotopologues into isotopic clusters is specific to isotopic studies. A number of dedicated software tools have been developed, such as X13CMS (Patti et al., 2014), geoRge (Capellades et al., 2016), MetExtractII (Buelschl et al., 2017), mzMatchIso (Chokkathukalam et al., 2012), DynaMet (Kiefer et al., 2015) and HiResTec (Hoffmann et al., 2018). Considering the wealth of information to be exploited in untargeted isotopic studies, the processing software needs to be robust and efficient in maximizing the number and quality of the extracted data. Comparisons of these programs (Capellades et al., 2016; Dange et al., 2020) have highlighted the differences in requirements, algorithms, and parameter optimizations between the different tools, as well as inconsistencies (non-detection of known peaks, inconsistent isotopic clusters, abnormal redundancy, etc.) in the results obtained. This can be explained in part by the newness of these programs, which will likely be improved in the near future. It can also be explained by the challenge that parameter optimization represents in such a complex, multi-step data processing workflow. Indeed, no rational strategy to optimize the recovery of all the available information in raw MS data has yet been proposed.

In this article, we present a method for optimizing MS-based untargeted isotopic tracing experiments by maximizing the amount and quality of the isotopic information that can be extracted from the analytical data. This method is based on the use of a suitable reference material to rationally perform parameter optimization throughout the processing workflow. It is applied here for ^13^C-labelling experiments analysed with geoRge and X13CMS, but the approach is generic and can be used with any similar program or labelling strategy. We demonstrate it here for the study of a well-described *E.coli* mutant with altered metabolic fluxes.

## Experimental section

### Preparation of biological samples

#### Reference material: the Pascal triangle sample

The ‘Pascal Triangle’ (PT) sample was produced biologically as described by Millard et al. (2014). Briefly, *Escherichia coli* K-12 MG1655 was grown in minimal medium with a mixture of unlabelled + ^13^C-labelled acetate as sole carbon source. This mixture consisted of the four different (carbon) isotopic forms of acetate in equal proportions, i.e., 25% of U-^12^C-acetate, 25% of 1-^13^C-acetate, 25% of 2-^13^C-acetate, and 25% of U-^13^C-acetate. The actual isotopic composition of this mixture was controlled by quantitative ^1^H NMR before use. A similar culture was performed with only unlabelled acetate to produce the unlabelled PT sample. Cells were grown in a 500 mL Multifors Bioreactor (Infors HT, Bottmingen-Basel, Switzerland) under pH control (pH 7.0). Cell growth was monitored by measuring the optical density at 600 nm with a Genesys 6 spectrophotometer (Thermo, Carlsbad, CA, USA). Intracellular metabolites were sampled by fast filtration (Kiefer et al., 2007; Millard et al., 2014) from cells collected in the mid-exponential growth phase. Samples (2 mL) of cell culture were rapidly dropped on a filter (Sartolon Polyamide 0.2 μm) to eliminate the culture medium. The filter was rinsed with 2 mL of washing solution (NaCl 0.9% with 5 mM of acetate), quickly removed from the filtration unit, then placed in a precooled centrifuge tube containing 5 mL of ACN/MeOH/H2Omq (2/2/1) with 125 mM formic acid for metabolite extraction and incubated for 20 min at − 20 °C. The tubes were then centrifuged for 5 min at 2000×*g* and the supernatant was evaporated (Savant SC250 EXP 230 Speedvac, ThermoFisher) and resuspended in 100 µL of water before LC–MS analysis.

#### *E. coli* samples

Two *E.coli* BW-25113 strains from the Keio collection (Baba et al., 2006) were used: BW25113 wild type, and BW25113 ∆*zwf*.

Both strains were first cultured in LB medium (10 g/L tryptone, 5 g/L yeast extract and 10 g/L NaCl) with kanamycine (25 μg/ml) at 37 °C overnight and then stored in glycerol stock. The strains were then inoculated from a glycerol stock and first cultured in 48-well microplates in liquid LB medium. The LB cultures were used to inoculate preculture cells in 48-well microplates in minimal synthetic medium containing 17.4 g/L Na_2_HPO_4_,12H_2_0, 3.03 g/L of KH_2_PO_4_, 0.51 g/L NaCl, 2.04 g/L NH_4_Cl, 0.49 g/L MgSO_4_, 4.38 mg/L CaCl_2_, 15 mg/L Na_2_EDTA 2H_2_O, 4.5 mg/L ZnSO_4_ 7H_2_O, 0.3 mg/L CoCl_2_ 17.6H_2_O, 1 mg/L MnCl_2_ 4H_2_O, 1 mg/L of H_3_BO_3_, 0.4 mg/L Na_2_MoO_4_ 2H_2_O, 3 mg/L FeSO_4_ 7H_2_O, 0.3 mg/L CuSO_4_ 5H_2_O, 0.1 g/L thiamine and 3 g/L glucose. The M9 precultures were used to inoculate cells grown in minimal medium containing 3.48 g/L Na_2_HPO_4_,12H_2_0, 0.606 g/L KH_2_PO_4_, 0.51 g/L NaCl, 2.04 g/L NH_4_Cl, 0.098 g/L MgSO_4_, 4.38 mg/L CaCl_2_, 15 mg/L Na2EDTA 2H_2_O, 4.5 mg/L ZnSO_4_ 7H_2_O, 0.3 mg/L CoCl_2_ 17. 6H_2_O, 1 mg/L MnCl_2_ 4H_2_O, 1 mg/L H_3_BO_3_, 0.4 mg/L Na_2_MoO_4_ 2H_2_O, 3 mg/L FeSO_4_ 7H_2_O, 0.3 mg/L CuSO_4_ 5H_2_O, 0.1 g/L thiamine and 3 g/L glucose. These cultures were performed in 48 15 ml bioreactors under controlled growth conditions using a robotic platform (Freedom EVO 200, Tecan), with collection of labelled samples (biomass or cultivation medium) at defined culture times or optical densities. This cell culture robot and its operation are described in detail in Heux et al. (2014) and Bergès et al. (2021).

The cultures were carried out with either unlabelled glucose or a mixture of 80% [1-^13^C1]-d-glucose + 20% [U-^13^C6]-d-glucose. To minimize sources of unlabelled carbon atoms from the first culture steps in the latter experiments, cells were inoculated at a starting OD of between 0.04 and 0.076 from pre-cultures grown with the same medium and the same (unlabelled or labelled) carbon sources as the cultures.

All cultures were performed in 15 mL reaction vessels, at 37 °C, pH 7, a stirring speed of 2300 rpm and with 5 L/min of compressed air flowing through the culture module. Intracellular metabolites were automatically sampled in each bioreactor when OD_600nm_ = 1.2 was reached. Samples (200 µL) were extracted and quenched in 2 mL of acetonitrile/methanol/water (4/4/2) with 125 mM formic acid at − 20 °C. These 2 mL were then evaporated in a SpeedVac and resuspend in 200 µL of water before LC-HRMS analysis. All samples were produced in five replicates.

### LC/MS measurements

LC/MS analyses were performed using an ICS5000 + ion chromatography system (Dionex, CA, US) coupled to an Orbitrap Q Exactive + mass spectrometer (Thermo Fisher Scientific, Waltham, MA, USA) operated in negative electrospray ionization (ESI−) mode. Central metabolites were separated on an ionic chromatography column IonPac AS11 (250 × 2 mm i.d.; Dionex, CA, USA). The mobile phase was a KOH gradient at a flow rate of 380 μL/min, varied as follows: 0 min, 0.5 mM; 1 min, 0.5 mM; 9.5 min, 4.1 mM; 14.6 min, 4.1 mM; 24 min, 9.65 mM; 31.1 min, 90 mM; and 43 min, 90 mM. The column was then equilibrated for 5 min at the initial conditions before the next sample was analysed. The injection volume was 15 μL.

MS analyses were performed in full-scan mode at a resolution of 70 000 (at 400 m/z) over the m/z range 80–1000. Data were acquired with the following source parameters: the capillary temperature was 350 °C, the source heater temperature, 350 °C, the sheath gas flow rate, 50 a.u. (arbitrary units), the auxiliary gas flow rate, 10 a.u., the S-Lens RF level, 65%, and the source voltage, 2.75 kV.

The data were acquired in a single analytical batch. As in untargeted metabolomics approaches, all the biological samples were randomized in the analytical run and the five-replicates of the reference sample were injected at regular intervals throughout the experiment. Raw LC/MS data were converted into the open “mzXML” format using the software Proteowizard (Kessner et al., 2008). The raw data were cut after 42 min to retain all essential information while avoiding artefacts from the cleaning step and reducing data size. Figure S-1 shows the Graphical User Interface of MSConvert.

### Data processing

#### Reference data

Twenty-five metabolites covering representative metabolite classes were selected as reference metabolites: organic acids (fumarate, succinate, malate, orotate, alpha-ketoglutarate (α-KG), citrate), phosphorylated compounds (2 and 3-phosphoglycerate (2/3-PG), phosphoenolpyruvate (PEP), glycerol-3phosphate (Gly-3P), 5-phosphoribosyl-pyrophosphate (PRPP), pentose-5-phosphate (P5P), fructose-1,6-diphosphate (FBP), sedoheptulose-7-phosphate (Sed7P), glucose-1-phosphate (G1P), glucose-6-phosphate (G6P), fructose-6-phosphate (F6P), mannose-6-phosphate (Man6P)), and nucleotides (adenosine diphosphate (ADP), adenosine triphosphate (ATP), cytidine diphosphate (CDP), cytidine triphosphate (CTP), guanosine diphosphate (GDP), uridine diphosphate (UDP), uridine monophosphate (UMP), uridine triphosphate (UTP)). All these compounds were identified in the MS data with a confidence level 1 (Creek et al., 2014), including confirmation with authentic compounds.

The isotopologues from these metabolites were extracted from the MS data collected on the reference material, and were assigned to molecular isotopic clusters in a targeted manner with the software Emzed (Kiefer et al., 2013) using a mass tolerance of 0.003 m/z. Carbon isotopologue distributions (CIDs) of the reference metabolites were then quantified from the corresponding mass fractions after correcting for the presence of all naturally occurring isotopes and the isotopic purity of the tracer (99%) using the software IsoCor, v2.0.4 (Millard et al., 2019). The complete dataset (including the list of reference metabolites, the isotopologues, their analytical characterics, their abundances, the isotopic clusters and the metabolite CIDs) is detailed in Supplementary Information Table S1 and was used as reference material to evaluate the optimization of data extraction.

#### Detection of LC/MS features using XCMS

LC/MS features were extracted using the XCMS package (Smith et al., 2006) in Rstudio. The isotopologue parameters optimization (IPO) tool (Libiseller et al., 2015) was first used to optimize XCMS parameters, using unlabelled samples (*E.coli*) as required. The set of parameters selected using the IPO tool are given in SI Table S2, and were used as starting settings for subsequent data processing optimization.

All raw datasets (i.e. from unlabelled and labelled PT samples and *E.coli* samples) were grouped and processed in a single batch with XCMS (Smith et al., 2006) so that peaks were identified and integrated using exactly the same processing parameters. This operation was iteratively repeated after changing the parameter settings to minimize the difference between the XCMS data and a reference dataset, as explained in the Results section. The XCMS parameters and their tested range of values are described in SI Table S3. The parameters giving the optimal recovery of the reference data are given in SI Table S2.

#### Isotopologue clustering

The XCMS object containing the list of putative isotopologues was processed separately with the R packages X13CMS (Patti et al. 2014) and geoRge (Cappellades et al. 2016). The parameters for the two programs are listed in SI Table S4.

#### Calculation of CIDs

Carbon isotopologue distributions were calculated from the relevant mass fractions of isotopic clusters after correcting for naturally occurring isotopes of elements other than carbon using IsoCor (Millard et al., 2019), accounting also for the MS resolution. The CIDs of metabolites in the PT samples can be predicted from the composition of the label input and the number of carbon atoms in the metabolites. The theoretical CIDs of metabolites in the PT sample were calculated using the equation

$${M}_{k}=\left( \begin{array}{c}n\\ k\end{array}\right)*{p}^{k}*{\left(1-p\right)}^{n-k}$$

where *n* is the total number of carbon atoms in a molecular entity with *k* ^13^C atoms and *p* is the abundance of ^13^C isotopes. Here, the molecular enrichment of ^13^C-acetate measured by NMR was *p* = 0.512. Standard deviations of measured CIDs were determined from the analysis of five analytical replicates of the PT sample.

### Statistical analyses

Principal Component Analysis (PCA) was applied to all the biological samples (unlabelled and labelled *E.coli* strains). PCA was performed using SIMCA [REF, v 15.0.02.5959] to separate all the biological samples (unlabelled and labelled *E.coli* strains) into different classes. A Wilcoxon test (p value ≤ 0.05) was used to identify the most discriminating isotopologues between the two *E.coli* strains. An in-house database with 47 metabolites was then used for metabolite identification based on exact masses and standard retention times (RTs). Metabolite identification was confirmed with authentic compounds.

### Evaluation criteria for processing optimization

This study is primarily based on the establishment of specific metrics to evaluate isotopic measurements and validate software parameters used to process data in untargeted MS-based isotopic tracing investigations of metabolism. We used the criteria established by Heuillet et al. (2018) to validate MS-based isotopic measurements:

- The mass accuracy, i.e., the error on isotopologue masses, estimated from the difference between the theoretical (M_th_) and experimental (M_exp_) mass of each isotopologue.
  $${\text{mass accuracy }}\left( {{\text{ppm}}} \right) \, = \, \left( {{\text{M}}_{{{\text{th}}}} {-}{\text{M}}_{{{\text{exp}}}} } \right)/{\text{M}}_{{{\text{th}}}} \times {1}0^{{6}}$$
- The RT accuracy*,* i.e., the error on the measured RTs, calculated from the difference between theoretical and measured RTs.
  $${\text{RT accuracy }}\left( {\text{s}} \right) = \left( {{\text{RT}}_{{{\text{th}}}} {-}{\text{RT}}_{{{\text{exp}}}} } \right)/{\text{RT}}_{{{\text{th}}}} \times {1}0000$$
- The RT isotopic deviation, i.e., the measured deviation of RTs between isotopologues belonging to the same isotopic cluster.
- The area precision, i.e., the spread of measured areas, estimated from the standard deviation of measurements on PT sample replicates.
- The CID accuracy (CID mean bias), i.e., the error on measured CIDs, simply the difference between predicted and measured CIDs.
  $${\text{CID accuracy}} = {\text{CID}}_{{{\text{th}}}} - {\text{ CID}}_{{{\text{exp}}}}$$

We also used two further criteria to evaluate the closeness of the clustering data obtained with the two ^13^C-clustering programs to the clusters obtained by manual analysis:

- The recall, i.e. the ability of the process to retrieve the information, calculated as follows:
  $$\mathrm{recall}= \frac{\left\{\text{relevant isotopic \,clusters}\right\}\cap \{\text{retrieved isotopic clusters}\}}{\{\text{relevant isotopic clusters}\}}$$
- The cluster precision, i.e., a measure of the relevance of the retrieved information, defined by:
  $$\mathrm{cluster\, precision}= \frac{\left\{\text{relevant isotopic clusters}\right\}\cap \{\text{retrieved isotopic clusters}\}}{\{\text{retrieved isotopic clusters}\}}$$

## Results and discussion

### Overall strategy and case study

**Fig. 1:**
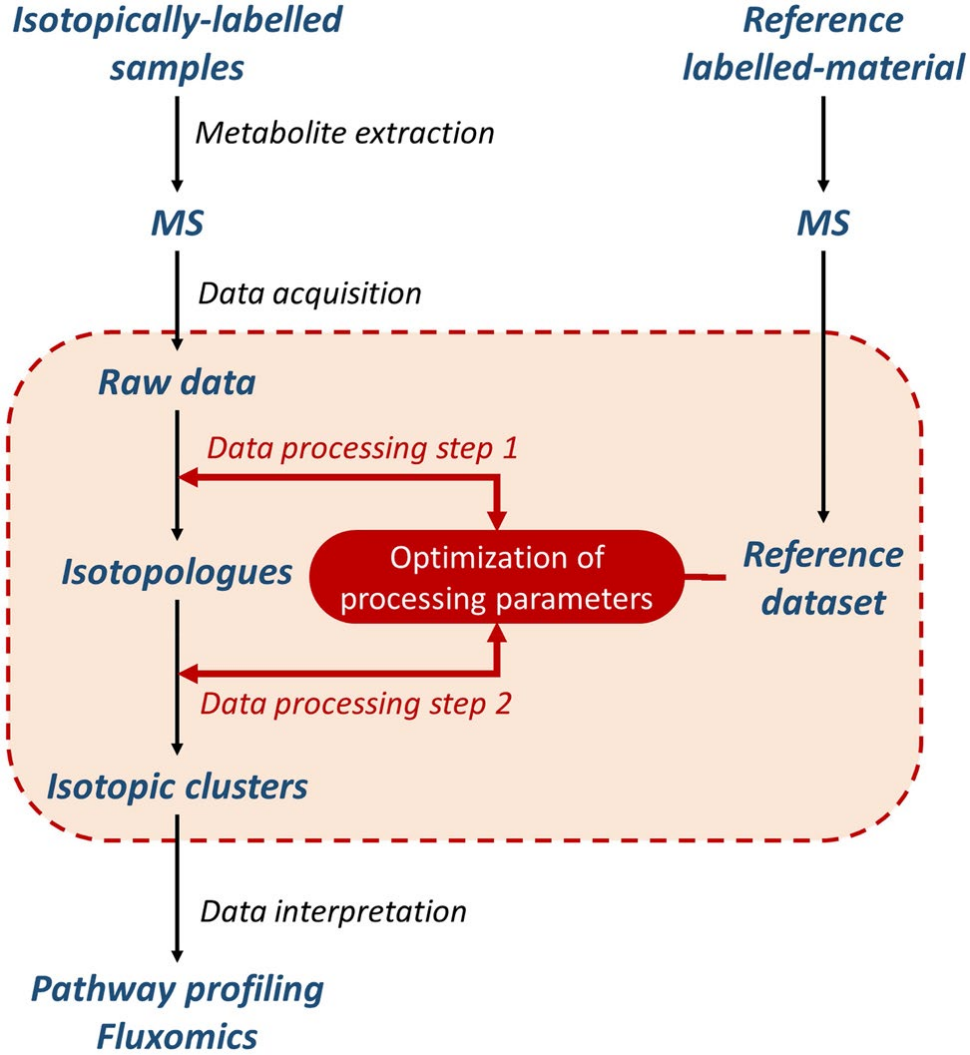
Strategy for software parameter optimization in untargeted MS-based isotopic profiling using a reference labelled material

The aim of this work was to optimize data processing in untargeted MS-based isotopic tracing studies of metabolism, which refers here to isotope-labelling experiments aiming at the identification of metabolic pathways from the detailed examination of the label incorporation into metabolites. In contrast to isotope-assisted metabolomics in which a labelled sample with known label content of metabolites is added to assist in metabolome annotation (or quantification) (de Jong et al. 2012; Wang et al., 2019), the labelling patterns of metabolites are not known—and are not predictable—in tracing studies of metabolites. Indeed, they represent the desired information to elucidate metabolic pathways. According to the isotopic composition of the labelled source and to the operating metabolic pathways, potentially any combination of isotopologues can be generated for each metabolite in such experiments, which means that the complete isotopic envelope has to be measured to get valuable metabolic information. Moreover, the isotopologue abundances are determined by the pathways activities and can be exploited to measure metabolic fluxes. Hence, untargeted MS-based isotopic tracing studies of metabolism can be defined as the quantitative measurement of the complete isotopic envelope of all detected metabolites. It currently represents a major challenge in terms of MS data processing and interpretation because both metabolites and their labelling patterns are not known. Some software tools have been recently introduced to perform automated extraction of isotopic clusters in untargeted MS-based isotopic tracing studies, but due to the high complexity of the MS data collected in such studies, specific strategies to optimize the parametrization of these tools are required. In this work, a methodology to optimize the extraction of complete isotopic envelopes of all metabolites detected in full-scan MS spectra of labelled samples is introduced. The raw MS data in these experiments are processed in two steps: (1) extraction of individual isotopologues and (2) grouping of individual isotopologues into isotopic clusters. The proposed strategy for optimizing data processing in this context is shown in Fig. 1. The key feature is the addition to the analytical batch of a reference isotopically labelled sample to optimize data processing parameters. The labelling data of a set of metabolites are manually extracted from the MS data of the reference material to generate a reference dataset and the processing parameters are then optimized by minimizing the difference between this reference dataset and the data extracted for the reference metabolites. The same reference dataset is used to optimize the isotopologue extraction and isotopologue clustering steps.

The proposed optimization process is generic and can be applied to various stable isotope tracers used to investigate metabolism. Its use is demonstrated here for ^13^C-tracing, which is the most widespread approach in isotopic studies of metabolism (Wiechert et al., 2001; Zamboni et al., 2009). As a test case to illustrate the application and relevance of the proposed optimization strategy, a ^13^C-labelling experiment was performed in which two *E. coli* strains (wild-type BW25113 and its *∆zwf* derivative knocked-out for the gene encoding the first committed step of the pentose-phosphate pathway) were grown in the presence of ^13^C-labelled glucose as sole carbon source. The intracellular metabolites were sampled at mid-exponential growth and analysed by LC–MS. A reference material was analysed together with the biological samples to optimize the data processing. The reference material and its use for data optimization are described in detail in the following sections. To properly evaluate data quality throughout the optimization process, all samples (including the reference material) were produced and analysed in five replicates. In keeping with the requirements of the ^13^C-profiling software furthermore, unlabelled samples (five replicates) of the reference material and of the *E. coli* strains were produced and analysed in the same analytical batch as the labelled samples.

### Definition of reference sets for optimization

#### Choice of the reference material

Various isotopically labelled materials can be used, provided they satisfy a number of criteria related to the analytical method, the analysed samples, and the biological question to be addressed. The *reference material* should ideally have an identical or similar matrix to the samples of interest to generate the same matrix effects in the MS experiments and contain the same metabolites. It is very important for the labelling patterns of the metabolites to be known or be fully predictable to provide reliable reference data for the optimization process.

The reference material used here was a so-called Pascal triangle (PT) sample. PT samples are biologically produced materials whose isotopic composition is designed to obtain metabolites with tracer isotopologue distributions that match the binomial coefficients of Pascal's triangle. Details about these samples and their application to MS-based isotopic tracing studies can be found in Millard et al. (2014), Heuillet et al. (2018) and Schwaiger-Haber et al. (2019). PT samples were used here for several reasons. First, the fact that the sample could be produced by cultivating *E. coli* on ^13^C-labelled acetate and collecting intracellular polar metabolites, meant that it had exactly the same matrix as the biological samples to be analysed. Second, the chosen PT sample satisfies many of the above-mentioned criteria for reference materials, including a broad metabolome coverage, fully predictable labelling patterns and broad coverage of the isotopologue space (all tracer isotopic forms of the same metabolite are present at the same abundance).

#### Definition of the reference dataset

The *reference dataset* corresponds to analytical data manually extracted from the *reference material* for a list of selected metabolites (the *reference metabolites*) and used as reference data during the optimization process. As for the *reference material*, various sets of metabolites can be used. The *reference metabolites* should be sufficient in number to cover the metabolome. They should be known compounds so that their labelling patterns can be extracted in a targeted fashion and complete isotopic clusters should be reliably detected in the reference material to optimize isotopologue recovery and isotopologue grouping. Note that the *reference metabolites* do not necessarily have to occur in the biological samples for the data optimization process itself since this depends only on the data from the *reference material*. However, they should be selected for their relevance to the objectives of the study.

For this case study, we selected 25 metabolites that are representative of the central metabolism of *E. coli* and are known to be reliably detected with our analytical method. All of them are confirmed level 1 annotated metabolites (Creek et al., 2014). The complete list of the selected *reference metabolites* is given in the Material & Methods (Sect. 2.3.1). This set of metabolites was consistent with the metabolite content of the labelled reference material and the biological question for the *E.coli* strains considered in this work.

According to the elemental formula of the 25 *reference metabolites*, the *reference dataset* should consist of 25 isotopic clusters containing 184 tracer (carbon) isotopologues in total. By manually processing the MS data collected for the PT sample using the software Emzed (Kiefer et al., 2013), all 25 isotopic clusters were found, along with 181 tracer isotopologues (Table S1). The missing isotopologues corresponded to MS signals that were either undetected (CDP M0 and Mn) or with too low S/N ratio (G1P Mn).

The *reference dataset* was further characterized for the mass accuracy and RT isotopic deviation of individual isotopologues. Compared to their theoretical values the mean mass error was 1.42 ± 1.1 ppm for the 181 isotopologues. For the 25 reference metabolites, the RT isotopic deviation ranged from zero to 3 s with a mean relative error of 0.02% across the complete analytical run. These results indicate that the analytical characteristics (m/z, RT pairs) of the detected isotopologues are fully consistent with the values expected for the selected metabolites.

The experimental CIDs of the corresponding metabolites were calculated from the *reference dataset* to generate reference values (*reference CIDs*) (Table S1), which were validated by comparing them with predicted values for the PT sample. The CIDs measured manually for all 25 metabolites deviated by less than 5% on average from the predicted values (Fig. S2).

These results highlight one of the benefits of using a reference material such as the PT sample to optimize processing, namely that analytical problems—limited sensitivity in this case—can be identified and considered separately from processing issues.

The 181 isotopologues in the reference dataset are referred to hereafter as the *reference isotopologues.*

### Optimization of isotopologue extraction

**Fig. 2:**
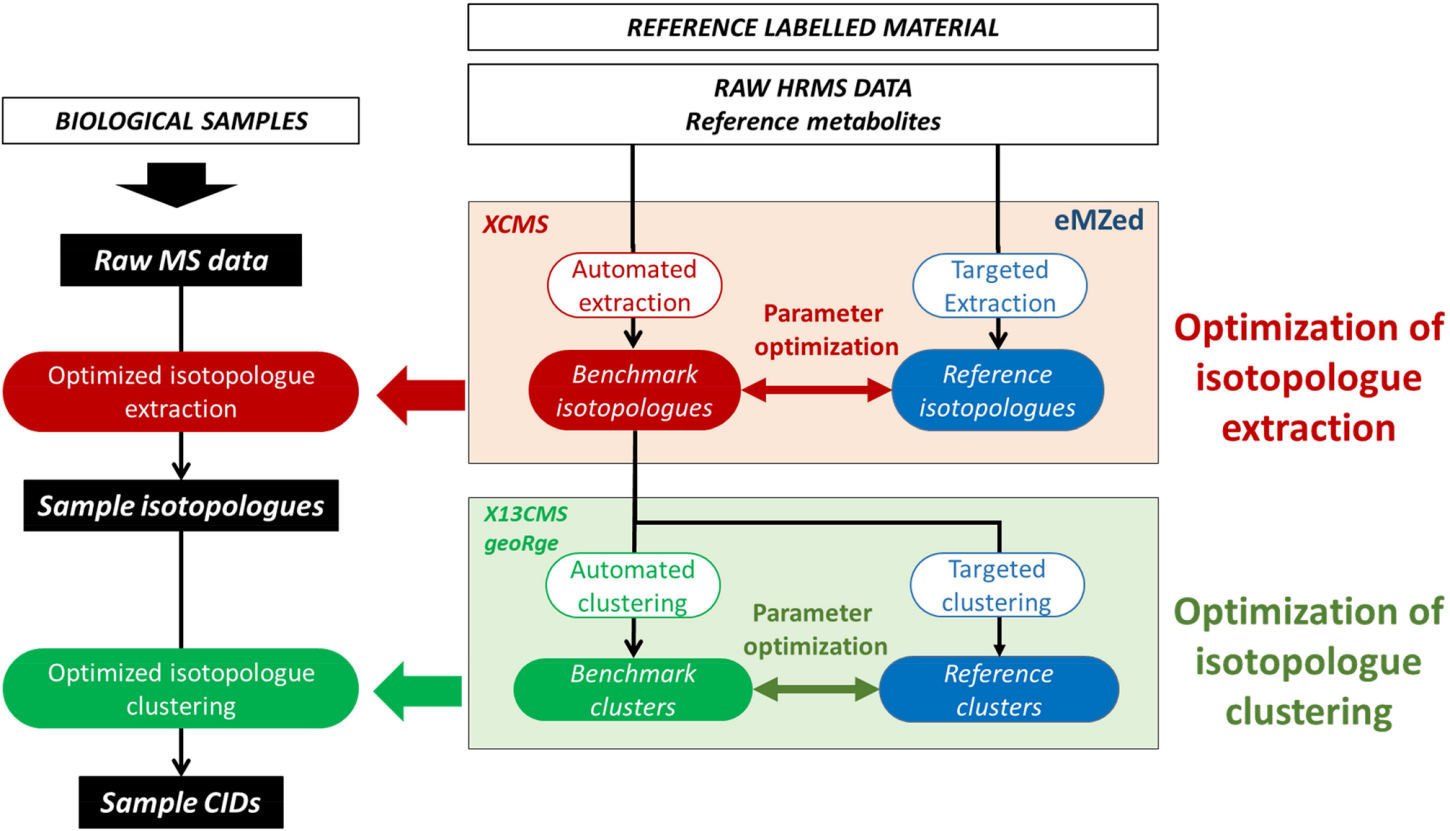
Proposed strategy for the two-step optimization of data processing in untargeted MS-based isotopic tracing studies

The proposed strategy for data processing optimization based on a reference material is illustrated in Fig. 2 and involves two steps (i) optimization of isotopologue extraction and (ii) optimization of isotopologue clustering, as described in detail below. Briefly, in the first step, the *benchmark isotopologues* of the *reference metabolites* are identified automatically using extraction software (XCMS in this work) and are compared to the *reference isotopologues* using three evaluation criteria (recovery rate, analytical characteristics, and isotopologue integrals) and the extraction parameters are then iteratively modified to minimize the difference between the two isotopologue datasets.

#### Starting the optimization process

Some tools were recently published to perform automated parametrization of software in untargeted metabolomics (Libiseller et al., 2015; Manier et al., 2018). Because isotopologue extraction is performed with the same tools as feature extraction in untargeted metabolomics—i.e. XCMS in this study-, such tools can be also applied to untargeted isotopologue analysis. In this work we used the tool IPO (Alboniga et al. 2020; Libiseller et al., 2015), which was specifically designed to parameterize XCMS, to provide starting parameter settings for isotopologue extraction. In compliance with the IPO guidelines, this was done with MS data collected on unlabelled samples—i.e. the unlabelled PT samples. The so-obtained parameters (Table S2) were applied to extract the isotopologues from the labelled PT samples (*benchmark isotopologues*). A total of 164 out of the 181 *reference isotopologues*, were retrieved at the end of this process (Fig S3a). Closer inspection of the data (Table S5) showed that the mass of the extracted peak differed significantly from that of the corresponding peak in the reference data (Table S4). Indeed 22 isotopologues showed mass errors above 3 ppm, the error being higher than 5 ppm for 8 of them, and up to 18.8 for malate M2. This mass discrepancy, together with the fact that 17 isotopologues were not detected at all, indicates that the IPO-defined parameter settings were not optimal for isotopologue extraction. Such results can be explained because IPO was designed to optimize the processing of MS data collected on unlabeled material. The processing of MS data collected on labeled material, which are much more complex (more peaks with lower intensities), requires specific optimization tools. The tool IPO was found useful to provide a first set of parameter values which could be used to as a starting point to evaluate the benefit of the proposed optimization strategy.

#### Manual parameter selection

The XCMS parameters were next optimized using a semi-manual approach depicted in Fig. 2. The IPO parameters (see Materials and Methods, Table S2) were used as a starting point for this process, but other tools or starting values could also have been used. In each optimization round, the isotopologues from the 25 *reference metabolites* were automatically extracted using XCMS and gathered into *benchmark isotopologues*. The *benchmark isotopologues* were then compared to the *reference isotopologues* using the evaluation criteria mentioned above. The process was then iterated after changing the extraction parameters values to maximize the agreement between the *benchmark isotopologues* and the *reference isotopologues*.

This optimization process was used for the five labelled PT samples in the analytical batch. Table S2 lists the parameter settings giving the optimal isotopologue recovery by automated extraction accross the five PT sample replicates (Fig. S3b). The optimized parameters allowed the recovery of 174 isotopologues, i.e. 10 more than with the initial parameter settings. This gain in recovery was accompanied by a gain in data quality (Table S5). The average error in mass accuracy over the common detected isotopologues (for 161 isotopologues) was 1.29 ± 1.02 ppm, to be compared to 1.74 ± 2.38 ppm in the initial data. The lower standard deviation on the mass errors indicated a higher precision of isotopologue masses after optimization. The RT accuracy for the *benchmark isotopologues* compared to the *reference isotopologues* was 0.29 ± 0.22 s on average. The results are given in full in the Supplementary Data (Table S5) and highlight the improvement in data extraction afforded by the proposed optimization strategy.

Nevertheless, seven reference isotopologues remained undetected in the optimized dataset, indicating that the automated process was slightly less efficient than manual extraction. Five of the missing isotopologues, the M0 and Mn of ADP and GDP and the Mn_-1_ of CDP, were not detected in any of the five PT sample replicates. The two others missing isotopologues (UDP M0 and CDP M1) were detected in only one replicate. The chromatographic signal appeared more intense in this replicate than in the others. Overall, the above data indicated not only that the number missed isotopologues was decreased after optimization process, but also that the detected isotopologues were much better defined.

#### Quality of isotopologue quantification

In isotopic studies of metabolism, valuable quantitative information on biochemical pathways is obtained from isotopologue abundances. The reliability of isotopologue quantification is a major issue at the data acquisition level because ionization problems and matrix effects mean that MS is not inherently quantitative. Methods for validating MS methods for reliable isotopologue measurements—including the benefits of using PT samples for such a purpose—as discussed recently by Heuillet et al. (2018) and Schwaiger-Haber et al. (2019), are beyond the scope of this work. Isotopologue quantification can also be limited by data processing. Several factors can be problematic, but the main limitation is the capability of the processing software to properly integrate the MS signals. Optimizing data processing in untargeted isotopic tracing studies therefore also means ensuring isotopologue abundances are properly measured.

Because manual integration is somewhat arbitrary and automatic integration is imperfect regardless of the algorithm considered, the quality of isotopologue quantification was controlled and maximized throughout the optimization process by comparing isotopologue abundances in the *benchmark isotopologues* to those in the *reference isotopologues*. Two methods were used to evaluate quantification accuracy.

We first compared the absolute abundances of individual isotopologues separately. Figure S4 shows that the *benchmark isotopologues* have absolute abundances very close to those in the reference dataset. The isotopologue areas were closely similar whether integrated manually or automatically, indicating the reliability of automated isotopologue quantification after optimization. Some slight overestimations were observed for two metabolites showing noisy peaks (succinate and malate), while individual isotopologues with very low S/N (lightest and heaviest isotopologues of G1P, Sed7P) were underestimated.

**Fig. 3:**
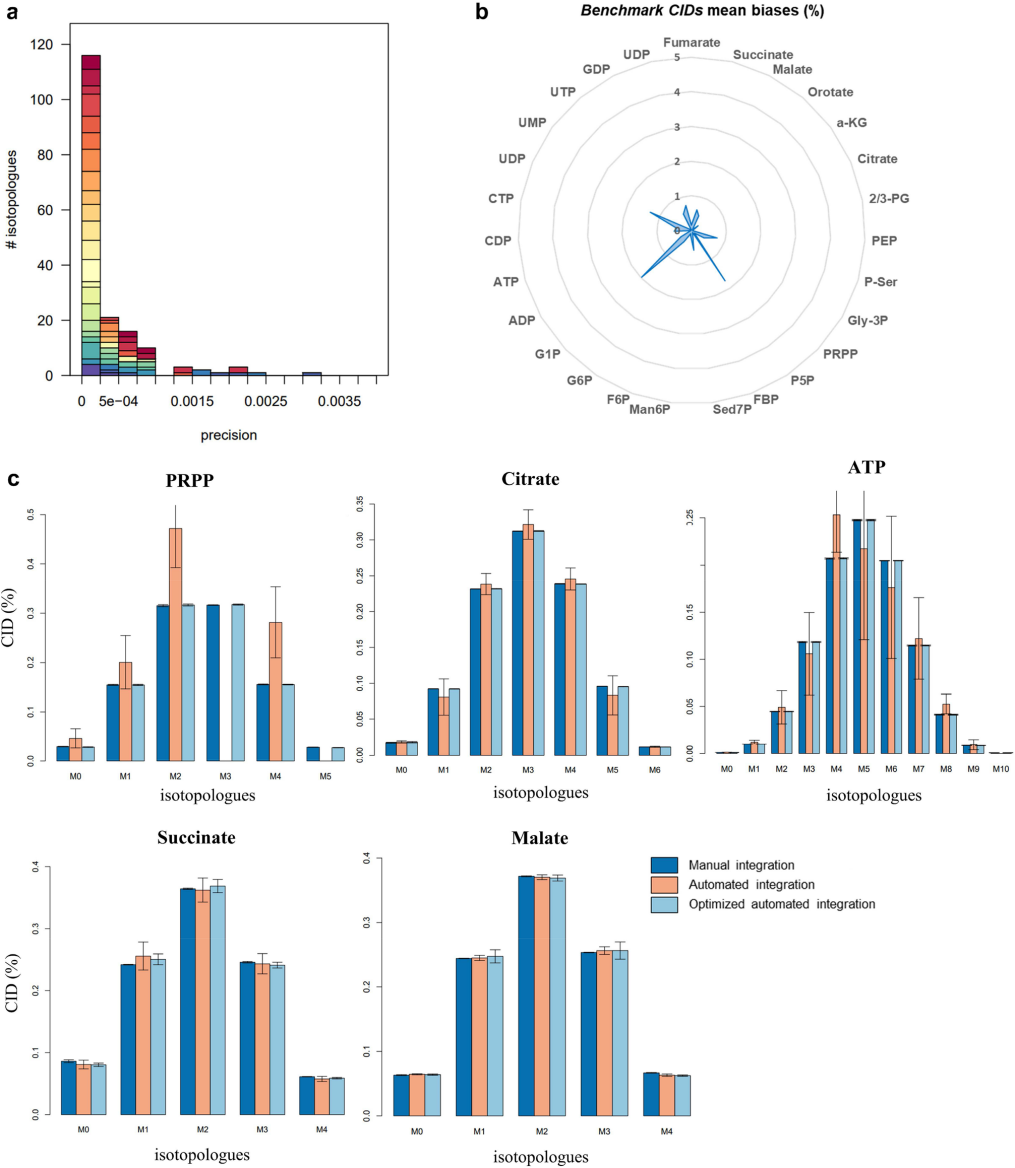
Impact of parameter optimization on the measurement of isotopologue abundances. **a** Distribution of precision for the 174 XCMS-extracted *benchmark isotopologues* in the PT sample. **b** Mean biases (%) of optimized *benchmark CIDs* with respect to *reference CIDs.* **c** Comparison of *reference CIDs* (dark blue), IPO *benchmark CIDs* and optimized *benchmark CIDs* (light blue) for PRPP, citrate, ATP, malate and succinate in the PT samples

The mean SD in integrated areas across the five replicate measurements was 0.0003 for the 174 extracted isotopologues (Fig. 3A), emphasizing the high quantitative reliability of the automatic isotopologue extraction process. We next compared the isotopologue abundances relative to the isotopic cluster of the corresponding metabolite by calculating the CIDs. Isotopologue quantification errors propagate to the entire CID vector, so that comparing CIDs calculated after automated extraction to manually measured CIDs is a sensitive method of detecting processing-induced quantification errors.

*Benchmark CIDs* were calculated for the *benchmark isotopologues*—before (i.e. with IPO settings) and after parameter optimization—after reconstructing molecular isotopic clusters and correcting for naturally-occurring isotopes. The *benchmark CIDs* were then compared to the *reference CIDs*. The data are shown in full in the Supplementary Data (Fig S5). The results obtained with the final, XCMS-optimized dataset are shown in Fig. 3B, C. For all 25 metabolites, the CIDs obtained after parameter optimization were in close agreement with the reference values (average error below 2%; Fig. 3B). Figure 3C compares the CIDs of selected metabolites with reference values before and after parameter optimization. The CIDs calculated from the initial non-optimized dataset are generally biased and show significant inter-replicate variability (e.g. PRPP, Fig. 3C). This is partly because many isotopologues go undetected with the IPO parameters, as mentioned above. After parameter optimization however, the CIDs were in good agreement with the corresponding values in the *reference dataset*, showing the benefit of the proposed optimization strategy. Interestingly, the CIDs of both malate and succinate, whose isotopologue abundances were overestimated in the optimized dataset (Fig. S4), were also closely consistent (Fig. 3C). This means that although the MS signals of the two compounds were overestimated in the automatically extracted data, the quantitative relationships between isotopologues of the same compound were preserved. This observation points to a potential bias in interpreting the abundances of individual isotopologues from different metabolites to derive quantitative metabolic information—e.g. comparing the M + 5 isotopologue of citrate to the M + 5 isotopologue of glutamine to determine reductive glutamine metabolism –without considering all potential data acquisition and processing problems.

Altogether, the above results clearly emphasize the significant improvement in the quality of the quantitative data achieved through the proposed optimization strategy. The results also show that data processing can be a substantial source of bias in MS-based untargeted isotopic tracing investigations, in terms of the number, correctness and quantification of the recovered isotopologues.

### Optimization of isotopologue clustering

Isotopologue clustering consists in the grouping of extracted isotopologues into metabolite isotopic clusters (Fig. 2). Two different programs, geoRge and X13CMS (Patti et al. 2014; Capellades et al., 2016; Dange et al., 2020), were used to do this. Clustering was optimized in a similar fashion as the extraction process was (Fig. 2). The isotopic clusters of the 25 reference metabolites (*reference clusters*) were manually extracted from the optimal set of isotopologues. The software were used to automatically extract the 25 clusters (*benchmark clusters*) from the same dataset. The optimization consisted in adjusting software parameters to minimize the difference between benchmark clusters and reference clusters.

The quality of clustering was evaluated from the proportion of correct clusters that were recovered. A correct cluster was defined as containing only all the correct isotopologues. Two types of incorrect cluster were considered: incomplete clusters, missing one or more isotopologues, and corrupt clusters, with one or more spurious isotopologues. We defined two figures of merit to optimize based on the proportions of correct and incorrect clusters in the *benchmark clusters*: recall, or sensitivity, the ability to detect a cluster for all 25 reference metabolites; and precision, the number of correct clusters retrieved in the *benchmark clusters*. The software parameters were then iteratively modified to maximize the recall and the precision of the *benchmark clusters*.

Preliminary tests showed that the quality of the clustering depended mainly on two parameter (isotopologue mass deviation and RT window). The isotopologue mass deviation (“ppm” for X13CMS and “ppm.s” for geoRge) is the acceptable error in m/z measurements between successive isotopologues in the same isotopic cluster (the accuracy of isotopic distances), which should be the mass difference between ^13^C and ^12^C (1.00335 m/z). The RT window (“RT window” for X13CMS and “rt.win.min” for geoRge), corresponds to the tolerance on the RTs of isotopologues from the same metabolite, which should in theory be exactly the same. The RT deviation was measured to vary between 0.2 and 7.2 in the isotopologue dataset obtained after XCMS optimization (Table S5, see 3.3.2). From these values, two different RT windows (5 and 10 s) were considered and the isotopologue mass deviation was varied from 1 to 10 ppm. The noise threshold was deliberately set at a low value (5000) to maximize peak extraction.

As reported previously (Dange et al., 2020), many redundancies were observed in the clusters obtained with geoRge independently of the parameters used. This is due to the clustering algorithm of geoRge, which generates various clusters from the same set of isotopologues. The optimization for geoRge was therefore performed after manual curation of obvious redundancies in the geoRge dataset.

**Fig. 4:**
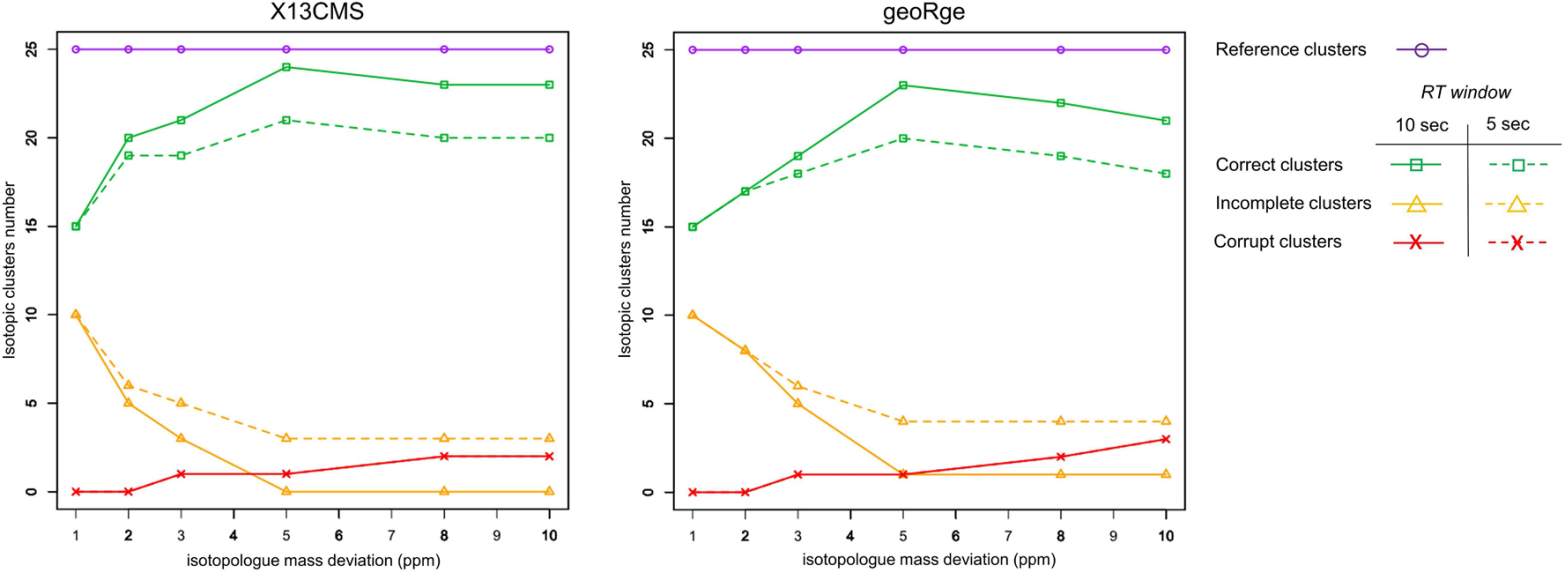
Quality of isotopologue clustering. Number of correct (green line), incomplete (orange line) and corrupt (red line) isotopic clusters detected by X13CMS and geoRge depending on the isotopologue mass deviation (1, 2, 3, 5, 8 or 10 ppm) and the RT window (5 s, dotted line; or 10 s, solid line) based on the set of *reference clusters* (purple line) for the five replicates of the PT sample

For both programs, a low mass deviation threshold produced more incomplete clusters while increasing the mass deviation generated more corrupt clusters (Fig. 4). The missing species were most often the Mn isotopologues of nucleotides (ADP, ATP, CTP, GDP, UDP, UMP, UTP), which can be explained by the lower quality of the XCMS data for these species and their larger mass deviation (see Sect. 3.3 and Table S4). Processing the data with a larger mass tolerance allowed these isotopologues to be recovered but also tended to generate corrupt clusters.

An RT window of 10 s was found to yield a greater proportion of correct clusters than an RT window of 5 s for all isotopologue mass deviation values except 1 ppm, for which the proportion of correct clusters was the same with both. The number of corrupt clusters did not depend on the length of the RT window, regardless of the mass deviation used. These results are because while unlabelled and labelled samples were processed simultaneously, the heaviest isotopologues were only detected in the ^13^C-enriched samples leading to a certain amount of variation in RTs.

These results are based on a targeted search of 25-*benchmark clusters* and we assume that the observed errors are representative of the clustering process for the entire dataset. Table 1 shows that with an RT window of 10 s, the precision and recall were optimal with both programs at a mass deviation of 5 ppm. The equivalent results for an RT window of 5 s are provided in Table S6.

**Table 1:** Cluster precision and recall for X13CMS and geoRge with a RT window of 10 s and different isotopologue mass deviations, evaluated for the 25 *reference metabolites* in the PT sample

	Isotopologue mass deviation (ppm)	1 (%)	2 (%)	3 (%)	5 (%)	8 (%)	10 (%)
X13CMS	Precision	60	80	84	96	92	92
	Recall	100	100	100	100	100	100
geoRge	Precision	60	68	76	92	88	84
	Recall	100	100	100	100	100	100

The recall of both programs was 100%, meaning that all 25 *reference metabolites* were retrieved. Almost all these clusters were correct, with a precision of 96% (1 incorrect cluster) and 92% (2 incorrect clusters) for X13CMS and geoRge, respectively. The ATP cluster was found incorrect with both programs, with some isotopologues wrongly identified by the software as M35 to M39. The second incorrect cluster for geoRge was ADP, whose M9 isotopologue was missed because of the statistical rules applied by geoRge to select potential enriched isotopologues in the labelled samples. Close inspection of the data for this isotopologue showed that some noise had been integrated for the unlabelled samples, and could be interpreted as signal by geoRge, so that the M9 peak in the labelled data was not considered as labelled.

These results show that both programs perform well despite their slightly different approaches. Briefly, geoRge compares potential isotopic peaks in the labelled and unlabelled samples with all candidate basepeaks within the vector of masses calculated for each potential isotopologue. On the other hand, X13CMS compares all potential isotopologue peak pairs within a RT bin, groups them together based on a common basepeak and discards duplicate information. In this targeted search of 25 *reference metabolites*, X13CMS generated a smaller number of incorrect clusters than geoRge and no correction for clustering redundancy was required. It has been shown that in spite of these redundancies, geoRge tends to generate fewer false positives than X13CMS, but can miss some features that X13CMS finds (Capellades et al., 2016; Dange et al., 2020). The above results show that regardless of the software chosen, independently optimizing the parameters used to group isotopic clusters is essential.

### Application to the case study

To illustrate its use, the optimization workflow was applied to the study of wild type *E. coli* BW 25113 and a mutant deleted for the *zwf* gene (∆*zwf*) that encodes glucose-6-phosphate dehydrogenase (G6PDH). This mutation has a negligible impact on the growth of the bacterium but leads to metabolic adaptations, which can be nicely revealed by using ^13^C-labelling experiments (Nicolas et al., 2007; Zhao et al., 2004; Bergès, Cahoreau et al. 2021). We used this example of an untargeted MS based isotopic tracing investigation to illustrate how the proposed workflow optimizes the recovery of this kind of labelling information.

The *E. coli* samples were analysed by LC–MS and first processed using the starting (IPO-derived) parameter settings (Table S2). Then data processing was repeated with the optimal parameter settings (Table S2). The gain in data quality resulting from the optimization is illustrated in for the WT strain (Table 2). The fact that the proposed approach yields the same cluster precision for biological samples as for the reference material, confirms the efficiency of the optimization process.

**Table 2:** Impact of parameter optimization on the extraction of data from the *E. coli* WT samples

Clustering software	Cluster precision (%)	
	Starting parameter settings (%)	Optimized parameter settings (%)
X13CMS	40	96
geoRge	44	92

Cluster precision (%) of the two programs for the 25 reference metabolites before and after parameter optimization

**Fig. 5:**
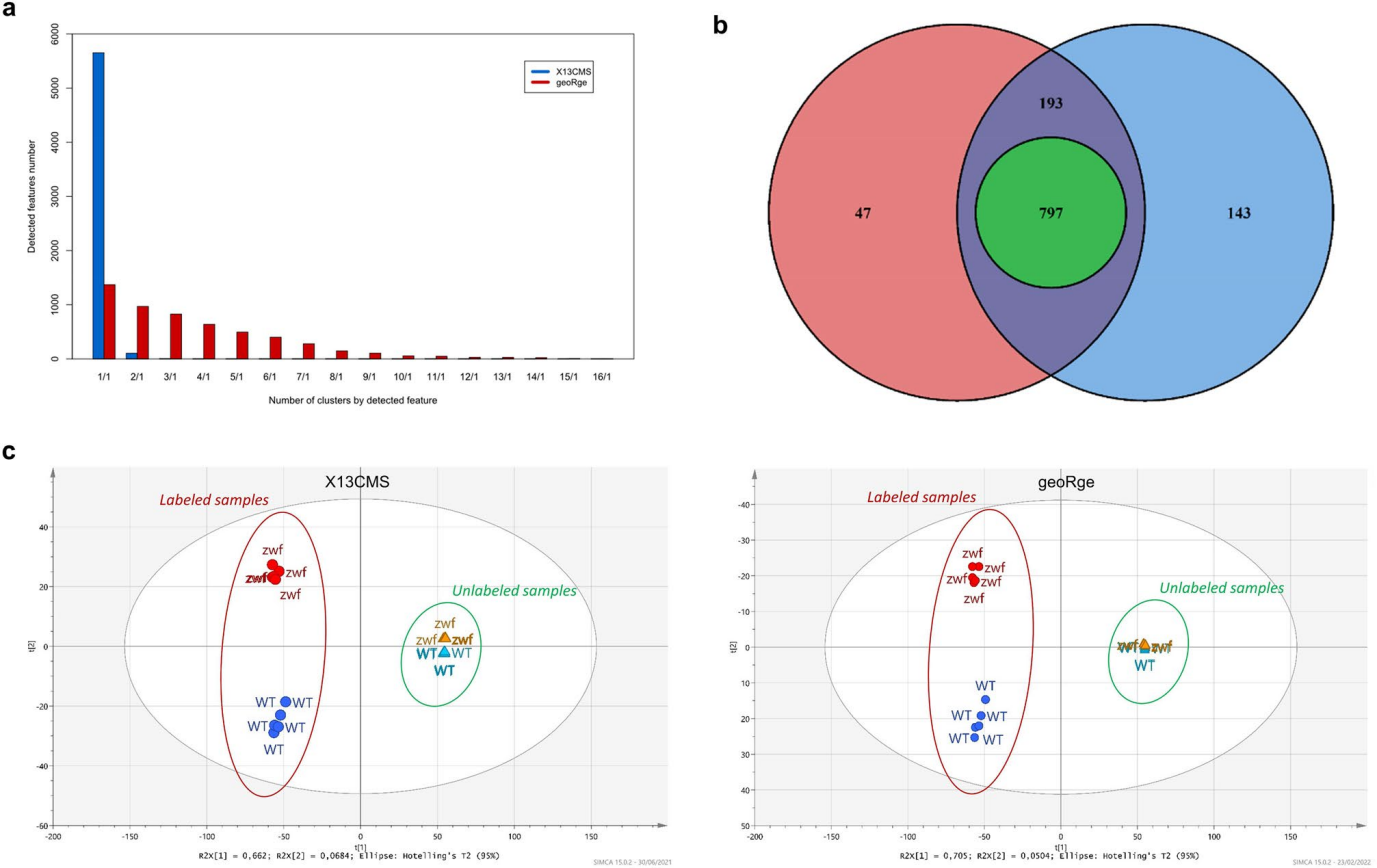
**a** Comparison of the number of isotopic clusters each detected isotopologue appears in for X13CMS (blue) and geoRge (red) software. **b** Venn diagram of the number of isotopic clusters detected by X13CMS (blue) and geoRge (red) in biological samples, including clusters identical both in length and isotopologue composition (green circle). **c** PCA plots of the extracted isotopic profiles of unlabelled and labelled wild-type and *∆zwf E.coli* strains after processing using X13CMS (left) and geoRge (right)

In total, 10,129 isotopologues were extracted by XCMS from the two *E. coli* strains and were further processed with geoRge and X13CMS to group some of isotopologues in isotopic clusters. As already observed, geoRge generated a significant number of redundancies from this dataset compared to X13CMS (Fig. 5A), but after manual curation the number of clusters was similar with both software (1037 and 1133 for geoRge and X13CMS, respectively) (Fig. 5B). Over all the distinct clusters detected (1180) with both programs (Fig. 5B), a total of 990 clusters had the same basepeak, corresponding to an overlap of 84%, of which 797 were identical in both cluster length and isotopologue composition (Table S7).

Regarding the greater number of clusters detected by X13CMS than by geoRge, the 190 isotopic clusters identified by one program and not the other (147 by X13CMS and 43 by geoRge) mostly contained a single isotopologue possibly because of instrumental noise or the presence of unlabelled metabolites or other unresolved peaks.

In isotope labelling experiments, isotopologue abundances can be interpreted either individually (*e.g.* the evolution of the M + 3 peak intensity of a metabolite) or relative to the complete isotopic cluster (isotopologue distribution), the latter approach being the most common way of describing labelling patterns in ^13^C-fluxomics. Here, mass fractions were calculated for all detected clusters in all the samples (unlabelled and labelled) from the outputs of X13CMS and geoRge, and PCA was used to explore differences in the isotopic profiles of two *E.coli* strains (Fig. 5C). Comparisons of isotopic profiles depend heavily on the number of features detected and how they are clustered, therefore on the quality of the data processing. The PCA plots for the two programs show a similar level of separation between the different biological conditions, confirming the repeatability of the workflow from sample preparation through to data processing. In these plots, labelled and unlabelled samples are strictly separated along the first PCA component and WT and ∆*zwf* strains along the second. The lack of separation between the two groups of unlabelled samples is expected because the unlabelled mass fractions have the same isotopomer composition (i.e. natural abundance), such that the only discriminating factor is the presence or absence of peaks. On the contrary, the ^13^C-enriched samples are closely grouped by strain on the plots according to their isotopic composition. This demonstrates the significant impact of the ∆*zwf* mutation on flux distribution, which then significantly affects the isotopic composition of the metabolites.

The WT and ∆*zwf* groups were analysed to identify the most discriminating labelling data between the two strains. The corresponding isotopic clusters were compared using Wilcoxon tests, with 207 (X13CMS) and 138 (geoRge) of these clusters having more than one significantly different (*p* ≤ 0.025) isotopologue between strains. By exploiting an in-house database (containing 47 metabolites), 20 isotopic clusters could be assigned to metabolites with a level 1 confidence (Creek et al., 2014). They were related to glycolysis (Fumarate, Succinate, Malate, 2/3-PG, PEP, G6P, FBP), the PPP (Sed7P, Orotate, P5P, Shiki3P (CAS: 63959-45-5)) and nucleotide biosynthesis (ADP, ATP, CDP, CTP, UMP, UDP, UTP, UDP-Glucose (CAS: 133-89-1), UDP-Acetylglucosamine (CAS: 528-04-1)) (Fig. S7). Changes in the labelling patterns of these metabolites was fully consistent with the modifications expected for the ∆zwf strains, which is known to significantly impact the partition between glycolysis and the PPP (Nicolas et al., 2007; Zhao et al., 2004), resulting also in differential labelling of the ribosyl moiety of nucleotides. Furthermore, the number of significantly different isotopic clusters that remain unidentified after this initial analysis demonstrates the power of the untargeted approach and the need for further identification.

## Conclusion

This work emphasized that specific workflows have to be developed for optimal processing of the complex MS data that are generated in MS-based untargeted isotopic tracing studies of metabolism. Indeed, the results showed that significant gain in the recovery of valuable information was obtained by applying the proposed methodology for data processing optimization. The application of a suitable reference material to optimize software parametrization proved to increase not only the number of recovered isotopic data but also the quality of the data. Pascal Triangle samples are well suited for such purpose since they allow both the identification of analytical issues and optimization of data processing at the same time. Together with the progress in MS instrumentation and analytical methods, which allows to extend the metabolome—and fluxome—coverage, applying the proposed methodology is maximizing the biological value of isotopic tracing investigations by revealing the full metabolic information that is encoded in the labelling patterns of the metabolites.

## Supplementary Information

Below is the link to the electronic supplementary material.

Supplementary file1 (PDF 2707 kb)

## Abbreviations

2/3-PG: 2 And 3-phosphoglycerate
ACN: Acetonitrile
a-KG: Alpha-ketoglutarate
ATP: Adenosine triphosphate
ADP: Adenosine diphosphate
CID: Carbon isotopologue distribution
CDP: Cytosine diphosphate
CTP: Cytosine triphosphate
F6P: Fructose-6-phosphate
FBP: Fructose-1,6-diphosphate
G1P: Glucose-1phosphate
G6P: Glucose-6-phosphate
GDP: Guanosine diphosphate
Gly3P: Glycerol-3phosphate
LC/MS: Liquid chromatography with mass spectrometry
Man6P: Mannose-6-phosphate
MeOH: Methanol
P5P: Pentose-5phosphate
PCA: Principal component analysis
PEP: Phosphoenolpyruvate
PRPP: 5-Phosphoribosyl-pyrophosphate
PT: Pascal triangle
RT: Retention time
Sed7P: Sedoheptulose-7phosphate
UDP: Uridine diphosphate
UMP: Uridine monophosphate
UTP: Uridine triphosphate

## Glossary

Benchmark clusters: Dataset containing the benchmark isotopologues automatically clustered using the two clustering software
Benchmark isotopologues: Dataset containing isotopologues of *reference metabolites* automatically extracted from MS data of the reference material using XCMS
Isotopic cluster: Group of MS peaks from a unique molecular entity, i.e. with the same elemental composition but different isotopic compositions (IUPAC definition)
Isotopologues: Molecular entities that differ only in their isotopic composition (IUPAC definition)
Pascal triangle (PT) sample: Biologically-produced material in which the isotopic composition of the labelled substrate is designed to obtain metabolites with tracer isotopologues distributed according to the binomial coefficients of Pascal's triangle
Reference clusters: Reference dataset containing the benchmark isotopologues manually clustered
Reference isotopologues: Reference dataset containing isotopologues of *reference metabolites* manually extracted from MS data of the reference material
Reference material: Labelled sample used as a reference to optimize processing parameters
Reference metabolites: List of metabolites identified with a level 1 confidence expressed and measurable in the reference material
Tracer isotopologues: Isotopologues of the tracer element
