# Supplementary material for: An optimization method for untargeted MS-based isotopic tracing investigations of metabolism
Source: Metabolomics. 2022 Jun 16;18(7):41. doi: 10.1007/s11306-022-01897-5 (PMC9205802; doi:10.1007/s11306-022-01897-5)
Supplement: Supplementary file 1 — Supplementary file1 (PDF 2707 kb) [file 11306_2022_1897_MOESM1_ESM.pdf]

Supporting information

An optimization method for untargeted MS-based isotopic tracing investigations of metabolism

Noémie Butin[1,2,3], Cécilia Bergès[2,3], Jean-Charles Portais[1,2,3], Floriant Bellvert[2,3,*]

[1] RESTORE, Université de Toulouse, CNRS ERL5311, EFS, ENVT, Inserm U1031, UPS, Toulouse, France

[2] Toulouse Biotechnology Institute, TBI-INSA de Toulouse INSA/ CNRS 5504-UMR INSA/INRA 792, Toulouse, France

[3] MetaboHUB-MetaToul, National Infrastructure of Metabolomics and Fluxomics, Toulouse, 31077, France

*corresponding author: floriant.bellvert@insa-toulouse.fr

Table of contents

Figure S-1. Conversion of raw MS data with MSConvert

Table S-1. Reference datasets.

Table S-2. Optimized XCMS automated peak extraction and integration parameters from the Pascal triangle sample.

Table S-3. Ranges for the parameter optimization.

Table S-4. Optimized parameters for ^{13}C-clustering software.

Figure S-2. Reference CIDs mean biases.

Figure S-3. Percentage of recovery in datasets**.**

Table S-5. Mass accuracies of XCMS datasets.

Figure S-4. Impact of parameter optimization on the measurement of isotopologue abundances.

Figure S-5. CIDs comparison between optimized and non-optimized datasets.

Figure S-6. Clustering software redundancies after manual curation.

Table S-6. Cluster precision and recall for clustering software.

Figure S-7. Carbon mass fractions for significant metabolites between two *E.coli* strains.

Table S-7. Overlapping isotopic clusters.

Figure S-1. Conversion of raw data using MSConvert

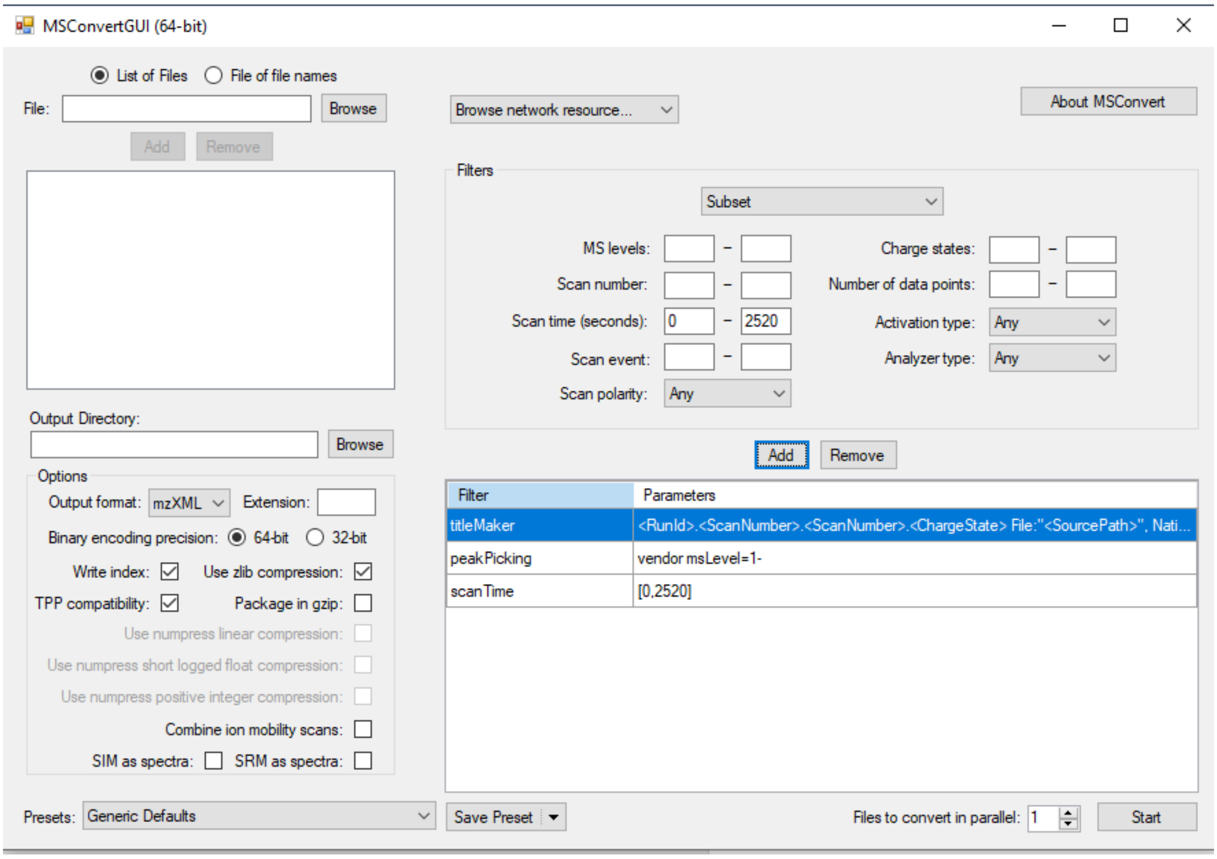

Table S-1. Reference datasets.

Reference metabolites	Reference Isotopologues	m/z	RT (sec)	area	Reference CIDs
**Fumarate**	M0	115.00350	981	56030235	0.07
	M1	116.00685	981	186005277	0.24
	M2	117.01019	981	286367284	0.37
	M3	118.01353	981	195796893	0.25
	M4	119.01686	981	50076785	0.06
**Succinate**	M0	117.01914	667	282803625	0.09
	M1	118.02248	667	793899995	0.24
	M2	119.02576	667	1195649421	0.36
	M3	120.02911	667	806966256	0.25
	M4	121.03250	667	202284443	0.06
**Malate**	M0	133.01417	670	424342051	0.06
	M1	134.01748	670	1626757443	0.24
	M2	135.02079	670	2478580090	0.37
	M3	136.02404	670	1688263347	0.25
	M4	137.02743	670	447848485	0.07
**Orotate**	M0	155.00991	1542	307245998	0.03
	M1	156.01325	1542	1523469272	0.16
	M2	157.01648	1542	3020822605	0.31
	M3	158.01978	1542	3019378130	0.31
	M4	159.02310	1542	1505195603	0.16
	M5	160.02658	1542	287627182	0.03
**a-KG**	M0	145.01417	882	30474657	0.05
	M1	146.01753	882	96355684	0.15
	M2	147.02088	882	195160824	0.30
	M3	148.02424	882	200443971	0.31
	M4	149.02753	882	102259920	0.16
	M5	150.03093	882	21077798	0.03
**Citrate**	M0	191.01994	1813	418678922	0.02
	M1	192.02325	1813	2201958564	0.09
	M2	193.02646	1813	5529085713	0.23
	M3	194.02973	1813	7452410371	0.31
	M4	195.03301	1813	5705670648	0.24
	M5	196.03628	1813	2288719058	0.10
	M6	197.03998	1813	280685227	0.01
**2/3-PG**	M0	184.98577	1741	1606633559	0.12
	M1	185.98906	1741	4951837448	0.37
	M2	186.99233	1741	5048634498	0.38
	M3	187.99559	1741	1678812830	0.13
**PEP**	M0	166.97505	1889	338965384	0.12
	M1	167.97833	1889	1060010283	0.38
	M2	168.98164	1889	1080910744	0.38
	M3	169.98506	1889	341973019	0.12
**Gly-3P**	M0	171.00653	514	60073480	0.21
	M1	172.00990	514	101324423	0.35
	M2	173.01317	514	99069549	0.34
	M3	174.01661	514	30466742	0.10
**PRPP**	M0	388.94405	2154	3773252	0.03
	M1	389.94740	2154	19526130	0.15
	M2	390.95072	2154	39788198	0.32
	M3	391.95403	2154	39994054	0.32
	M4	392.95738	2154	19646953	0.16
	M5	393.96044	2154	3620865	0.03
**P5P**	M0	229.01225	1087	15790399	0.03
	M1	230.01552	1087	80560741	0.16
	M2	231.01884	1087	164024774	0.32
	M3	232.02220	1087	165122627	0.32

	M4	233.02558	1087	79802609	0.15
	M5	234.02910	1087	12746690	0.02
**FBP**	M0	338.98810	2082	60911534	0.02
	M1	339.99131	2082	372220250	0.09
	M2	340.99460	2082	929801620	0.23
	M3	341.99782	2082	1247455449	0.31
	M4	343.00107	2082	939764667	0.24
	M5	344.00425	2082	375932623	0.09
	M6	345.00732	2082	545633560	0.01
**Sed7P**	M0	289.03269	1194	4238381	0.01
	M1	290.03604	1194	30245296	0.05
	M2	291.03938	1194	92560049	0.16
	M3	292.04273	1194	156438036	0.28
	M4	293.04608	1194	156671855	0.28
	M5	294.04944	1194	92460730	0.16
	M6	295.05165	1194	28587991	0.05
	M7	296.05471	1194	2996829	0.01
**Man6P**	M0	259.02304	1022	19907444	0.02
	M1	260.02636	1022	118046331	0.10
	M2	261.02961	1022	293341582	0.25
	M3	262.03289	1022	380574277	0.33
	M4	263.03623	1022	263394462	0.23
	M5	264.03964	1022	86609808	0.07
	M6	265.04309	1022	6272360	0.01
**F6P**	M0	259.02307	953	16279740	0.02
	M1	260.02637	953	99556729	0.09
	M2	261.02961	953	255419536	0.24
	M3	262.03289	953	345140729	0.32
	M4	263.03621	953	256612959	0.24
	M5	264.03961	953	96965877	0.09
	M6	265.04305	953	12107622	0.01
**G6P**	M0	259.02303	892	37644409	0.01
	M1	260.02632	892	232144699	0.09
	M2	261.02955	892	600703579	0.24
	M3	262.03284	892	813250693	0.32
	M4	263.03613	892	610245636	0.24
	M5	264.03951	892	232058201	0.09
	M6	265.04295	892	29220183	0.01
**G1P**	M0	259.02294	521	2188513	0.02
	M1	260.02630	521	11459761	0.10
	M2	261.02963	521	28785602	0.25
	M3	262.03295	521	38072046	0.33
	M4	263.03632	521	26003880	0.23
	M5	264.03973	521	7928553	0.07
	M6	-	-	-	0.00
**ADP**	M0	426.02220	2046	2457757	0.001
	M1	427.02540	2046	16922886	0.01
	M2	428.02871	2046	74325863	0.04
	M3	429.03196	2046	199432722	0.12
	M4	430.03521	2046	349509378	0.21
	M5	431.03846	2046	417235612	0.25
	M6	432.04172	2046	343721466	0.20
	M7	433.04502	2046	192069385	0.11
	M8	434.04845	2046	68002000	0.04
	M9	435.05177	2046	13419219	0.01
	M10	436.05448	2046	1474728	0.0008
**ATP**	M0	505.98885	2184	16246587	0.001
	M1	506.99234	2184	121367108	0.01
	M2	507.99573	2184	534430940	0.04
	M3	508.99902	2184	1422686675	0.12

	M4	510.00231	2184	2486164802	0.21
	M5	511.00559	2184	2972343098	0.25
	M6	512.00881	2184	2458060594	0.20
	M7	513.01208	2184	1378870250	0.11
	M8	514.01539	2184	497063414	0.04
	M9	515.01844	2184	104015344	0.01
	M10	516.02078	2184	9917869	0.0008
**CDP**	M0	-	-	-	0.00
	M1	403.01368	1818	6378057	0.02
	M2	404.01712	1818	27717271	0.07
	M3	405.02042	1818	64362251	0.17
	M4	406.02372	1818	95223930	0.25
	M5	407.02705	1818	93347270	0.25
	M6	408.03040	1818	61069389	0.16
	M7	409.03379	1818	24949899	0.07
	M8	410.03702	1818	4902167	0.01
	M9	-	-	-	0.00
**CTP**	M0	481.9776	2106	8538776	0.002
	M1	482.98096	2106	65463726	0.02
	M2	483.98428	2106	256077353	0.07
	M3	484.98759	2106	592625022	0.17
	M4	485.99086	2106	884008506	0.25
	M5	486.99417	2106	870996338	0.25
	M6	487.99749	2106	567941167	0.16
	M7	489.00077	2106	233553217	0.07
	M8	490.00390	2106	54518770	0.02
	M9	491.00624	2106	5699163	0.002
**GDP**	M0	442.01707	2258	1268796	0.004
	M1	443.02045	2258	2757797	0.01
	M2	444.02364	2258	12302899	0.05
	M3	445.02699	2258	32572041	0.12
	M4	446.03027	2258	56954948	0.21
	M5	447.03359	2258	67914235	0.25
	M6	448.03690	2258	55575927	0.20
	M7	449.04030	2258	30545046	0.11
	M8	450.04364	2258	10876038	0.04
	M9	451.04667	2258	2043296	0.01
	M10	452.04925	2258	61934	0.0002
**UDP**	M0	402.99462	2202	21775720	0.002
	M1	403.99807	2202	183070962	0.02
	M2	405.00128	2202	737410758	0.07
	M3	406.00453	2202	1728622632	0.17
	M4	407.00778	2202	2580480660	0.25
	M5	408.01098	2202	2561842283	0.25
	M6	409.01421	2202	1684070366	0.16
	M7	410.01752	2202	691542759	0.07
	M8	411.02084	2202	155155050	0.01
	M9	412.02337	2202	13935933	0.001
**UMP**	M0	323.02759	1931	31372647	0.002
	M1	324.03098	1931	262454720	0.02
	M2	325.03428	1931	1050573013	0.07
	M3	326.03757	1931	2416270973	0.17
	M4	327.04080	1931	3587714566	0.25
	M5	328.04402	1931	3551766195	0.25
	M6	329.04727	1931	2346046321	0.16
	M7	330.05055	1931	984698697	0.07
	M8	331.05388	1931	219836705	0.02
	M9	332.05669	1931	18514492	0.001
**UTP**	M0	482.96185	2208	9341187	0.002
	M1	483.96505	2208	81137802	0.02

	M2	484.96834	2208	319754515	0.07
	M3	485.97165	2208	744990764	0.17
	M4	486.97494	2208	1110932982	0.25
	M5	487.97820	2208	1103056897	0.25
	M6	488.98148	2208	723196293	0.16
	M7	489.98478	2208	300497734	0.07
	M8	490.98783	2208	70319340	0.02
	M9	491.99024	2208	6951686	0.002

Table S-2. Optimized XCMS automated peak extraction and integration parameters from the Pascal triangle sample.

	xcmsSet()				group()			retcor()
**Parameters**	**method**	**mass error**	**peakwidth**	**mzdiff**	**mzwid**	**minfrac**	**bw**	**method**
Starting (IPO) parameters	centWave	10	(24.86)	−0.036	0.013	0.5	5	-
Manually optimized parameters	centWave	10	(28.99)	−0.001	0.0015	0.2	10	peakgroups

Table S-3. Ranges for the parameter optimization. The XCMS parameters and their range of values tested starting with the parameters provided by IPO.

**Parameter**	**Minimum**	**Maximum**
peakwidth minimum	10	30
peakwidth maximum	80	120
ppm	10	10
mzdiff	-0.001	0.002
mzwid	0.001	0.01
bw	5	15

Table S-4. Optimized parameters for 13C-clustering software

Program	Routine	Settings	Optimized value
X13CMS	getIsoLabelReport()	RTwin	10
		ppm	5
		noise	5000
geoRge	PuIncSeeker()	fc threshold	1.2
		p-value threshold	0.05
		Basepeak min intensity	5000
	Basepeak_finder()	Basepeak mass error	5
		RT min win	10

Figure S-2. Reference CIDs mean biases. Mean biases (%) of *reference CIDs* with respect to the predicted values for the 25 *reference metabolites* in the PT samples.

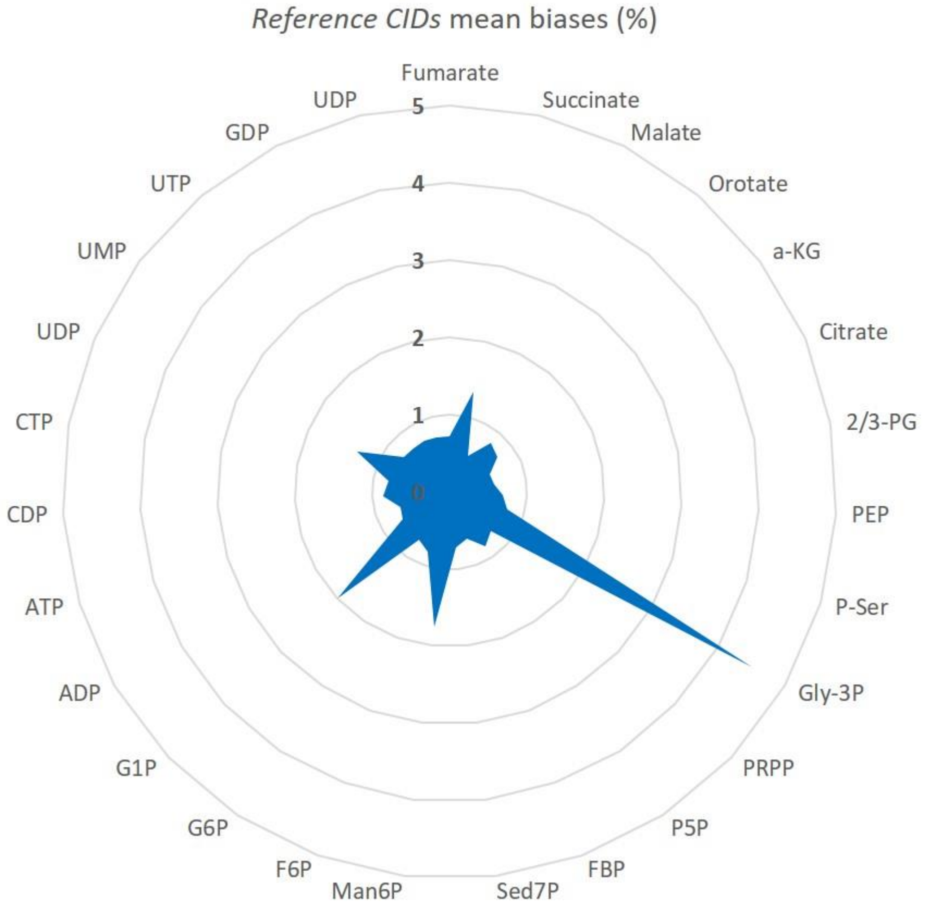

Figure S-3. Percentage of recovery in datasets. Number of isotopologues from *reference metabolites* that were detected or missing in the *reference isotopologues* and the *benchmark isotopologues* after **a.** XCMS processing using starting parameters and **b.** XCMS processing using optimized parameters in the PT samples.

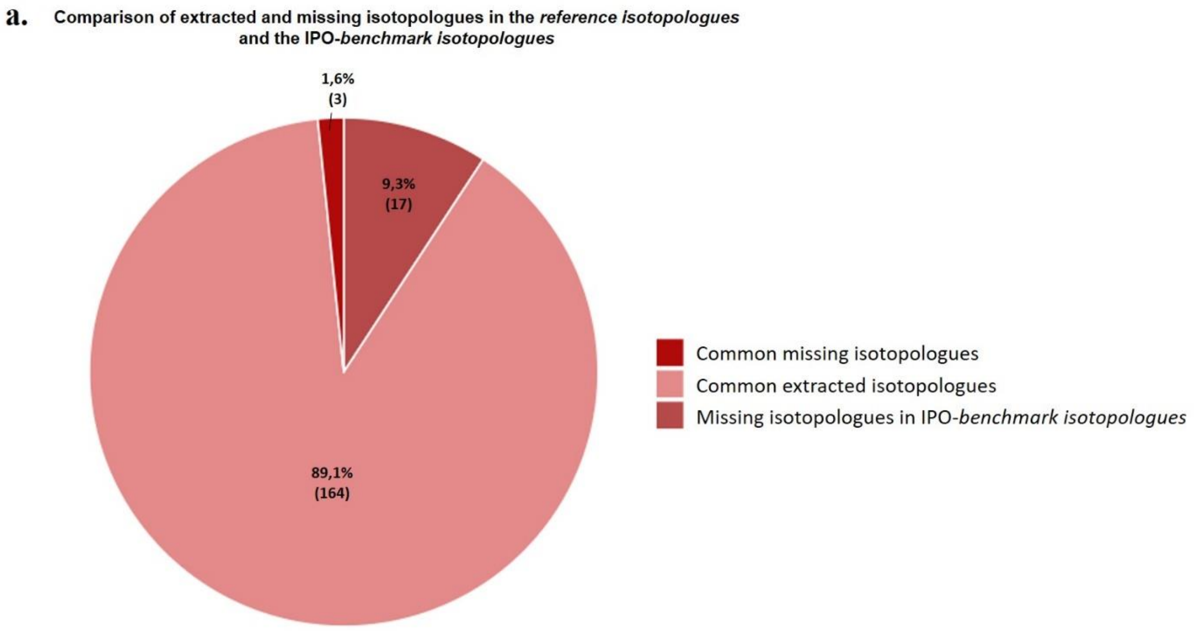

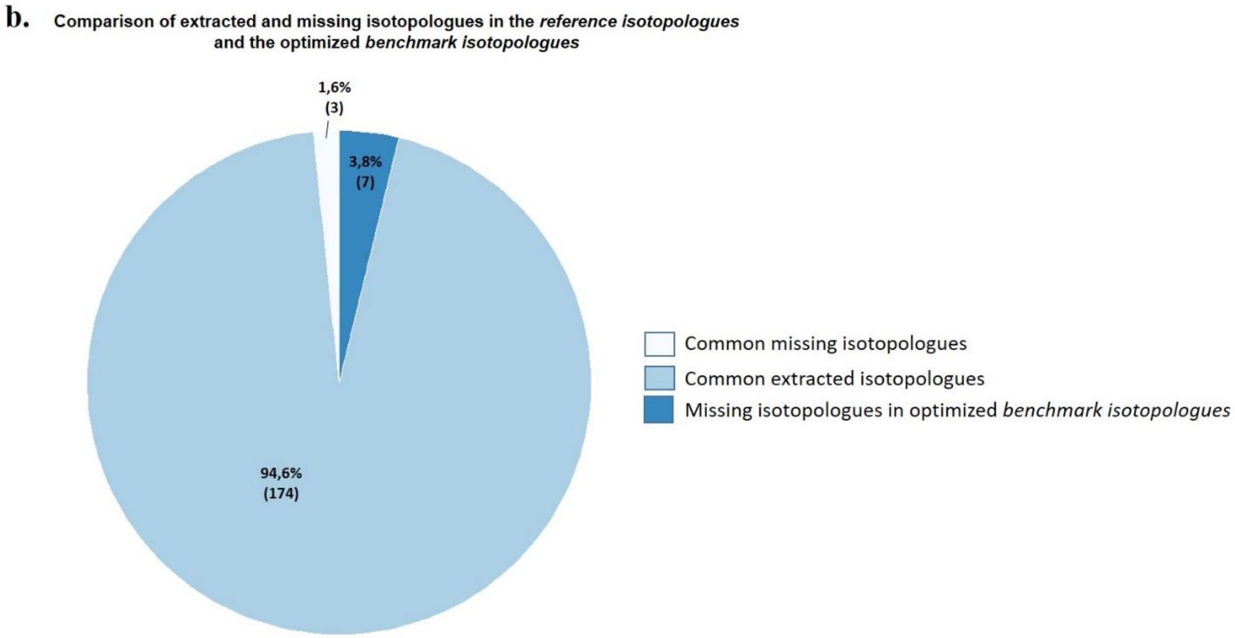

Table S-5. Mass accuracies of XCMS datasets. Mass accuracies for the 25 *reference metabolites* extracted in the five-labelled PT samples using XCMS compared to the theoretical masses.

				Starting parameter settings		Optimized parameter settings		
**Metabolite**	**Isotopologue**	**m/z theo**	**RT theo (sec)**	**m/z**	**Δm/z (ppm)**	**m/z**	**Δm/z (ppm)**	**RT (sec)**
**Fumarate**	M0	115.00368	981	115.00345	1.98	115.00345	1.98	980
	M1	116.00704	981	116.00682	1.86	116.00682	1.86	980
	M2	117.01039	981	117.01015	2.08	117.01019	1.69	979
	M3	118.01375	981	118.01356	1.57	118.01356	1.57	979
	M4	119.01710	981	119.01686	1.99	119.01687	1.94	979
**Succinate**	M0	117.01933	667	117.01911	1.90	117.01911	1.90	668
	M1	118.02269	667	118.02248	1.72	118.02248	1.72	668
	M2	119.02604	667	119.02571	2.75	119.02575	2.41	668
	M3	120.02940	667	120.02914	2.09	120.02913	2.18	668
	M4	121.03275	667	121.03254	1.77	121.03254	1.76	668
**Malate**	M0	133.01425	670	133.01408	1.24	133.01409	1.24	677
	M1	134.01761	670	134.01748	0.93	134.01748	0.93	677
	M2	135.02096	670	135.01841	18.87	135.02082	1.06	677
	M3	136.02432	670	136.02412	1.45	136.02413	1.37	677
	M4	137.02767	670	137.02750	1.25	137.02750	1.24	676
**Orotate**	M0	155.00983	1542	155.00978	0.31	155.00978	0.31	1 526
	M1	156.01319	1542	156.01312	0.39	156.01312	0.40	1 526
	M2	157.01654	1542	157.01631	1.48	157.01637	1.09	1 525
	M3	158.01990	1542	158.01968	1.35	158.01968	1.33	1 525
	M4	159.02325	1542	159.02309	1.03	159.02309	1.01	1 525
	M5	160.02661	1542	160.02655	0.36	160.02655	0.36	1 524
**a-KG**	M0	145.01425	882	145.01418	0.47	145.01418	0.48	884
	M1	146.01761	882	146.01756	0.32	146.01756	0.31	884
	M2	147.02096	882	147.02091	0.33	147.02091	0.33	883
	M3	148.02432	882	148.02428	0.21	148.02429	0.16	883
	M4	149.02767	882	149.02756	0.71	149.02756	0.71	883
	M5	150.03103	882	150.03097	0.35	150.03097	0.38	883
**Citrate**	M0	191.01973	1813	191.01987	0.75	191.01987	0.75	1 816
	M1	192.02309	1813	192.02324	0.79	192.02324	0.78	1 816
	M2	193.02644	1813	193.02399	12.70	193.02651	0.39	1 816
	M3	194.02980	1813	194.02984	0.25	194.02985	0.26	1 816
	M4	195.03315	1813	195.03317	0.12	195.03318	0.14	1 816
	M5	196.03651	1813	196.03662	0.57	196.03663	0.65	1 816
	M6	197.03986	1813	197.04026	2.03	197.04027	2.09	1 816
**2/3-PG**	M0	184.98566	1741	184.98571	0.25	184.98571	0.25	1 742
	M1	185.98902	1741	185.98908	0.36	185.98908	0.37	1 742
	M2	186.99237	1741	186.99010	12.13	186.99269	1.70	1 741
	M3	187.99573	1741	187.99577	0.23	187.99577	0.24	1 741
**PEP**	M0	166.97510	1889	166.97509	0.04	166.97509	0.04	1 890
	M1	167.97846	1889	167.97847	0.11	167.97847	0.11	1 890
	M2	168.98181	1889	168.98178	0.17	168.98190	0.54	1 890
	M3	169.98517	1889	169.98525	0.51	169.98524	0.45	1 890
**Gly-3P**	M0	171.0064	514	171.00642	0.10	171.00642	0.14	516
	M1	172.00976	514	172.00985	0.54	172.00985	0.54	516
	M2	173.01311	514	173.01318	0.38	173.01326	0.88	516
	M3	174.01647	514	174.01653	0.37	174.01655	0.47	516
**PRPP**	M0	388.94454	2154	388.94404	1.29	388.94404	1.28	2 162
	M1	389.94790	2154	389.94740	1.27	389.94740	1.27	2 162
	M2	390.95125	2154	390.95069	1.42	390.95067	1.48	2 160
	M3	391.95461	2154	-	-	391.95395	1.66	2 161
	M4	392.95796	2154	392.95726	1.78	392.95728	1.72	2 159
	M5	393.96132	2154	-	-	393.96043	2.24	2 158
**P5P**	M0	229.01188	1087	229.01215	1.17	229.01215	1.18	1 090

	M1	230.01524	1087	230.01552	1.25	230.01552	1.22	1 089
	M2	231.01859	1087	231.01755	4.50	231.01889	1.29	1 087
	M3	232.02195	1087	232.02228	1.43	232.02227	1.42	1 088
	M4	233.02530	1087	233.02564	1.45	233.02564	1.45	1 087
	M5	234.02866	1087	234.02907	1.79	234.02907	1.79	1 086
**FBP**	M0	338.98877	2082	338.98787	2.66	338.98784	2.73	2 087
	M1	339.99213	2082	339.99120	2.73	339.99120	2.72	2 087
	M2	340.99548	2082	340.99223	9.53	340.99454	2.76	2 087
	M3	341.99884	2082	341.99669	6.28	341.99769	3.34	2 087
	M4	343.00219	2082	343.00114	3.07	343.00114	3.05	2 087
	M5	344.00555	2082	344.00438	3.39	344.00436	3.43	2 087
	M6	345.00890	2082	345.00784	3.08	345.00782	3.12	2 087
**Sed7P**	M0	289.03301	1194	289.03281	0.68	289.03282	0.67	1 191
	M1	290.03637	1194	290.03619	0.59	290.03618	0.64	1 191
	M2	291.03972	1194	291.03719	8.69	291.03956	0.54	1 190
	M3	292.04308	1194	292.04291	0.56	292.04291	0.56	1 189
	M4	293.04643	1194	293.04627	0.53	293.04627	0.55	1 189
	M5	294.04979	1194	294.04965	0.45	294.04965	0.45	1 189
	M6	295.05314	1194	295.05189	4.22	295.05190	4.21	1 188
	M7	296.05650	1194	296.05520	4.37	296.05513	4.60	1 187
**Man6P**	M0	259.02244	1022	259.02288	1.70	259.02287	1.64	1 023
	M1	260.02580	1022	260.02629	1.90	260.02629	1.90	1 022
	M2	261.02915	1022	261.02963	1.82	261.02968	2.03	1 022
	M3	262.03251	1022	262.03302	1.98	262.03303	2.01	1 022
	M4	263.03586	1022	263.03640	2.04	263.03640	2.04	1 022
	M5	264.03922	1022	264.03962	1.52	264.03965	1.64	1 021
	M6	265.04257	1022	265.04312	2.08	265.04312	2.08	1 020
**F6P**	M0	259.02244	953	259.02283	1.49	259.02283	1.51	957
	M1	260.02580	953	260.02628	1.88	260.02629	1.89	956
	M2	261.02915	953	261.02954	1.50	261.02969	2.07	955
	M3	262.03251	953	262.03294	1.67	262.03285	1.30	952
	M4	263.03586	953	263.03642	2.14	263.03637	1.92	953
	M5	264.03922	953	264.03965	1.65	264.03966	1.67	953
	M6	265.04257	953	265.04310	2.01	265.04311	2.02	954
**G6P**	M0	259.02244	892	259.02281	1.41	259.02281	1.41	890
	M1	260.02580	892	260.02622	1.62	260.02624	1.72	889
	M2	261.02915	892	261.02832	3.16	261.02965	1.92	888
	M3	262.03251	892	262.03283	1.23	262.03283	1.22	888
	M4	263.03586	892	263.03640	2.05	263.03640	2.06	888
	M5	264.03922	892	264.03978	2.13	264.03977	2.10	887
	M6	265.04257	892	265.04305	1.83	265.04306	1.84	887
**G1P**	M0	259.02244	521	259.02295	1.96	259.02293	1.88	523
	M1	260.02579	521	260.02631	2.02	260.02631	2.01	523
	M2	261.02914	521	261.02974	2.31	261.02979	2.49	523
	M3	262.03249	521	-	-	262.03309	2.28	521
	M4	263.03584	521	-	-	263.03653	2.64	521
	M5	264.03919	521	264.03998	2.98	264.03996	2.90	523
	M6	265.04254	521	-	-	-	-	
**ADP**	M0	426.02214	2046	-	-	-	-	2 051
	M1	427.02550	2046	427.02544	0.12	427.02544	0.12	2 051
	M2	428.02885	2046	428.02845	0.94	428.02864	0.48	2 051
	M3	429.03221	2046	429.03199	0.49	429.03199	0.49	2 051
	M4	430.03556	2046	430.03533	0.54	430.03533	0.54	2 051
	M5	431.03892	2046	431.03867	0.56	431.03868	0.56	2 051
	M6	432.04227	2046	432.04203	0.56	432.04203	0.56	2 051
	M7	433.04563	2046	433.04547	0.36	433.04547	0.36	2 051
	M8	434.04898	2046	-	-	434.04849	1.14	2 051
	M9	435.05234	2046	-	-	435.05183	1.17	2 051
	M10	436.05569	2046	-	-	-	-	
**ATP**	M0	505.98847	2184	505.98900	1.06	505.98900	1.05	2 187

	M1	506.99183	2184	506.99234	1.02	506.99234	1.02	2 187
	M2	507.99518	2184	507.99487	0.62	507.99573	1.08	2 187
	M3	508.99854	2184	508.99885	0.61	508.99900	0.92	2 187
	M4	510.00189	2184	510.00232	0.84	510.00234	0.88	2 187
	M5	511.00525	2184	511.00559	0.68	511.00564	0.78	2 187
	M6	512.00860	2184	512.00900	0.79	512.00906	0.89	2 187
	M7	513.01196	2184	513.01234	0.74	513.01225	0.58	2 187
	M8	514.01531	2184	514.01565	0.65	514.01563	0.63	2 187
	M9	515.01867	2184	515.01883	0.32	515.01883	0.32	2 187
	M10	516.02202	2184	-	-	516.02078	2.41	2 185
**CDP**	M0	402.01090	1818	-	-	-	-	
	M1	403.01426	1818	403.01388	0.92	-	-	
	M2	404.01761	1818	404.01722	0.97	404.01727	0.84	1 818
	M3	405.02097	1818	405.02057	0.97	405.02060	0.91	1 818
	M4	406.02432	1818	406.02389	1.07	406.02388	1.07	1 818
	M5	407.02768	1818	407.02723	1.10	407.02723	1.08	1 818
	M6	408.03103	1818	408.03056	1.14	408.03059	1.08	1 818
	M7	409.03439	1818	-	-	409.03367	1.75	1 817
	M8	410.03774	1818	-	-	-	-	
	M9	411.04110	1818	-	-	-	-	
**CTP**	M0	481.97723	2106	481.97750	0.56	481.97752	0.60	2 116
	M1	482.98059	2106	482.98099	0.83	482.98099	0.84	2 117
	M2	483.98394	2106	483.98266	2.65	483.98428	0.69	2 116
	M3	484.98730	2106	484.98749	0.41	484.98751	0.44	2 116
	M4	485.99065	2106	485.99090	0.51	485.99088	0.47	2 116
	M5	486.99401	2106	486.99424	0.48	486.99422	0.44	2 116
	M6	487.99736	2106	487.99754	0.37	487.99754	0.37	2 116
	M7	489.00072	2106	489.00088	0.34	489.00089	0.35	2 116
	M8	490.00407	2106	490.00414	0.14	490.00412	0.11	2 116
	M9	491.00743	2106	-	-	491.00621	2.47	2 111
**GDP**	M0	442.01705	2258	442.01707	0.03	-	-	
	M1	443.02040	2258	443.02067	0.61	443.02067	0.61	2 266
	M2	444.02375	2258	444.02356	0.42	444.02373	0.04	2 266
	M3	445.02710	2258	445.02709	0.02	445.02708	0.03	2 266
	M4	446.03045	2258	446.03040	0.11	446.03039	0.14	2 266
	M5	447.03380	2258	447.03374	0.14	447.03374	0.14	2 266
	M6	448.03715	2258	448.03708	0.15	448.03708	0.15	2 266
	M7	449.04050	2258	449.04039	0.25	449.04039	0.26	2 262
	M8	450.04385	2258	-	-	450.04372	0.28	2 260
	M9	451.04720	2258	-	-	451.04674	1.02	2 259
	M10	452.05055	2258	-	-	-	-	
**UDP**	M0	402.99492	2202	402.99449	1.07	-	-	
	M1	403.99826	2202	403.99806	0.50	403.99805	0.51	2 208
	M2	405.00160	2202	405.00110	1.23	405.00128	0.78	2 208
	M3	406.00494	2202	406.00457	0.91	406.00457	0.90	2 208
	M4	407.00828	2202	407.00797	0.76	407.00797	0.75	2 207
	M5	408.01162	2202	408.01130	0.77	408.01128	0.84	2 208
	M6	409.01496	2202	409.01466	0.73	409.01466	0.73	2 208
	M7	410.01830	2202	410.01766	1.55	410.01754	1.85	2 203
	M8	411.02164	2202	411.02089	1.81	411.02095	1.68	2 205
	M9	412.02498	2202	-	-	412.02337	3.92	2 203
**UMP**	M0	323.02859	1931	323.02754	3.25	323.02753	3.29	1 936
	M1	324.03195	1931	324.03099	2.94	324.03099	2.95	1 936
	M2	325.03530	1931	325.03311	6.73	325.03430	3.08	1 936
	M3	326.03866	1931	326.03607	7.93	326.03760	3.25	1 935
	M4	327.04201	1931	327.04089	3.41	327.04090	3.40	1 935
	M5	328.04537	1931	328.04421	3.53	328.04421	3.53	1 935
	M6	329.04872	1931	329.04755	3.56	329.04755	3.55	1 935
	M7	330.05208	1931	330.05090	3.57	330.05090	3.56	1 935
	M8	331.05543	1931	331.05424	3.60	331.05424	3.58	1 935

	M9	332.05879	1931	332.05715	4.91	332.05708	5.13	1 934
**UTP**	M0	482.96125	2208	482.96167	0.86	482.96167	0.87	2 209
	M1	483.96460	2208	483.96510	1.03	483.96510	1.04	2 210
	M2	484.96795	2208	484.96781	0.29	484.96837	0.86	2 210
	M3	485.97130	2208	485.97146	0.34	485.97165	0.73	2 209
	M4	486.97465	2208	486.97495	0.61	486.97497	0.65	2 209
	M5	487.97800	2208	487.97833	0.68	487.97834	0.70	2 209
	M6	488.98135	2208	488.98168	0.68	488.98169	0.69	2 209
	M7	489.98470	2208	489.98491	0.43	489.98492	0.44	2 209
	M8	490.98805	2208	490.98793	0.25	490.98792	0.27	2 210
	M9	491.99140	2208	-	-	491.99023	2.38	2 203

Figure S-4. Impact of parameter optimization on the measurement of isotopologue abundances. Comparison of the integrated areas of benchmark isotopologues (red bars) and reference isotopologues (black line) in a log scale.

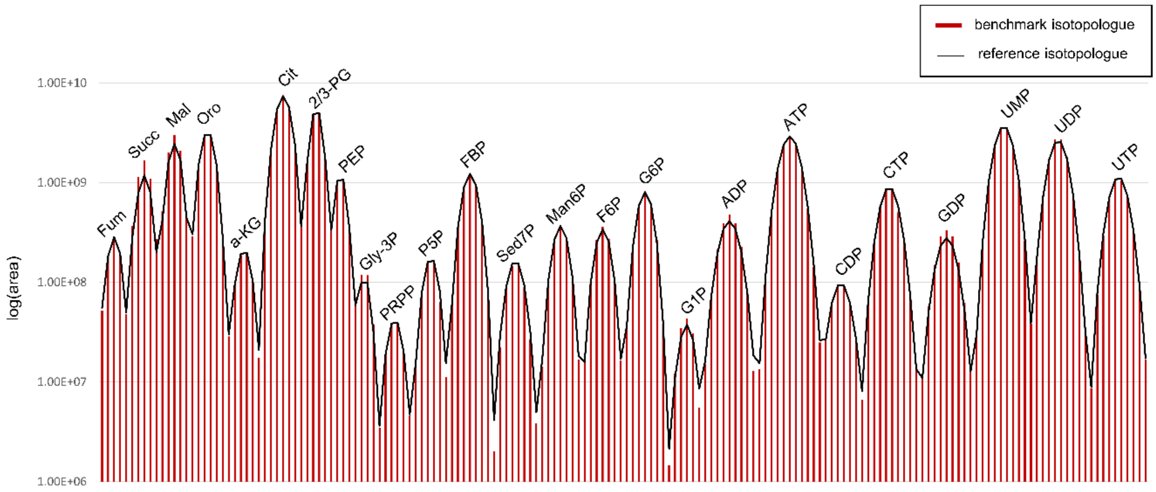

Figure S-5. CIDs comparison between optimized and non-optimized datasets. CIDs comparison between theoretical distribution (yellow), *reference CIDs* (dark blue), IPO *benchmark CIDs* (light red) and optimized *benchmark CIDs* (light blue) for the 25-*reference metabolites*.

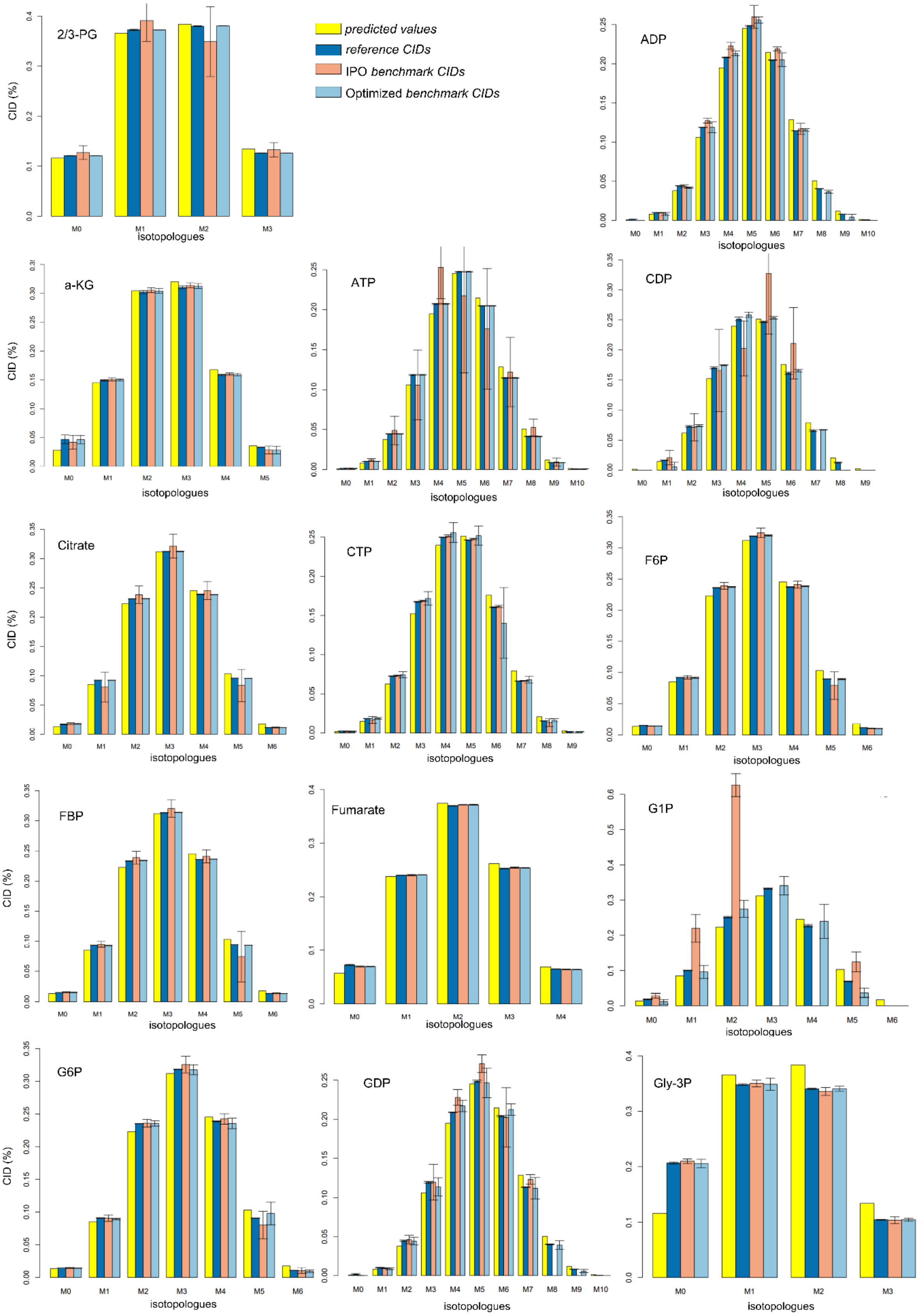

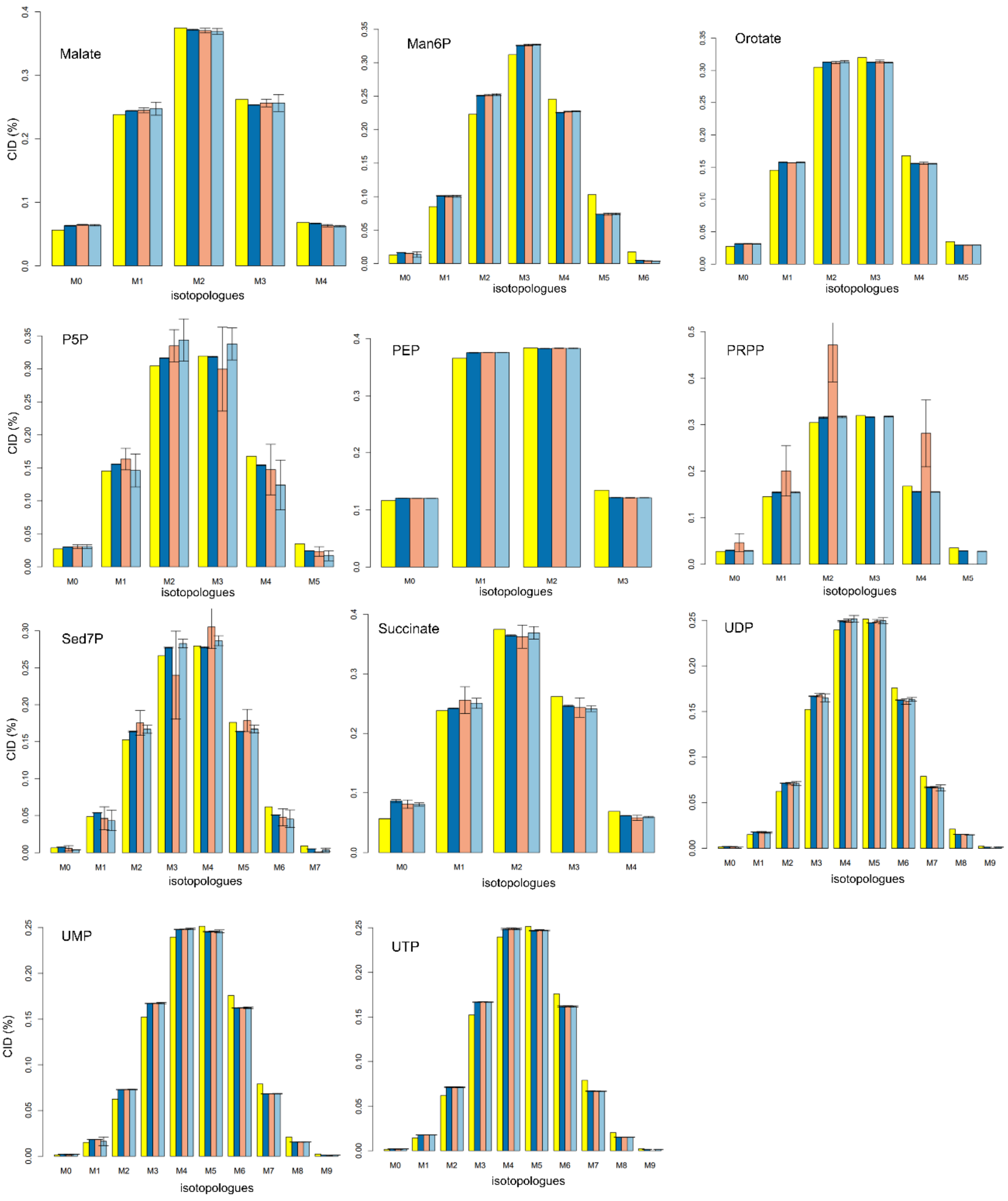
Malate
Man6P
Orotate
P5P
PEP
PRPP
Sed7P
Succinate
UDP
UMP
UTP
CID (%)
isotopologues
M0
M1
M2
M3
M4
M5
M6
M7
M8
M9

Figure S-6. Clustering software redundancies after manual curation. Comparison of the number of isotopic clusters each detected isotopologue appears in for X13CMS (blue) and geoRge (red) software after manual curation of obvious redundancies.

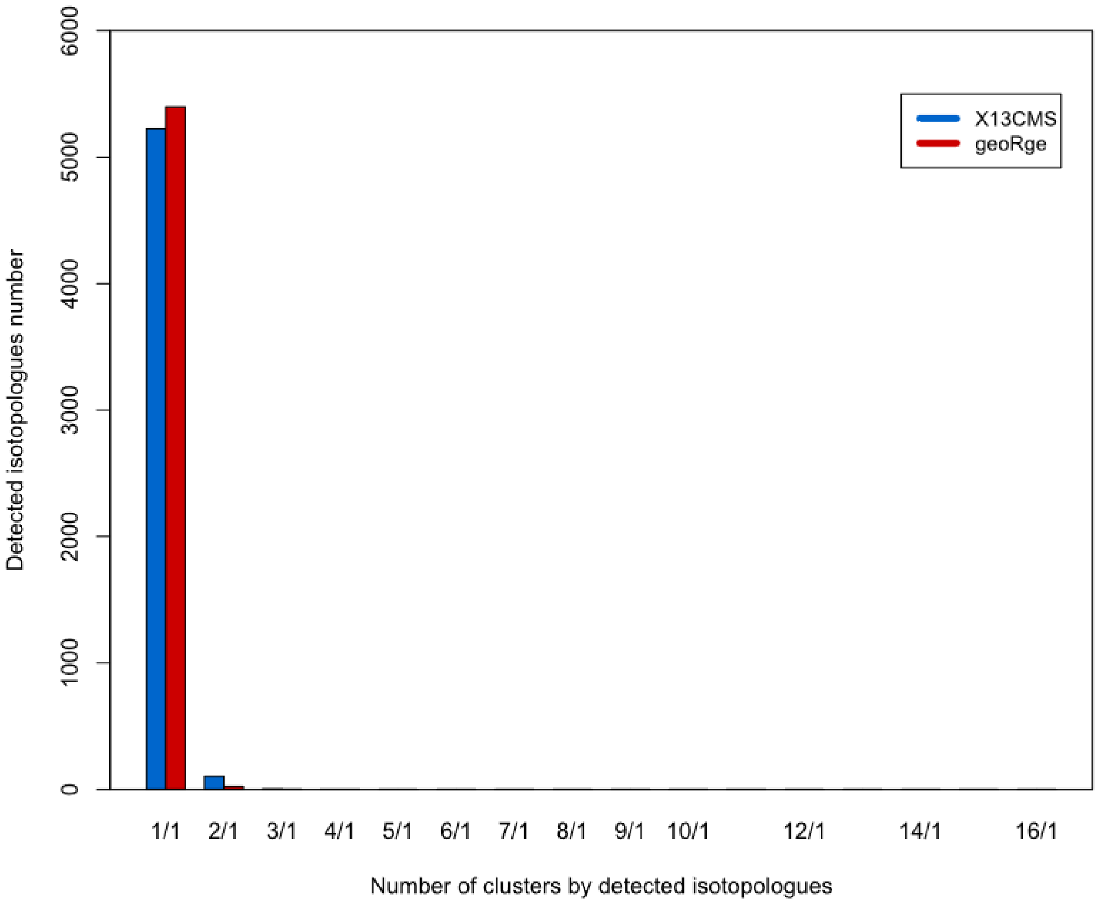

Table S-6. Cluster precision and recall for clustering software. Cluster precision and recall for X13CMS and geoRge with a RT window of 5 s and different isotopologue mass deviations, evaluated for the 25 reference metabolites in the PT sample.

	Isotopologue mass deviation (ppm)	1	2	3	5	8	10
**X13CMS**	precision	60%	76%	76%	84%	80%	80%
	recall	100%	100%	100%	100%	100%	100%
**geoRge**	precision	60%	68%	72%	80%	74%	72%
	recall	100%	100%	100%	100%	100%	100%

Figure S-7. Carbon mass fractions for significant metabolites between two *E.coli* strains. Carbon mass fractions comparison between the wild-type (green) and the Δzwf strains (red) for significant metabolites identified with a level 1 confidence.

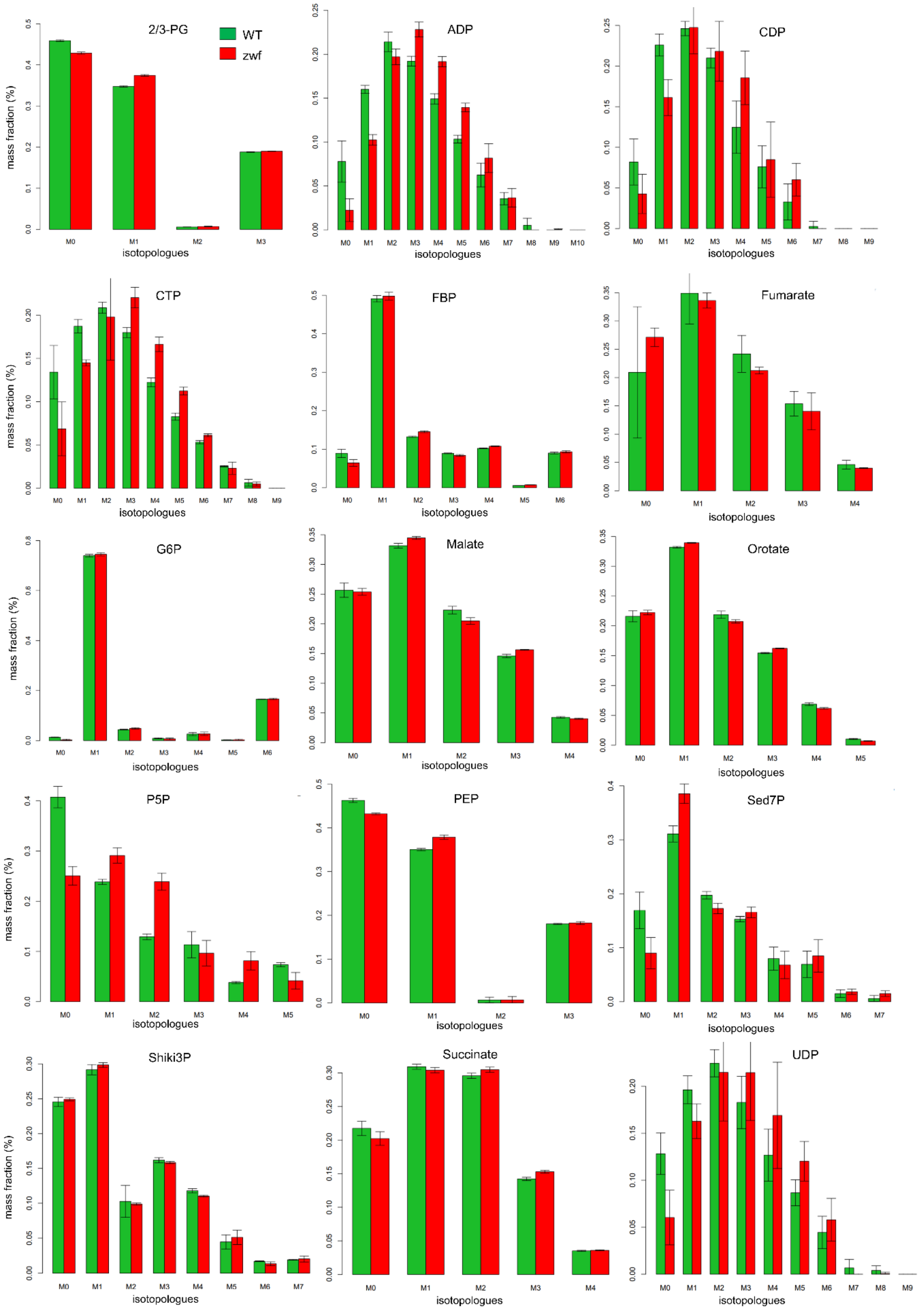

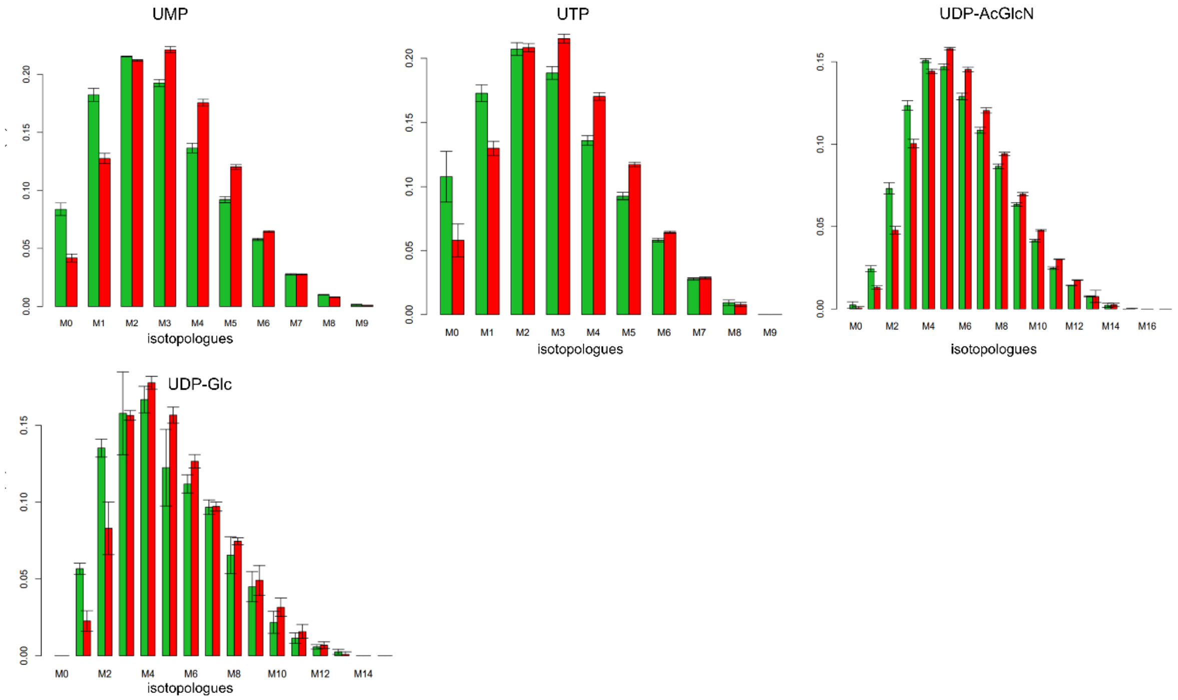
UMP
0.00
0.05
0.10
0.15
0.20
M0 M1 M2 M3 M4 M5 M6 M7 M8 M9
isotopologues
UTP
0.00
0.05
0.10
0.15
0.20
M0 M1 M2 M3 M4 M5 M6 M7 M8 M9
isotopologues
UDP-AcGlcN
0.00
0.05
0.10
0.15
M0 M2 M4 M6 M8 M10 M12 M14 M16
isotopologues
UDP-Glc
0.00
0.05
0.10
0.15
M0 M2 M4 M6 M8 M10 M12 M14
isotopologues

Table S-7. Overlapping isotopic clusters. The m/z and RT pairs of the 797 overlapping isotopic clusters between X13CMS and geoRge software.

Cluster_id	isotopologues	m/z	rt
**C12**	M0	82.02974	532
	M2	84.03646	531
**C23**	M0	85.02946	1987
	M1	86.03283	1987
	M2	87.03618	1987
	M3	88.03952	1987
	M4	89.04294	1987
**C24**	M0	85.02946	1897
	M1	86.03283	1897
	M2	87.03618	1897
	M3	88.03950	1898
**C26**	M0	85.02945	646
	M1	86.03283	646
	M2	87.03618	646
**C27**	M0	85.06584	802
	M1	86.06921	800
	M2	87.07256	800
	M3	88.07594	800
	M4	89.07931	799
**C35**	M0	87.00874	239
	M1	88.01211	239
	M2	89.01548	240
	M3	90.01885	239
**C37**	M0	87.00873	1816
	M1	88.01210	1817
	M2	89.01544	1816
	M3	90.01879	1816
**C36**	M0	87.00875	194
	M1	88.01212	190
	M3	90.01885	195
**C53**	M0	88.04038	633
	M1	89.04375	633
	M2	90.04711	633
	M3	91.05048	633
**C54**	M0	88.04039	557
	M1	89.04376	556
	M2	90.04711	556
	M3	91.05048	557
**C55**	M0	88.04036	771
	M1	89.04376	773
	M2	90.04711	772
**C57**	M0	88.98803	958
	M1	89.99137	958

	M2	90.99476	959
**C62**	M0	89.02441	181
	M1	90.02775	182
	M2	91.03115	184
	M3	92.03454	190
**C64**	M0	89.02443	254
	M1	90.02778	252
	M2	91.03113	244
	M3	92.03455	252
**C96**	M0	91.05535	2184
	M1	92.05872	2183
	M2	93.06208	2183
	M3	94.06546	2183
	M4	95.06883	2183
	M5	96.07220	2182
	M6	97.07553	2182
**C105**	M0	93.03465	260
	M1	94.03801	260
	M2	95.04140	260
	M3	96.04478	260
	M4	97.04811	260
	M5	98.05149	260
**C104**	M0	93.03464	1753
	M1	94.03805	1753
	M2	95.04141	1753
	M3	96.04477	1753
	M4	97.04807	1753
	M5	98.05147	1753
	M6	99.05485	1753
**C107**	M0	93.03462	2131
	M1	94.03801	2130
	M2	95.04139	2130
**C124**	M0	94.98091	1024
	M1	95.98430	1024
**C122**	M0	94.98093	1796
	M1	95.98436	1795
**C126**	M0	95.01396	204
	M1	96.01735	203
**C131**	M0	95.05033	2131
	M1	96.05369	2132
	M2	97.05702	2131
	M3	98.06039	2131
	M4	99.06379	2131
**C202**	M0	98.02487	532
	M1	99.02825	531
	M2	100.03162	531

	M3	101.03478	531
**C203**	M0	98.02487	1871
	M1	99.02822	1871
	M2	100.03163	1871
	M3	101.03478	1870
**C216**	M0	99.00891	668
	M1	100.01229	668
	M2	101.01548	668
	M3	102.01884	668
	M4	103.02221	670
**C218**	M0	99.00891	1831
	M1	100.01227	1831
	M2	101.01545	1831
**C217**	M0	99.00892	176
	M1	100.01227	175
**C237**	M0	100.07695	541
	M1	101.08013	540
	M2	102.08349	540
	M3	103.08687	540
**C241**	M0	101.02438	884
	M1	102.02772	884
	M2	103.03111	883
	M3	104.03446	883
	M4	105.03765	883
**C243**	M0	101.02441	647
	M1	102.02780	647
	M2	103.03117	647
	M3	104.03454	647
**C242**	M0	101.02442	202
	M1	102.02779	202
	M4	105.03771	204
**C259**	M0	102.05606	540
	M1	103.05944	540
	M2	104.06281	540
	M3	105.06598	540
**C263**	M0	102.05606	816
	M1	103.05943	815
	M2	104.06281	813
	M3	105.06599	813
	M4	106.06935	813
**C266**	M0	103.00371	732
	M1	104.00709	732
	M2	105.01027	729
	M3	106.01364	729
**C272**	M0	103.04010	189
	M1	104.04346	195

	M2	105.04666	194
	M3	106.05003	195
**C273**	M0	103.04010	646
	M1	104.04348	646
	M2	105.04666	646
	M3	106.05002	646
	M4	107.05340	647
**C260**	M0	102.05607	985
	M1	103.05944	984
	M2	104.06281	985
**C298**	M0	105.01920	1741
	M1	106.02257	1741
	M2	107.02591	1741
	M3	108.02932	1741
**C299**	M0	105.01920	181
	M1	106.02258	180
	M2	107.02592	178
	M3	108.02932	179
**C322**	M0	107.05015	2217
	M1	108.05352	2217
	M2	109.05669	2217
	M3	110.06006	2217
	M4	111.06343	2217
	M5	112.06680	2217
	M6	113.07017	2217
	M7	114.07349	2216
**C324**	M0	108.02159	2321
	M2	110.02817	2320
	M3	111.03171	2324
**C332**	M0	109.02924	2321
	M1	110.03261	2321
	M2	111.03599	2321
	M3	112.03935	2321
	M4	113.04272	2321
	M5	114.04603	2321
	M6	115.04939	2321
**C346**	M0	110.97552	443
	M1	111.97886	443
**C351**	M0	111.00854	1816
	M1	112.01190	1816
	M2	113.01529	1816
	M3	114.01859	1816
	M4	115.02196	1816
	M5	116.02532	1816
**C352**	M0	111.00854	1899
	M1	112.01192	1899

	M2	113.01529	1899
	M3	114.01858	1899
**C356**	M0	111.01975	1526
	M1	112.02309	1526
	M2	113.02641	1525
	M3	114.02973	1525
	M4	115.03312	1525
**C376**	M0	112.04019	557
	M1	113.04356	557
	M2	114.04686	557
	M3	115.05022	557
	M4	116.05360	557
**C387**	M0	112.98538	594
	M1	113.98862	594
**C389**	M0	112.99905	1484
	M1	114.00238	1484
**C393**	M0	113.02424	204
	M1	114.02757	204
	M5	118.04110	202
**C400**	M0	113.03545	676
	M1	114.03876	676
	M2	115.04214	676
	M3	116.04551	676
	M4	117.04887	675
**C406**	M0	113.06060	801
	M1	114.06392	799
	M2	115.06728	799
	M3	116.07066	799
	M4	117.07402	799
**C412**	M0	114.01943	557
	M1	115.02279	557
	M2	116.02617	556
	M3	117.02953	556
	M4	118.03286	555
**C413**	M0	114.01942	633
	M1	115.02279	633
	M2	116.02616	633
	M3	117.02952	633
	M4	118.03290	633
**C414**	M0	114.01944	678
	M1	115.02283	677
	M2	116.02620	677
**C371**	M0	112.01682	1525
	M3	115.02686	1531
**C435**	M0	115.00345	980
	M1	116.00682	980

	M2	117.01019	979
	M3	118.01356	979
	M4	119.01687	979
**C432**	M0	115.00346	633
	M1	116.00683	633
	M2	117.01020	632
	M3	118.01359	632
	M4	119.01691	633
**C434**	M0	115.00346	678
	M1	116.00685	678
	M2	117.01024	678
	M3	118.01368	678
	M4	119.01710	677
**C436**	M0	115.00345	557
	M1	116.00683	556
	M2	117.01020	555
	M3	118.01354	555
**C433**	M0	115.00346	803
	M1	116.00682	803
	M2	117.01019	803
	M3	118.01357	802
**C437**	M0	115.01468	1484
	M1	116.01805	1485
	M2	117.02134	1487
**C447**	M0	115.03984	802
	M1	116.04320	802
	M2	117.04658	800
	M3	118.04995	800
	M4	119.05325	800
	M5	120.05662	800
**C449**	M0	115.03984	338
	M1	116.04322	332
**C473**	M0	116.03509	171
	M1	117.03846	170
**C494**	M0	117.01911	668
	M1	118.02248	668
	M2	119.02575	668
	M3	120.02913	668
	M4	121.03254	668
**C505**	M0	117.05555	231
	M1	118.05892	232
	M2	119.06225	233
	M3	120.06562	233
	M4	121.06899	233
	M5	122.07236	232
**C523**	M0	118.05079	531

	M1	119.05410	531
	M2	120.05748	531
	M3	121.06085	531
	M4	122.06421	531
**C536**	M0	119.03478	175
	M1	120.03814	175
	M2	121.04148	174
	M3	122.04487	175
	M4	123.04827	174
**C555**	M0	121.02933	2229
	M1	122.03271	2230
	M2	123.03609	2230
	M3	124.03940	2228
	M4	125.04277	2230
	M5	126.04614	2228
**C557**	M0	121.02932	2131
	M1	122.03270	2131
	M2	123.03607	2130
	M3	124.03937	2131
**C533**	M0	119.02328	668
	M3	122.03346	670
**C565**	M0	122.02461	968
	M1	123.02798	968
	M2	124.03127	967
	M3	125.03465	967
	M4	126.03802	967
**C564**	M0	122.02461	1731
	M1	123.02798	1730
	M2	124.03124	1730
**C607**	M0	125.02422	846
	M1	126.02754	845
**C608**	M0	125.02423	206
	M1	126.02762	206
**C610**	M0	125.03464	1829
	M1	126.03785	1829
	M2	127.04132	1828
	M3	128.04471	1828
**C632**	M0	126.95279	823
	M1	127.95613	821
**C645**	M0	128.03520	540
	M1	129.03857	540
	M2	130.04188	540
	M3	131.04526	540
	M4	132.04863	540
**C646**	M0	128.03519	815
	M1	129.03858	813

	M2	130.04188	812
	M4	132.04863	812
**C644**	M0	128.03522	176
	M1	129.03856	175
	M2	130.04185	177
	M3	131.04521	179
	M4	132.04860	179
**C660**	M0	129.01923	1987
	M1	130.02254	1987
	M2	131.02591	1987
	M3	132.02934	1987
	M4	133.03265	1987
**C658**	M0	129.01923	1897
	M1	130.02254	1897
	M2	131.02592	1897
	M3	132.02954	1897
	M4	133.03273	1898
**C659**	M0	129.01923	646
	M1	130.02257	646
	M2	131.02594	646
**C661**	M0	129.01923	1816
	M1	130.02255	1815
	M2	131.02590	1814
	M3	132.02923	1813
	M4	133.03256	1813
**C657**	M0	129.01924	764
	M1	130.02257	762
**C650**	M0	128.03521	985
	M1	129.03858	985
	M2	130.04189	985
**C701**	M0	130.05080	557
	M1	131.05418	557
	M2	132.05755	557
	M3	133.06091	557
	M4	134.06428	557
	M5	135.06763	557
**C715**	M0	131.03490	203
	M1	132.03824	203
**C724**	M0	131.07125	280
	M1	132.07463	280
	M2	133.07799	279
	M3	134.08136	279
	M4	135.08471	279
	M5	136.08801	279
**C723**	M0	131.07120	802
	M1	132.07460	802

	M2	133.07796	800
	M3	134.08133	800
	M4	135.08470	800
	M5	136.08801	799
**C731**	M0	132.03004	633
	M1	133.03342	633
	M2	134.03678	633
	M3	135.04014	633
	M4	136.04346	633
**C761**	M0	132.99640	1831
	M1	133.99977	1831
	M2	135.00310	1831
	M3	136.00643	1832
**C762**	M0	133.01409	677
	M1	134.01748	677
	M2	135.02082	677
	M3	136.02413	677
	M4	137.02750	676
**C777**	M0	133.05047	189
	M1	134.05385	189
	M2	135.05717	190
	M3	136.06047	190
	M4	137.06386	190
	M5	138.06724	190
**C763**	M0	133.01414	816
	M1	134.01755	813
	M2	135.02090	813
**C821**	M0	135.01206	903
	M1	136.01535	903
**C833**	M0	135.04509	2335
	M1	136.04838	2335
**C804**	M0	134.04717	2188
	M1	135.05047	2187
	M2	136.05376	2186
	M3	137.05715	2186
**C794**	M0	134.03432	633
	M3	137.04437	633
**C867**	M0	137.02431	260
	M1	138.02769	261
	M2	139.03110	261
	M3	140.03447	261
	M4	141.03784	261
	M5	142.04117	261
**C866**	M0	137.02429	1753
	M1	138.02768	1753
	M2	139.03105	1753

	M3	140.03443	1753
	M4	141.03781	1753
	M5	142.04112	1752
	M6	143.04448	1752
	M7	144.04786	1752
**C822**	M0	135.01834	677
	M3	138.02836	677
	M4	139.03180	677
**C836**	M0	135.05474	190
	M1	136.05811	191
	M4	139.06824	190
**C889**	M0	138.97057	1024
	M1	139.97395	1024
**C891**	M0	138.98018	2087
	M1	139.98347	2087
	M2	140.98678	2087
**C890**	M0	138.98011	890
	M1	139.98346	889
	M2	140.98629	896
**C918**	M0	140.03524	480
	M1	141.03863	480
	M6	146.05542	480
**C907**	M0	139.11273	485
	M2	141.11952	485
	M3	142.12282	484
	M4	143.12620	485
	M5	144.12956	485
**C954**	M0	142.98075	2288
	M1	143.98410	2290
	M2	144.98754	2290
	M3	145.99088	2290
	M4	146.99420	2291
**C957**	M0	143.03494	204
	M1	144.03830	205
**C989**	M0	144.06654	541
	M1	145.06991	540
	M2	146.07327	540
	M3	147.07665	540
	M4	148.08002	540
	M5	149.08333	540
**C995**	M0	144.98077	2127
	M1	145.98428	2125
**C998**	M0	145.01418	884
	M1	146.01756	884
	M2	147.02091	883
	M3	148.02429	883

	M4	149.02756	883
	M5	150.03097	883
**C1005**	M0	145.05060	272
	M1	146.05396	272
	M2	147.05737	272
	M3	148.06077	272
	M4	149.06408	272
	M5	150.06748	272
	M6	151.07084	272
**C1022**	M0	146.04583	171
	M1	147.04920	171
	M2	148.05255	171
	M3	149.05584	171
	M4	150.05924	171
	M5	151.06264	171
**C1025**	M0	146.04582	912
	M1	147.04920	911
	M2	148.05257	910
	M3	149.05585	911
	M4	150.05924	911
	M5	151.06264	911
**C1023**	M0	146.04582	815
	M1	147.04920	814
	M2	148.05256	813
	M3	149.05585	813
	M4	150.05923	814
	M5	151.06261	814
**C1044**	M0	147.02984	646
	M1	148.03321	646
	M2	149.03651	646
	M3	150.03987	646
	M4	151.04326	646
	M5	152.04666	646
**C1041**	M0	147.02985	1816
	M1	148.03327	1816
	M2	149.03654	1816
	M3	150.03992	1816
	M4	151.04315	1813
**C1060**	M0	147.06621	242
	M1	148.06957	242
	M2	149.07287	242
	M3	150.07620	243
	M4	151.07955	243
	M5	152.08295	243
	M6	153.08636	242
**C1089**	M0	148.96443	1890

	M1	149.96782	1890
	M2	150.97112	1890
	M3	151.97456	1890
**C1092**	M0	149.00908	734
	M1	150.01247	733
**C1095**	M0	149.02771	278
	M1	150.03110	278
	M2	151.03446	278
	M3	152.03788	278
	M4	153.04122	278
	M5	154.04460	278
**C1101**	M0	149.04546	177
	M1	150.04888	179
	M2	151.05219	177
	M3	152.05552	174
**C1144**	M0	150.97062	787
	M1	151.97401	784
**C1147**	M0	150.98031	980
	M1	151.98365	980
	M3	153.99037	979
**C1174**	M0	152.01876	1944
	M1	153.02221	1944
	M2	154.02555	1943
**C1182**	M0	152.98629	907
	M1	153.98967	908
**C1185**	M0	152.99582	516
	M1	153.99918	513
	M2	155.00245	513
**C1188**	M0	153.01929	2321
	M1	154.02267	2321
	M2	155.02606	2321
	M3	156.02940	2320
	M4	157.03266	2320
	M5	158.03603	2321
	M6	159.03943	2320
	M7	160.04282	2320
**C1190**	M0	153.01931	749
	M1	154.02268	747
	M2	155.02609	747
	M3	156.02945	747
	M4	157.03271	746
**C1117**	M0	149.07033	242
	M1	150.07359	242
	M5	154.08720	242
**C1202**	M0	153.98548	1978
	M1	154.98876	1978

**C1197**	M0	153.05565	1156
	M2	155.06246	1156
	M3	156.06575	1155
	M4	157.06911	1155
**C1218**	M0	154.97513	1779
	M1	155.97847	1778
	M2	156.98179	1778
**C1226**	M0	155.00978	1526
	M1	156.01312	1526
	M2	157.01637	1525
	M3	158.01968	1525
	M4	159.02309	1525
	M5	160.02655	1524
**C1230**	M0	155.03498	260
	M1	156.03835	260
	M2	157.04165	260
	M3	158.04503	260
	M4	159.04841	259
	M5	160.05179	260
	M6	161.05518	260
	M7	162.05853	259
**C1242**	M0	156.03026	557
	M1	157.03349	557
	M2	158.03682	557
	M3	159.04022	557
	M4	160.04356	557
	M5	161.04683	556
**C1246**	M0	156.06655	531
	M1	157.06992	531
	M2	158.07331	531
	M3	159.07667	531
	M4	160.08002	531
	M5	161.08341	531
**C1254**	M0	157.02538	676
	M1	158.02876	676
	M2	159.03212	676
	M3	160.03548	676
	M4	161.03888	675
	M5	162.04232	675
**C1255**	M0	157.02542	817
	M1	158.02879	815
	M2	159.03222	816
	M3	160.03554	817
**C1260**	M0	157.05055	926
	M1	158.05394	926
	M2	159.05731	926

	M3	160.06068	926
	M4	161.06406	926
	M5	162.06745	926
	M6	163.07084	925
	M7	164.07417	925
**C1270**	M0	158.00944	633
	M1	159.01281	633
	M2	160.01625	633
	M3	161.01966	633
	M4	162.02292	633
**C1284**	M0	158.08224	573
	M1	159.08562	573
	M2	160.08900	573
	M3	161.09238	574
	M4	162.09574	573
**C1301**	M0	159.00469	1485
	M1	160.00808	1484
	M2	161.01175	1484
	M3	162.01489	1484
**C1304**	M0	159.02986	176
	M1	160.03326	175
**C1279**	M0	158.04587	452
	M1	159.04926	453
	M2	160.05260	452
**C1250**	M0	157.01380	1526
	M4	161.02737	1524
**C1331**	M0	160.02510	602
	M1	161.02848	604
**C1344**	M0	160.06147	169
	M1	161.06483	169
	M2	162.06821	169
	M3	163.07168	168
	M4	164.07491	160
	M5	165.07825	161
**C1367**	M0	161.04554	202
	M1	162.04893	204
	M6	167.06572	203
**C1373**	M0	161.08194	342
	M1	162.08536	342
	M2	163.08871	341
	M3	164.09200	341
	M4	165.09538	341
	M5	166.09875	341
	M6	167.10211	341
**C1374**	M0	161.08196	266
	M1	162.08535	266

	M2	163.08871	266
	M3	164.09202	266
**C1395**	M0	162.04892	670
	M1	163.05226	670
	M2	164.05560	670
	M3	165.05894	670
**C1389**	M0	162.02305	728
	M2	164.02984	726
**C1420**	M0	163.04012	1238
	M1	164.04348	1238
	M2	165.04678	1238
	M3	166.05013	1238
	M4	167.05353	1238
	M5	168.05688	1237
**C1393**	M0	162.04080	575
	M3	165.05082	578
**C1462**	M0	165.05570	615
	M1	166.05913	614
	M2	167.06252	615
	M3	168.06587	614
	M4	169.06925	614
	M5	170.07260	614
	M6	171.07599	614
**C1483**	M0	166.96562	1796
	M1	167.96901	1796
	M3	169.97580	1795
**C1484**	M0	166.97509	1890
	M1	167.97847	1890
	M2	168.98190	1890
	M3	169.98524	1890
**C1485**	M0	166.97513	779
	M1	167.97852	776
	M2	168.98184	779
	M3	169.98527	776
**C1486**	M0	166.97512	2183
	M1	167.97846	2186
	M2	168.98179	2186
**C1510**	M0	167.99726	787
	M1	169.00064	787
	M2	170.00406	785
	M3	171.00737	785
**C1522**	M0	168.98130	896
	M1	169.98465	896
	M2	170.98806	896
	M3	171.99144	896
**C1527**	M0	168.99075	2087

	M1	169.99421	2087
	M2	170.99756	2087
	M3	172.00092	2087
**C1537**	M0	169.49249	2087
	M1	170.49585	2087
	M2	171.49924	2087
**C1542**	M0	169.99315	787
	M1	170.99658	785
	M3	173.00306	785
**C1566**	M0	171.00642	516
	M1	172.00985	516
	M2	173.01326	516
	M3	174.01655	516
**C1603**	M0	172.06164	192
	M1	173.06485	192
	M2	174.06834	191
	M3	175.07165	190
	M4	176.07499	190
	M5	177.07838	185
**C1615**	M0	173.00913	1897
	M1	174.01258	1897
	M2	175.01596	1897
	M3	176.01933	1897
	M4	177.02271	1897
	M5	178.02609	1897
	M6	179.02954	1897
**C1617**	M0	173.00911	1815
	M2	175.01595	1813
	M3	176.01933	1813
	M4	177.02270	1813
**C1569**	M0	171.01227	1845
	M1	172.01559	1845
	M4	175.02491	1837
**C1644**	M0	174.04073	557
	M1	175.04408	557
	M2	176.04744	557
	M3	177.05075	557
	M4	178.05407	557
	M5	179.05754	557
	M6	180.06107	557
**C1623**	M0	173.04563	713
	M1	174.04896	711
	M2	175.05230	711
	M3	176.05565	711
	M4	177.05902	711
**C1670**	M0	175.03599	633

	M1	176.03936	633
	M2	177.04268	633
	M3	178.04601	633
	M4	179.04942	633
	M5	180.05301	633
**C1671**	M0	175.03614	770
	M1	176.03952	768
**C1677**	M0	175.06115	802
	M1	176.06454	802
	M2	177.06790	800
	M3	178.07129	801
	M4	179.07457	800
	M5	180.07801	799
	M6	181.08146	799
	M7	182.08478	799
**C1691**	M0	176.01211	480
	M1	177.01550	480
**C1728**	M0	177.04055	176
	M1	178.04386	176
	M6	183.06080	176
**C1745**	M0	178.01811	273
	M1	179.02142	273
	M2	180.02482	272
	M3	181.02818	273
	M4	182.03154	273
**C1766**	M0	179.01985	711
	M1	180.02319	709
**C1780**	M0	179.05623	203
	M1	180.05959	204
	M6	185.07640	203
**C1778**	M0	179.05624	166
	M1	180.05957	166
	M6	185.07646	165
**C1779**	M0	179.05626	332
	M1	180.05963	331
**C1727**	M0	177.04015	633
	M1	178.04360	633
	M4	181.05363	633
**C1755**	M0	178.05102	2414
	M1	179.05438	2411
	M2	180.05776	2413
	M3	181.06112	2416
	M4	182.06453	2413
	M5	183.06786	2412
**C1699**	M0	176.04477	557
	M1	177.04824	557

	M5	181.06177	557
**C1807**	M0	181.03622	2062
	M1	182.03949	2063
	M2	183.04287	2063
**C1809**	M0	181.05081	1549
	M1	182.05405	1550
	M2	183.05739	1549
	M3	184.06083	1549
	M4	185.06415	1550
	M5	186.06749	1551
**C1853**	M0	184.00166	1664
	M1	185.00504	1664
	M2	186.00834	1656
	M3	187.01182	1664
**C1866**	M0	184.98571	1742
	M1	185.98908	1742
	M2	186.99269	1741
	M3	187.99577	1741
**C1851**	M0	183.99614	2118
	M1	184.99939	2117
	M2	186.00283	2117
	M3	187.00615	2117
**C1897**	M0	186.04091	579
	M1	187.04425	579
	M2	188.04759	579
	M3	189.05097	579
	M4	190.05435	579
**C1924**	M0	187.07267	165
	M1	188.07599	162
	M2	189.07924	161
	M3	190.08283	163
	M4	191.08619	161
	M5	192.08939	164
**C1907**	M0	186.99006	1742
	M2	188.99655	1742
	M3	189.99995	1742
**C1934**	M0	188.05651	541
	M1	189.05989	541
	M2	190.06328	540
	M3	191.06660	541
	M4	192.06996	540
	M5	193.07326	540
	M6	194.07670	540
	M7	195.08029	540
**C1944**	M0	189.04067	199
	M1	190.04401	199

	M2	191.04734	201
	M3	192.05069	200
	M4	193.05405	202
**C1951**	M0	189.07699	1041
	M1	190.08036	1041
	M2	191.08372	1041
	M3	192.08708	1041
	M4	193.09038	1041
	M5	194.09378	1040
	M6	195.09717	1040
	M7	196.10051	1040
**C1954**	M0	189.08825	1494
	M1	190.09162	1494
	M2	191.09498	1494
	M3	192.09836	1494
	M4	193.10164	1494
	M5	194.10497	1494
**C1956**	M0	189.50417	1917
	M1	190.50759	1918
	M2	191.51088	1919
	M3	192.51412	1919
**C1984**	M0	191.01987	1816
	M1	192.02324	1816
	M2	193.02651	1816
	M3	194.02985	1816
	M4	195.03318	1816
	M5	196.03663	1816
	M6	197.04027	1816
**C1991**	M0	191.05629	637
	M1	192.05974	638
	M2	193.06295	637
	M3	194.06617	637
	M4	195.06960	637
**C1992**	M0	191.05676	170
	M3	194.06624	174
**C2014**	M0	192.06680	468
	M3	195.07672	468
	M4	196.08023	469
	M5	197.08359	469
	M6	198.08696	468
	M7	199.09038	468
**C2096**	M0	195.00656	1829
	M1	196.00998	1830
	M2	197.01323	1828
	M3	198.01663	1828
	M4	199.01998	1828

**C2102**	M0	195.05108	176
	M1	196.05443	176
	M2	197.05782	176
	M3	198.06124	170
	M4	199.06503	176
	M5	200.06792	176
	M6	201.07121	176
**C2103**	M0	195.05115	257
	M1	196.05452	258
	M2	197.05792	258
	M5	200.06801	258
	M6	201.07136	258
**C2104**	M0	195.05119	334
	M1	196.05457	334
	M2	197.05799	334
	M5	200.06806	333
	M6	201.07137	334
**C2105**	M0	195.05119	425
	M1	196.05458	425
	M6	201.07140	425
**C2110**	M0	195.05115	586
	M1	196.05455	586
**C2106**	M0	195.05116	773
	M1	196.05457	771
**C2108**	M0	195.05113	815
	M1	196.05453	813
**C2101**	M0	195.03371	233
	M2	197.04017	234
	M3	198.04351	237
**C2152**	M0	197.02241	361
	M1	198.02571	360
	M6	203.04253	361
**C2154**	M0	197.02236	263
	M1	198.02578	264
**C2028**	M0	193.02368	1816
	M5	198.04097	1816
	M6	199.04414	1813
**C2159**	M0	197.05515	176
	M1	198.05882	176
	M6	203.07544	176
**C2158**	M0	197.05526	334
	M1	198.05867	333
**C2172**	M0	198.01749	1559
	M1	199.02066	1559
	M2	200.02406	1559
	M3	201.02752	1559

	M4	202.03098	1559
**C2187**	M0	199.00181	889
	M1	200.00495	888
	M2	201.00814	888
	M3	202.01162	888
	M4	203.01508	888
**C2193**	M0	199.01944	360
	M1	200.02273	361
**C2218**	M0	200.05665	532
	M1	201.06000	531
	M2	202.06336	531
	M3	203.06674	531
	M4	204.07002	531
	M5	205.07340	531
	M6	206.07683	531
	M7	207.08020	531
**C2245**	M0	201.11344	1705
	M1	202.11682	1704
**C2269**	M0	202.07231	573
	M1	203.07569	573
	M2	204.07899	573
	M3	205.08234	573
	M4	206.08571	573
	M5	207.08907	573
	M6	208.09249	573
	M7	209.09579	573
**C2219**	M0	200.05672	755
	M2	202.06341	752
	M4	204.07011	752
**C2298**	M0	203.09273	721
	M1	204.09600	721
	M2	205.09948	718
	M3	206.10283	718
	M4	207.10619	718
**C2299**	M0	203.09266	311
	M1	204.09600	312
	M2	205.09944	311
	M3	206.10283	311
	M4	207.10622	311
	M5	208.10960	311
**C2302**	M0	203.12904	474
	M1	204.13248	474
	M2	205.13577	474
	M3	206.13920	474
	M4	207.14255	474
	M5	208.14594	474

	M6	209.14933	473
**C2329**	M0	205.02906	713
	M1	206.03242	717
	M2	207.03578	716
	M3	208.03918	716
	M4	209.04252	717
**C2331**	M0	205.03554	1759
	M1	206.03892	1760
	M2	207.04233	1759
	M3	208.04567	1759
	M4	209.04908	1759
	M5	210.05246	1760
	M6	211.05563	1761
	M7	212.05936	1761
**C2334**	M0	205.04676	655
	M1	206.05012	655
	M2	207.05350	655
	M3	208.05692	655
	M4	209.06029	655
**C2337**	M0	205.07195	781
	M1	206.07536	781
	M2	207.07864	779
	M3	208.08210	779
	M4	209.08545	779
	M5	210.08886	779
	M6	211.09222	779
	M7	212.09555	780
**C2310**	M0	204.01509	1959
	M3	207.02517	1958
**C2356**	M0	206.06699	164
	M1	207.07067	162
**C2375**	M0	207.05125	203
	M1	208.05462	206
	M2	209.05802	201
	M3	210.06148	199
	M4	211.06479	199
	M5	212.06816	202
	M6	213.07154	210
	M7	214.07492	202
**C2361**	M0	206.08249	406
	M1	207.08589	406
	M2	208.08925	406
	M3	209.09261	406
	M4	210.09594	406
	M5	211.09924	407
	M6	212.10273	406

**C2445**	M0	211.00164	1978
	M1	212.00500	1979
	M2	213.00848	1979
	M3	214.01175	1979
**C2429**	M0	210.00806	731
	M2	212.01486	729
**C2449**	M0	211.02508	2132
	M1	212.02853	2132
	M2	213.03177	2132
	M3	214.03522	2131
**C2473**	M0	212.98094	981
	M1	213.98429	981
	M2	214.98763	980
**C2447**	M0	211.00169	1932
	M3	214.01161	1934
**C2481**	M0	213.01729	547
	M1	214.02066	547
	M2	215.02394	547
	M3	216.02723	546
	M4	217.03064	545
**C2491**	M0	213.98526	1573
	M1	214.98865	1573
	M2	215.99192	1572
	M3	216.99519	1572
**C2513**	M0	214.99659	1718
	M1	215.99985	1717
	M2	217.00317	1714
	M3	218.00657	1717
	M4	219.00993	1719
**C2525**	M0	215.03307	257
	M1	216.03635	259
	M2	217.03972	259
	M6	221.05320	259
**C2530**	M0	215.03313	418
	M1	216.03640	417
	M2	217.03975	417
	M6	221.05326	418
**C2524**	M0	215.03310	341
	M1	216.03637	343
	M2	217.03972	340
	M6	221.05325	340
**C2532**	M0	215.03305	715
	M1	216.03638	711
**C2527**	M0	215.03309	596
	M1	216.03637	597
**C2529**	M0	215.03305	166

	M1	216.03629	166
	M2	217.03976	166
	M3	218.04326	162
	M6	221.05314	166
**C2486**	M0	213.08840	1755
	M3	216.09842	1754
	M4	217.10182	1753
**C2552**	M0	216.03372	1871
	M1	217.03715	1871
	M2	218.04057	1870
	M3	219.04397	1870
	M4	220.04735	1871
	M5	221.05065	1870
	M6	222.05417	1870
**C2531**	M0	215.03312	500
	M1	216.03637	502
	M2	217.03955	495
	M3	218.04293	496
	M4	219.04632	496
**C2581**	M0	217.02999	166
	M1	218.03338	166
	M2	219.03694	166
	M6	223.05019	166
**C2582**	M0	217.03004	258
	M1	218.03343	259
	M2	219.03691	257
	M6	223.05030	258
**C2584**	M0	217.03007	341
	M1	218.03347	343
	M2	219.03696	345
	M6	223.05029	340
**C2583**	M0	217.03010	418
	M1	218.03349	417
	M6	223.05034	417
**C2617**	M0	218.06724	532
	M1	219.07063	532
	M2	220.07400	532
	M3	221.07735	531
	M4	222.08073	531
	M5	223.08408	531
	M6	224.08752	531
	M7	225.09094	531
**C2620**	M0	218.10363	189
	M1	219.10700	184
	M2	220.11038	189
	M3	221.11369	183

	M4	222.11709	184
	M5	223.12046	183
	M6	224.12381	183
	M7	225.12721	183
	M8	226.13065	182
**C2618**	M0	218.06727	755
	M1	219.07064	754
	M2	220.07402	752
	M3	221.07737	752
	M4	222.08076	752
	M5	223.08415	751
**C2649**	M0	220.02883	1883
	M1	221.03213	1883
	M2	222.03564	1883
	M3	223.03894	1883
**C2672**	M0	221.04776	312
	M1	222.05124	312
**C2654**	M0	220.06185	420
	M1	221.06532	420
	M2	222.06857	420
	M3	223.07191	420
	M4	224.07533	420
	M5	225.07870	420
	M6	226.08214	420
**C2663**	M0	220.50524	2217
	M2	222.51196	2213
	M3	223.51535	2213
**C2668**	M0	220.99917	225
	M1	222.00255	224
	M2	223.00592	225
	M3	224.00927	221
	M4	225.01254	225
**C2697**	M0	222.04449	976
	M1	223.04781	976
	M2	224.05126	975
	M3	225.05460	975
	M4	226.05800	975
	M5	227.06137	974
**C2671**	M0	221.04581	1687
	M2	223.05255	1690
	M3	224.05600	1690
	M4	225.05929	1688
	M5	226.06256	1689
	M6	227.06598	1688
	M7	228.06935	1688
	M8	229.07268	1688

**C2715**	M0	222.99638	225
	M1	224.00000	227
	M2	225.00327	224
	M3	226.00663	227
	M4	227.00991	224
**C2720**	M0	223.04340	310
	M1	224.04676	310
**C2760**	M0	225.00813	771
	M1	226.01149	768
**C2762**	M0	225.01731	255
	M1	226.02068	255
	M2	227.02404	251
	M3	228.02735	248
	M4	229.03059	248
	M5	230.03400	248
	M6	231.03753	255
**C2763**	M0	225.01732	201
	M1	226.02066	201
	M2	227.02403	197
	M6	231.03746	202
**C2764**	M0	225.01728	1982
	M1	226.02072	1982
**C2767**	M0	225.04083	728
	M1	226.04421	728
	M2	227.04760	726
	M3	228.05100	726
	M4	229.05422	725
	M5	230.05756	725
**C2773**	M0	225.06194	205
	M1	226.06536	205
	M6	231.08209	204
**C2782**	M0	226.00306	780
	M1	227.00661	778
	M2	228.00986	778
**C2765**	M0	225.01732	316
	M1	226.02075	319
	M2	227.02407	320
	M3	228.02736	324
	M4	229.03059	324
	M6	231.03758	318
**C2790**	M0	226.02999	2181
	M1	227.03338	2181
	M2	228.03672	2181
	M3	229.04000	2181
**C2828**	M0	228.05169	1699
	M1	229.05499	1697

	M2	230.05829	1697
	M3	231.06165	1697
	M4	232.06501	1697
	M5	233.06837	1697
	M6	234.07182	1697
	M7	235.07515	1697
	M8	236.07856	1697
**C2811**	M0	227.06776	471
	M2	229.07436	470
	M3	230.07779	477
	M4	231.08119	478
	M5	232.08447	478
	M6	233.08775	478
**C2836**	M0	229.01215	1090
	M1	230.01552	1089
	M2	231.01889	1087
	M3	232.02227	1088
	M4	233.02564	1087
	M5	234.02907	1086
**C2837**	M0	229.01217	540
	M1	230.01563	540
	M2	231.01894	540
	M3	232.02217	540
**C2838**	M0	229.01220	1978
	M1	230.01557	1979
**C2824**	M0	228.02826	778
	M1	229.03130	775
	M2	230.03483	776
**C2849**	M0	229.04690	712
	M1	230.05030	710
	M2	231.05366	709
	M3	232.05702	709
	M4	233.06045	709
**C2816**	M0	227.10413	1494
	M2	229.11073	1493
	M3	230.11411	1493
	M4	231.11762	1493
**C2874**	M0	230.06730	674
	M1	231.07072	674
	M2	232.07408	673
	M3	233.07754	673
	M4	234.08082	673
	M5	235.08417	673
**C2642**	M0	219.97875	984
	M13	233.02135	979
	M14	234.02484	979

	M15	235.02846	979
	M16	236.03180	979
	M17	237.03521	979
	M18	238.03851	979
**C2890**	M0	231.02799	224
	M2	233.03473	225
	M3	234.03812	224
	M4	235.04145	224
**C2940**	M0	233.01287	1831
	M1	234.01634	1831
	M2	235.01963	1831
	M3	236.02304	1831
	M4	237.02641	1831
	M5	238.02982	1831
	M6	239.03317	1831
	M7	240.03647	1831
**C2948**	M0	233.03782	2173
	M1	234.04186	2174
	M2	235.04522	2174
	M0	229.04689	1878
	M1	230.05028	1879
	M5	234.06384	1879
**C2967**	M0	233.96616	694
	M1	234.96949	689
	M2	235.97285	689
	M3	236.97627	689
**C2975**	M0	234.04454	1871
	M1	235.04797	1871
	M2	236.05131	1870
	M3	237.05470	1870
	M4	238.05808	1870
	M5	239.06146	1870
	M6	240.06482	1870
**C2996**	M0	235.00174	1773
	M1	236.00511	1773
**C3043**	M0	237.06202	201
	M1	238.06538	200
	M2	239.06879	199
	M3	240.07216	200
	M4	241.07550	200
	M5	242.07887	200
	M6	243.08211	201
	M8	245.08880	204
**C3070**	M0	238.99671	1755
	M1	240.00014	1755
**C3097**	M0	240.05182	2415

	M1	241.05507	2413
	M2	242.05858	2411
	M3	243.06182	2412
	M4	244.06519	2412
	M5	245.06857	2411
	M6	246.07196	2413
	M7	247.07535	2412
	M8	248.07878	2414
**C3104**	M0	240.97752	2209
	M1	241.98092	2209
	M2	242.98408	2208
	M3	243.98749	2203
	M4	244.99065	2203
**C3106**	M0	241.01243	239
	M1	242.01579	240
	M2	243.01903	248
	M6	247.03261	240
**C3107**	M0	241.01236	313
	M1	242.01577	312
	M2	243.01901	313
	M3	244.02214	324
	M4	245.02552	319
**C3105**	M0	241.01240	2087
	M1	242.01580	2087
**C3110**	M0	241.02947	338
	M1	242.03292	340
**C3122**	M0	241.99950	2218
	M1	243.00277	2218
	M2	244.00606	2218
	M3	245.00928	2217
	M4	246.01283	2217
**C3130**	M0	242.04407	139
	M1	243.04731	139
	M2	244.05077	139
	M5	247.06098	139
	M6	248.06423	139
**C3145**	M0	243.02784	481
	M1	244.03129	481
	M2	245.03470	481
	M3	246.03794	480
	M4	247.04136	480
	M5	248.04485	480
	M6	249.04820	481
**C3147**	M0	243.02797	1981
	M1	244.03130	1982
**C3146**	M0	243.02792	900

	M1	244.03131	898
	M2	245.03452	895
	M3	246.03799	896
	M4	247.04147	894
	M5	248.04471	894
**C3153**	M0	243.06264	274
	M1	244.06607	274
	M2	245.06943	274
	M3	246.07281	274
	M4	247.07618	274
	M5	248.07949	274
	M6	249.08283	274
	M7	250.08625	270
**C3193**	M0	244.04106	139
	M1	245.04446	139
	M5	249.05774	141
	M6	250.06137	139
**C3117**	M0	241.39984	2315
	M4	245.41327	2314
	M5	246.41662	2313
	M6	247.41990	2316
**C3205**	M0	244.99901	1919
	M1	246.00240	1919
	M2	247.00564	1919
	M3	248.00913	1919
**C3206**	M0	245.00722	1686
	M1	246.01068	1685
	M2	247.01391	1685
	M3	248.01735	1685
	M4	249.02062	1685
**C3245**	M0	246.06242	1699
	M1	247.06566	1697
	M2	248.06912	1698
	M3	249.07251	1698
	M4	250.07587	1698
	M5	251.07917	1697
	M6	252.08252	1697
	M7	253.08587	1697
	M8	254.08925	1697
**C3266**	M0	247.02308	208
	M1	248.02637	202
	M2	249.02977	202
	M3	250.03313	203
	M4	251.03650	203
**C3277**	M0	247.06898	576
	M1	248.07216	576

	M2	249.07560	576
	M3	250.07912	576
	M4	251.08227	576
**C3303**	M0	248.07812	453
	M1	249.08153	453
	M2	250.08473	453
	M4	252.09164	452
**C3352**	M0	251.07785	168
	M1	252.08110	176
	M2	253.08449	176
	M3	254.08785	176
	M4	255.09120	176
	M5	256.09454	175
	M6	257.09790	176
	M7	258.10109	176
**C3361**	M0	252.05537	428
	M1	253.05871	427
	M2	254.06196	427
	M3	255.06539	427
	M4	256.06880	429
**C3370**	M0	252.49122	2187
	M1	253.49461	2187
	M2	254.49799	2187
	M3	255.50131	2187
	M4	256.50460	2185
**C3376**	M0	252.98728	1534
	M1	253.99064	1533
	M2	254.99399	1532
**C3379**	M0	252.99304	2187
	M1	253.99632	2187
	M2	254.99963	2187
**C3381**	M0	253.01233	1773
	M1	254.01582	1773
	M2	255.01921	1773
	M3	256.02258	1773
	M4	257.02584	1773
	M5	258.02917	1773
	M6	259.03261	1772
	M7	260.03591	1772
**C3384**	M0	253.04719	1944
	M1	254.05058	1944
	M2	255.05393	1944
	M3	256.05735	1944
	M4	257.06069	1944
	M5	258.06390	1944
**C3422**	M0	255.00297	678

	M1	256.00635	678
	M2	257.00962	677
	M3	258.01296	677
	M4	259.01636	676
**C3414**	M0	254.69849	2190
	M2	256.70521	2190
	M3	257.70860	2190
**C3455**	M0	257.00735	1755
	M1	258.01064	1755
	M2	259.01401	1755
	M3	260.01734	1755
	M4	261.02067	1755
	M5	262.02415	1754
**C3461**	M0	257.05516	140
	M1	258.05836	140
	M2	259.06109	139
	M6	263.07530	141
	M7	264.07865	141
**C3482**	M0	258.02944	230
	M1	259.03288	230
**C3485**	M0	258.03897	904
	M1	259.04229	909
**C3503**	M0	259.01342	303
	M1	260.01680	303
**C3507**	M0	259.02292	2087
	M1	260.02631	2087
	M2	261.02976	2087
	M3	262.03311	2087
	M4	263.03645	2087
	M5	264.03974	2087
	M6	265.04320	2087
**C3506**	M0	259.02281	890
	M1	260.02624	889
	M2	261.02965	888
	M3	262.03283	888
	M4	263.03640	888
	M5	264.03977	887
	M6	265.04306	887
**C3509**	M0	259.02283	957
	M1	260.02629	956
	M2	261.02969	955
	M3	262.03285	952
	M4	263.03637	953
	M5	264.03966	953
	M6	265.04311	954
**C3510**	M0	259.02293	523

	M1	260.02631	523
	M2	261.02979	523
	M3	262.03309	521
	M4	263.03653	521
	M5	264.03996	523
	M6	265.04319	523
**C3508**	M0	259.02287	1023
	M1	260.02629	1022
	M2	261.02968	1022
	M3	262.03303	1022
	M4	263.03640	1022
	M5	264.03965	1021
	M6	265.04312	1020
**C3511**	M0	259.02295	591
	M1	260.02634	591
	M3	262.03301	591
**C3512**	M0	259.02296	1980
	M1	260.02635	1981
**C3513**	M0	259.02293	797
	M1	260.02633	800
	M2	261.02965	799
	M3	262.03297	799
	M4	263.03635	798
	M5	264.03976	798
**C3523**	M0	259.05221	139
	M1	260.05556	140
	M2	261.05885	141
	M6	265.07231	141
**C3553**	M0	261.00248	516
	M1	262.00588	516
	M3	264.01243	515
**C3565**	M0	261.03860	514
	M1	262.04199	514
	M2	263.04539	514
	M3	264.04876	513
	M4	265.05201	513
	M5	266.05531	513
	M6	267.05877	513
**C3566**	M0	261.03876	210
	M1	262.04210	201
	M2	263.04548	201
	M3	264.04884	201
	M4	265.05217	202
**C3590**	M0	262.05744	1776
	M1	263.06074	1776
	M2	264.06415	1776

	M3	265.06755	1776
	M4	266.07089	1776
	M5	267.07417	1776
	M7	269.08084	1776
**C3597**	M0	262.97330	776
	M1	263.97666	774
	M2	264.97996	776
	M3	265.98332	776
	M4	266.98674	774
**C3615**	M0	263.96858	2063
	M1	264.97195	2062
	M2	265.97532	2062
	M3	266.97867	2064
**C3633**	M0	264.07308	249
	M1	265.07649	250
**C3575**	M0	261.58276	2163
	M3	264.59285	2163
	M4	265.59619	2164
	M5	266.59962	2163
	M6	267.60298	2164
**C3639**	M0	264.94310	1890
	M1	265.94653	1890
	M3	267.95330	1889
**C3640**	M0	264.95259	2151
	M1	265.95597	2151
	M2	266.95921	2151
	M3	267.96269	2151
**C3641**	M0	264.95250	1890
	M1	265.95584	1890
	M3	267.96252	1890
**C3573**	M0	261.08103	2163
	M4	265.09454	2163
	M5	266.09792	2163
	M6	267.10129	2163
	M7	268.10463	2163
**C3556**	M0	261.02714	890
	M6	267.04738	887
**C3668**	M0	266.01683	2110
	M2	268.02364	2110
	M3	269.02690	2110
**C3605**	M0	263.04283	513
	M5	268.05971	505
**C3687**	M0	267.07285	238
	M1	268.07612	238
**C3644**	M0	265.03961	493
	M4	269.05292	489

**C3752**	M0	271.09421	1494
	M1	272.09763	1494
	M2	273.10094	1494
	M3	274.10432	1494
	M4	275.10766	1494
	M5	276.10997	1494
	M6	277.11333	1493
	M7	278.11670	1493
	M8	279.12002	1493
**C3770**	M0	273.00239	1734
	M1	274.00578	1734
	M2	275.00921	1734
	M3	276.01121	1734
	M4	277.01476	1734
	M6	279.02155	1734
**C3771**	M0	273.00409	633
	M1	274.00756	634
	M2	275.01082	633
**C3776**	M0	273.03387	1790
	M1	274.03727	1789
	M2	275.04066	1789
	M3	276.04293	1789
	M4	277.04626	1789
	M5	278.04952	1789
	M6	279.05271	1789
**C3747**	M0	271.05788	738
	M1	272.06121	741
	M3	274.06798	744
**C3765**	M0	272.08948	1708
	M1	273.09286	1710
	M2	274.09628	1709
	M3	275.09960	1710
	M4	276.10193	1710
**C3796**	M0	273.53557	1790
	M1	274.53890	1789
	M2	275.54119	1789
	M3	276.54457	1789
	M4	277.54784	1789
	M5	278.55119	1789
**C3819**	M0	275.00851	1205
	M1	276.01094	1203
**C3822**	M0	275.01796	1655
	M1	276.02040	1655
**C3864**	M0	276.98801	807
	M1	277.99141	807
	M2	278.99475	805

	M3	279.99813	805
	M4	281.00146	805
	M5	282.00475	805
**C3866**	M0	277.01161	2217
	M1	278.01500	2217
	M2	279.01844	2217
	M3	280.02171	2216
**C3870**	M0	277.03265	197
	M1	278.03605	196
	M3	280.04282	196
	M4	281.04619	196
**C3871**	M0	277.03270	257
	M1	278.03606	259
**C3872**	M0	277.03750	421
	M1	278.04100	420
**C3878**	M0	277.06744	775
	M1	278.07082	775
	M2	279.07421	773
	M3	280.07756	772
	M4	281.08093	772
	M5	282.08428	772
	M6	283.08763	771
	M7	284.09087	771
**C3906**	M0	278.98097	146
	M1	279.98442	147
**C3909**	M0	279.01211	215
	M1	280.01548	215
**C3915**	M0	279.05258	322
	M1	280.05600	322
**C3899**	M0	278.08780	166
	M2	280.09453	167
**C3953**	M0	281.97828	1664
	M1	282.98147	1657
	M2	283.98480	1656
**C3959**	M0	282.01997	1959
	M1	283.02332	1959
	M2	284.02677	1959
	M3	285.03012	1959
	M4	286.03346	1958
	M5	287.03666	1958
	M6	288.03986	1957
**C3929**	M0	280.02235	1936
	M2	282.02932	1936
	M3	283.03250	1934
	M4	284.03588	1934
	M5	285.03909	1934

**C3966**	M0	282.52175	1959
	M1	283.52509	1959
	M2	284.52841	1959
	M3	285.53177	1958
	M4	286.53514	1958
	M5	287.53845	1958
	M6	288.54134	1957
**C3971**	M0	282.95291	1743
	M1	283.95645	1742
	M2	284.95924	1743
	M3	285.96291	1742
**C3972**	M0	282.96223	1741
	M1	283.96569	1741
	M2	284.96872	1741
	M3	285.97235	1741
**C4012**	M0	285.01858	317
	M1	286.02196	318
**C3996**	M0	284.05402	453
	M1	285.05753	452
	M2	286.06119	452
**C3967**	M0	282.53026	2108
	M4	286.54367	2110
**C4036**	M0	287.01715	1731
	M1	288.02055	1731
	M2	289.02391	1731
	M3	290.02726	1731
	M4	291.03061	1731
	M5	292.03400	1731
	M6	293.03720	1731
	M7	294.04073	1730
**C4044**	M0	287.06500	139
	M1	288.06833	140
**C4038**	M0	287.03417	1944
	M2	289.04096	1944
	M3	290.04432	1944
	M4	291.04768	1944
**C4045**	M0	287.08837	720
	M1	288.09177	719
	M2	289.09511	717
	M3	290.09842	717
	M4	291.10172	722
	M5	292.10492	718
	M6	293.10836	717
	M7	294.11179	717
	M8	295.11411	722
	M9	296.11752	722

**C4068**	M0	289.03282	1191
	M1	290.03618	1191
	M2	291.03956	1190
	M3	292.04291	1189
	M4	293.04627	1189
	M5	294.04965	1189
	M6	295.05190	1188
	M7	296.05513	1187
**C4067**	M0	289.03283	999
	M1	290.03622	998
	M2	291.03960	997
	M3	292.04293	997
	M4	293.04628	997
	M5	294.04965	997
	M6	295.05182	994
**C4069**	M0	289.03288	534
	M1	290.03614	534
	M2	291.03962	528
	M3	292.04291	528
	M4	293.04627	528
	M5	294.04960	528
**C4071**	M0	289.03283	2118
	M1	290.03625	2118
	M3	292.04288	2117
	M4	293.04611	2117
	M5	294.04958	2118
**C4037**	M0	287.03410	964
	M2	289.04085	964
	M3	290.04418	965
	M4	291.04760	965
	M5	292.05098	965
	M6	293.05436	965
	M7	294.05773	965
**C4056**	M0	288.07230	1791
	M1	289.07574	1791
	M2	290.07906	1791
	M3	291.08246	1791
	M4	292.08588	1791
	M5	293.08920	1791
	M6	294.09259	1791
	M7	295.09477	1790
	M8	296.09817	1791
	M9	297.10141	1790
**C4083**	M0	289.10394	1494
	M1	290.10734	1494
	M2	291.11069	1494

	M3	292.11403	1494
	M4	293.11740	1494
	M5	294.12070	1494
	M6	295.12300	1494
	M7	296.12636	1494
	M8	297.12972	1494
	M9	298.13307	1494
	M10	299.13627	1493
**C4086**	M0	289.12258	252
	M1	290.12607	253
	M2	291.12934	253
	M3	292.13266	253
	M4	293.13606	252
	M5	294.13830	252
	M6	295.14161	253
	M7	296.14486	253
**C4120**	M0	290.96751	2088
	M1	291.97084	2089
	M2	292.97427	2089
	M3	293.97750	2089
	M4	294.98002	2089
	M5	295.98325	2089
**C4118**	M0	290.96749	1838
	M1	291.97094	1838
	M2	292.97419	1838
	M3	293.97768	1838
**C4105**	M0	290.08800	222
	M1	291.09150	223
	M2	292.09472	222
	M3	293.09818	223
	M4	294.10150	223
**C4119**	M0	290.96745	2182
	M2	292.97424	2180
	M3	293.97755	2180
**C4132**	M0	291.06549	1754
	M1	292.06897	1754
	M2	293.07228	1754
	M3	294.07564	1754
	M4	295.07788	1754
	M5	296.08120	1754
	M6	297.08456	1754
	M7	298.08784	1754
**C4184**	M0	292.10364	253
	M1	293.10705	253
	M2	294.11043	253
	M3	295.11264	253

	M4	296.11601	253
	M5	297.11936	253
	M6	298.12268	253
	M7	299.12602	253
	M8	300.12938	253
	M9	301.13275	253
**C4109**	M0	290.09933	447
	M3	293.10944	456
**C4255**	M0	294.08295	729
	M1	295.08505	729
	M2	296.08851	726
	M3	297.09186	726
	M4	298.09527	726
	M5	299.09859	726
	M6	300.10200	725
	M7	301.10533	725
	M8	302.10867	725
	M9	303.11195	724
**C4256**	M0	294.08295	302
	M1	295.08499	300
	M2	296.08848	302
**C4332**	M0	296.93335	1796
	M1	297.93669	1796
	M2	298.94008	1796
	M3	299.94337	1796
**C4359**	M0	298.06851	1556
	M1	299.07186	1557
	M2	300.07521	1556
	M3	301.07857	1556
	M4	302.08192	1556
	M5	303.08522	1556
**C4326**	M0	296.49752	2196
	M3	299.50748	2194
	M4	300.51088	2194
**C4351**	M0	297.82828	2133
	M2	299.83530	2133
	M3	300.83863	2133
	M4	301.84196	2133
	M5	302.84527	2133
	M6	303.84860	2133
**C4349**	M0	297.32689	2133
	M3	300.33694	2133
	M4	301.34032	2133
	M5	302.34362	2133
	M6	303.34698	2133
**C4350**	M0	297.57765	2132

	M2	299.58448	2132
	M3	300.58777	2133
	M4	301.59111	2133
	M6	303.59780	2133
**C4394**	M0	300.03836	494
	M1	301.04191	494
	M2	302.04551	493
	M6	306.05868	492
**C4397**	M0	300.04779	919
	M1	301.05114	917
	M2	302.05453	917
	M3	303.05786	916
**C4409**	M0	300.89879	1547
	M1	301.90161	1547
**C4426**	M0	301.96462	2194
	M1	302.96825	2192
**C4437**	M0	302.06347	216
	M1	303.06687	217
	M2	304.07024	218
**C4386**	M0	299.51549	2090
	M3	302.52519	2092
	M4	303.52873	2091
**C4448**	M0	302.53233	1863
	M1	303.53565	1863
	M2	304.53913	1863
	M3	305.54245	1863
	M4	306.54577	1863
	M5	307.54911	1862
	M6	308.55245	1862
	M7	309.55569	1862
**C4453**	M0	302.94327	1091
	M1	303.94664	1089
**C4464**	M0	303.03410	1863
	M1	304.03746	1863
	M2	305.04081	1863
	M3	306.04412	1863
	M4	307.04744	1863
	M5	308.05083	1862
	M6	309.05418	1862
**C4395**	M0	300.04771	1918
	M4	304.06127	1918
	M5	305.06445	1918
**C4490**	M0	304.04262	138
	M1	305.04608	139
	M2	306.04958	140
	M6	310.06280	139

**C4441**	M0	302.09810	778
	M3	305.10828	775
	M4	306.11157	775
	M5	307.11498	783
	M6	308.11837	782
**C4507**	M0	305.04376	1944
	M1	306.04722	1944
	M2	307.05053	1944
	M3	308.05378	1944
	M4	309.05719	1944
	M5	310.06054	1944
	M6	311.06386	1944
	M7	312.06725	1944
	M8	313.07056	1944
	M9	314.07385	1944
**C4491**	M0	304.05971	1882
	M1	305.06316	1882
	M2	306.06647	1882
	M3	307.06983	1882
	M4	308.07312	1882
	M5	309.07641	1881
**C4515**	M0	305.08015	1742
	M1	306.08359	1742
	M2	307.08674	1742
	M3	308.09007	1742
	M4	309.09335	1742
	M5	310.09671	1742
	M6	311.10025	1742
	M7	312.10374	1742
**C4510**	M0	305.06330	203
	M1	306.06665	203
	M2	307.07006	203
	M3	308.07339	203
	M4	309.07672	203
	M5	310.08002	203
	M6	311.08336	202
	M7	312.08673	203
	M8	313.08999	200
**C4533**	M0	306.07536	1708
	M1	307.07890	1708
	M2	308.08216	1709
	M3	309.08541	1710
	M4	310.08881	1709
	M5	311.09212	1709
	M6	312.09545	1709
	M7	313.09880	1709

	M8	314.10207	1710
	M9	315.10529	1715
**C4534**	M0	306.07538	1846
	M1	307.07884	1846
	M2	308.08215	1846
	M3	309.08549	1846
	M4	310.08883	1846
	M5	311.09209	1846
	M6	312.09532	1845
	M7	313.09852	1845
	M8	314.10185	1845
	M9	315.10518	1845
**C4535**	M0	306.07536	1901
	M1	307.07891	1903
	M2	308.08205	1906
	M3	309.08546	1903
	M4	310.08876	1905
	M5	311.09200	1906
	M6	312.09517	1906
	M7	313.09847	1906
	M8	314.10181	1907
	M9	315.10513	1907
**C4537**	M0	306.07534	1969
	M1	307.07871	1965
	M2	308.08209	1963
	M3	309.08541	1964
	M4	310.08870	1964
	M5	311.09196	1965
	M6	312.09523	1965
	M7	313.09855	1965
	M8	314.10188	1965
	M9	315.10519	1964
**C4514**	M0	305.08013	719
	M1	306.08364	718
	M2	307.08695	716
	M3	308.09025	716
	M4	309.09358	716
	M5	310.09690	716
	M6	311.10029	716
	M7	312.10376	716
**C4499**	M0	304.11388	431
	M1	305.11720	429
	M2	306.12061	430
	M3	307.12394	430
	M4	308.12728	430
	M5	309.13066	430

**C4555**	M0	307.03294	443
	M1	308.03616	443
**C4569**	M0	307.07801	261
	M1	308.08167	261
	M2	309.08492	262
	M3	310.08833	262
	M4	311.09164	262
	M5	312.09480	262
	M6	313.09815	262
**C4620**	M0	308.97706	2130
	M1	309.98044	2131
	M2	310.98374	2131
	M3	311.98711	2132
	M4	312.99046	2133
**C4622**	M0	308.97705	2027
	M1	309.98042	2027
**C4621**	M0	308.97707	1978
	M1	309.98045	1979
**C4558**	M0	307.03960	1944
	M1	308.04299	1944
	M3	310.05044	1944
	M4	311.05349	1944
	M5	312.05665	1944
	M6	313.05989	1944
**C4541**	M0	306.07543	1766
	M1	307.07885	1767
	M3	309.08552	1766
	M4	310.08882	1766
**C4606**	M0	308.09753	755
	M1	309.10096	754
	M2	310.10432	752
	M3	311.10767	752
	M4	312.11101	752
	M5	313.11437	752
	M6	314.11772	751
	M7	315.12110	750
	M8	316.12446	750
	M9	317.12783	750
	M10	318.13113	750
	M11	319.13433	750
**C4538**	M0	306.07539	2067
	M5	311.09208	2070
	M6	312.09539	2070
**C4539**	M0	306.07532	2144
	M5	311.09218	2145
**C4649**	M0	309.50524	2098

	M2	311.51212	2101
	M3	312.51532	2102
	M4	313.51869	2102
	M5	314.52204	2100
	M6	315.52521	2099
**C4278**	M0	294.93019	223
	M17	311.98737	227
**C4685**	M0	311.02577	1526
	M1	312.02928	1526
	M2	313.03249	1526
	M3	314.03587	1526
	M4	315.03920	1525
	M5	316.04259	1525
	M6	317.04596	1525
	M7	318.04932	1524
	M8	319.05262	1524
	M9	320.05584	1531
**C4753**	M0	313.04301	550
	M1	314.04662	550
	M2	315.04980	550
	M3	316.05310	550
	M4	317.05648	550
	M5	318.05990	549
	M6	319.06313	547
**C4710**	M0	311.50289	2195
	M3	314.51291	2192
	M4	315.51623	2192
**C4756**	M0	313.07793	738
	M2	315.08459	735
	M3	316.08795	734
	M4	317.09133	734
	M5	318.09464	734
	M6	319.09810	734
	M7	320.10133	734
**C4797**	M0	314.86426	1546
	M1	315.86672	1545
**C4747**	M0	312.99034	211
	M3	316.00058	208
**C4772**	M0	313.99989	2165
	M2	316.00659	2165
	M3	317.00995	2165
**C4809**	M0	315.04028	520
	M1	316.04377	520
**C4812**	M0	315.05713	677
	M1	316.06062	677
	M2	317.06391	676

	M3	318.06727	676
	M4	319.07062	676
	M5	320.07392	676
	M6	321.07719	676
	M7	322.08041	676
	M8	323.08370	675
**C4849**	M0	317.02679	1726
	M1	318.03021	1726
	M2	319.03359	1725
	M3	320.03689	1725
	M4	321.03999	1725
	M5	322.04348	1725
**C4850**	M0	317.04503	520
	M1	318.04865	525
**C4877**	M0	318.51072	2183
	M1	319.51336	2183
	M2	320.51745	2183
	M3	321.52078	2183
	M4	322.52411	2183
	M5	323.52740	2182
	M6	324.53063	2182
**C4887**	M0	319.04256	725
	M1	320.04592	723
	M2	321.04925	727
	M3	322.05255	726
	M4	323.05589	727
	M5	324.05937	727
	M6	325.06268	726
**C4891**	M0	319.05371	138
	M1	320.05703	140
	M2	321.06043	140
	M6	325.07385	140
**C4896**	M0	319.07725	1077
	M1	320.08066	1077
	M2	321.08398	1076
	M3	322.08735	1076
	M4	323.09073	1076
	M5	324.09403	1076
	M6	325.09736	1074
	M7	326.10074	1073
	M8	327.10415	1074
	M9	328.10742	1073
**C4895**	M0	319.07721	814
	M1	320.08064	814
	M2	321.08401	811
	M3	322.08734	811

	M4	323.09070	811
	M5	324.09400	812
	M6	325.09731	811
	M7	326.10071	811
	M8	327.10408	811
**C4834**	M0	316.02176	2371
	M5	321.03859	2374
	M6	322.04194	2369
**C4897**	M0	319.07708	653
	M1	320.08068	651
	M2	321.08408	651
**C4932**	M0	320.91727	1742
	M1	321.92067	1742
**C4941**	M0	321.04825	1829
	M1	322.05174	1829
	M2	323.05504	1829
	M3	324.05837	1829
	M4	325.06172	1829
	M5	326.06506	1828
	M6	327.06841	1828
	M7	328.07176	1828
	M8	329.07511	1828
	M9	330.07840	1828
	M10	331.08094	1828
**C4945**	M0	321.05942	790
	M1	322.06294	787
	M2	323.06618	787
	M3	324.06943	791
	M4	325.07278	791
	M5	326.07622	791
	M6	327.07963	791
	M7	328.08300	790
**C4969**	M0	322.04344	692
	M1	323.04702	693
	M2	324.05029	691
	M3	325.05363	691
	M4	326.05698	691
	M5	327.06033	691
	M6	328.06359	691
**C4995**	M0	322.99283	1982
	M1	323.99620	1981
	M2	324.99957	1981
	M3	326.00241	1981
	M4	327.00625	1981
	M5	328.00955	1981
	M6	329.01303	1982

**C4999**	M0	323.02753	1936
	M1	324.03099	1936
	M2	325.03430	1936
	M3	326.03760	1935
	M4	327.04090	1935
	M5	328.04421	1935
	M6	329.04755	1935
	M7	330.05090	1935
	M8	331.05424	1935
	M9	332.05708	1934
**C5000**	M0	323.02747	2178
	M1	324.03055	2179
	M2	325.03411	2178
	M3	326.03756	2178
	M4	327.04096	2177
	M5	328.04429	2177
	M6	329.04763	2177
	M7	330.05098	2177
**C4980**	M0	322.06892	263
	M2	324.07562	263
**C5016**	M0	323.10606	516
	M1	324.10924	516
	M2	325.11267	516
	M3	326.11599	516
	M4	327.11944	516
	M5	328.12303	516
	M6	329.12609	516
	M7	330.12946	516
**C5091**	M0	326.97821	1089
	M1	327.98160	1088
	M2	328.98482	1086
	M3	329.98824	1087
	M4	330.99158	1086
	M5	331.99495	1085
**C5093**	M0	326.98780	1090
	M1	327.99126	1089
	M2	328.99460	1087
	M3	329.99790	1088
	M4	331.00109	1087
	M5	332.00445	1086
**C5117**	M0	328.04424	1040
	M1	329.04772	1040
	M2	330.05096	1039
	M3	331.05420	1038
	M4	332.05769	1038
	M5	333.06102	1038

	M6	334.06438	1037
	M7	335.06776	1037
	M8	336.07097	1040
**C5099**	M0	327.05899	1493
	M1	328.06243	1493
	M2	329.06574	1493
	M3	330.06908	1493
	M4	331.07245	1493
	M5	332.07585	1492
	M6	333.07915	1492
	M7	334.08249	1492
**C5138**	M0	329.00174	679
	M3	332.01204	681
	M4	333.01536	681
	M5	334.01874	680
	M6	335.02211	680
**C5169**	M0	330.04917	977
	M1	331.05299	976
	M2	332.05598	977
**C5107**	M0	327.09382	600
	M5	332.11041	597
	M6	333.11369	597
	M7	334.11712	596
	M8	335.12040	595
**C5216**	M0	332.02005	1871
	M1	333.02350	1871
	M2	334.02681	1871
	M3	335.03016	1871
	M4	336.03352	1871
	M5	337.03686	1871
**C5239**	M0	333.05824	249
	M1	334.06165	249
	M2	335.06500	249
	M3	336.06823	249
	M4	337.07170	249
	M5	338.07502	250
	M6	339.07839	249
	M7	340.08179	249
	M9	342.08825	249
**C5241**	M0	333.06774	672
	M2	335.07461	672
	M3	336.07796	672
**C5257**	M0	334.09815	237
	M1	335.10151	238
	M2	336.10491	238
	M3	337.10821	237

	M4	338.11157	237
	M5	339.11493	237
	M6	340.11835	237
	M7	341.12174	236
**C5185**	M0	330.54564	2057
	M5	335.56241	2057
**C5266**	M0	334.95648	1890
	M1	335.95994	1890
	M2	336.96324	1890
	M3	337.96661	1890
	M4	338.96997	1890
	M5	339.97302	1890
	M6	340.97601	1890
**C5254**	M0	334.07047	1726
	M1	335.07381	1725
	M2	336.07715	1725
	M3	337.08053	1726
	M4	338.08385	1725
	M5	339.08725	1725
	M6	340.09055	1725
	M7	341.09374	1725
	M8	342.09694	1725
**C5284**	M0	335.09752	260
	M1	336.10090	260
	M2	337.10421	260
	M3	338.10756	260
	M4	339.11104	260
	M5	340.11433	260
	M6	341.11764	260
**C5323**	M0	337.05454	1148
	M1	338.05799	1148
	M2	339.06131	1148
	M3	340.06465	1147
	M4	341.06802	1146
	M5	342.07138	1146
	M6	343.07468	1145
	M7	344.07796	1145
	M8	345.08127	1145
**C5318**	M0	337.01607	2171
	M1	338.01959	2170
	M2	339.02280	2170
	M3	340.02612	2170
	M4	341.02946	2170
	M5	342.03279	2170
	M6	343.03612	2170
**C5320**	M0	337.03380	1848

	M1	338.03719	1847
	M2	339.04064	1847
	M3	340.04398	1847
	M4	341.04730	1847
	M5	342.05057	1847
**C5324**	M0	337.07014	1110
	M1	338.07374	1109
	M2	339.07697	1109
	M3	340.08030	1109
	M4	341.08363	1108
	M5	342.08695	1108
	M6	343.09031	1107
	M7	344.09365	1108
**C5330**	M0	337.08792	938
	M1	338.09141	937
	M2	339.09471	937
	M3	340.09806	937
	M4	341.10138	937
	M5	342.10474	937
	M6	343.10811	935
	M7	344.11148	935
	M8	345.11485	935
	M9	346.11813	935
	M10	347.12152	935
**C5347**	M0	338.09139	1007
	M1	339.09472	1007
	M2	340.09808	1007
	M3	341.10141	1007
	M6	344.11144	1004
**C5354**	M0	338.98784	2087
	M1	339.99120	2087
	M2	340.99454	2087
	M3	341.99769	2087
	M4	343.00114	2087
	M5	344.00436	2087
	M6	345.00782	2087
**C5355**	M0	338.98783	1980
	M1	339.99121	1981
	M6	345.00803	1980
**C5353**	M0	338.98780	1908
	M1	339.99124	1907
**C5362**	M0	339.04476	2109
	M1	340.04807	2109
	M2	341.05156	2109
	M3	342.05485	2109
	M4	343.05819	2109

	M5	344.06147	2109
	M6	345.06484	2109
	M7	346.06785	2110
	M8	347.07093	2110
**C5339**	M0	338.06541	1501
	M1	339.06886	1501
	M2	340.07207	1501
	M3	341.07545	1501
	M4	342.07877	1501
	M5	343.08207	1501
	M6	344.08546	1498
**C5350**	M0	338.54318	2109
	M1	339.54571	2109
	M2	340.54993	2109
	M3	341.55323	2109
	M4	342.55655	2109
	M5	343.55987	2109
	M6	344.56317	2109
	M7	345.56645	2109
	M8	346.56939	2110
**C5376**	M0	339.09548	341
	M2	341.10226	341
**C5416**	M0	340.99402	900
	M1	341.99754	898
	M3	344.00417	894
**C5418**	M0	341.00190	1741
	M1	342.00540	1741
	M2	343.00866	1741
	M3	344.01197	1741
	M4	345.01547	1741
	M5	346.01864	1741
	M6	347.02186	1741
**C4888**	M0	319.04435	139
	M1	320.04773	143
	M24	343.12376	138
**C5474**	M0	342.91528	214
	M1	343.91865	214
**C5479**	M0	343.01915	515
	M1	344.02218	515
	M2	345.02575	515
	M3	346.02927	515
	M4	347.03265	515
	M5	348.03596	513
**C5378**	M0	339.10112	1474
	M3	342.11136	1474
	M4	343.11471	1473

	M5	344.11807	1473
**C5439**	M0	341.96259	698
	M3	344.97273	697
**C5497**	M0	343.99322	2095
	M2	345.99987	2096
	M3	347.00310	2095
	M4	348.00678	2096
**C5414**	M0	340.99207	2087
	M1	341.99601	2086
	M5	346.00879	2087
	M6	347.01242	2087
**C5554**	M0	346.05367	1769
	M1	347.05707	1768
	M2	348.06044	1769
	M3	349.06367	1768
	M4	350.06710	1768
	M6	352.07379	1767
	M7	353.07719	1767
**C5574**	M0	347.03893	2112
	M1	348.04253	2112
	M2	349.04568	2112
	M3	350.04906	2112
	M4	351.05234	2112
	M5	352.05565	2112
	M6	353.05909	2111
	M7	354.06224	2112
	M8	355.06555	2112
**C5556**	M0	346.05494	1180
	M1	347.05851	1179
	M2	348.06169	1179
	M3	349.06506	1179
	M4	350.06843	1178
	M5	351.07179	1177
	M6	352.07514	1177
	M7	353.07852	1176
	M8	354.08173	1177
	M9	355.08512	1176
**C5557**	M0	346.05490	2051
	M1	347.05836	2052
	M2	348.06162	2052
	M3	349.06510	2052
	M4	350.06845	2051
	M5	351.07173	2052
	M6	352.07507	2051
	M7	353.07835	2051
	M8	354.08173	2051

**C5610**	M0	348.98190	1526
	M1	349.98570	1526
	M2	350.98865	1526
	M3	351.99206	1525
	M4	352.99544	1525
	M5	353.99891	1525
	M6	355.00236	1524
	M7	356.00558	1524
**C5614**	M0	349.01683	958
	M1	350.02021	959
	M2	351.02359	955
	M3	352.02661	956
	M4	353.03025	954
	M5	354.03355	954
	M6	355.03691	954
**C5615**	M0	349.01691	894
	M1	350.02029	892
**C5621**	M0	349.05328	875
	M2	351.05992	872
	M3	352.06324	871
	M4	353.06661	871
	M5	354.06991	871
	M6	355.07332	871
	M7	356.07663	871
**C5652**	M0	350.98792	1773
	M1	351.99136	1773
	M2	352.99467	1773
	M3	353.99805	1773
	M4	355.00143	1773
	M5	356.00474	1773
	M6	357.00801	1773
	M7	358.01146	1772
**C5662**	M0	351.07848	633
	M1	352.08216	633
	M2	353.08522	633
	M3	354.08856	633
	M4	355.09201	633
	M5	356.09537	633
	M6	357.09871	633
	M7	358.10188	633
	M8	359.10532	633
**C5608**	M0	348.55635	1969
	M4	352.56957	1967
	M5	353.57293	1968
	M6	354.57629	1968
**C5467**	M0	342.11149	142

	M11	353.14834	143
**C5696**	M0	353.02883	1964
	M1	354.03232	1964
	M2	355.03556	1965
	M3	356.03890	1965
	M4	357.04225	1964
	M5	358.04555	1964
	M6	359.04890	1964
**C5720**	M0	354.03355	1556
	M1	355.03709	1556
	M2	356.04027	1556
	M3	357.04366	1556
	M4	358.04703	1556
	M5	359.05026	1555
	M6	360.05350	1557
**C5745**	M0	355.05574	1309
	M1	356.05957	1309
	M2	357.06248	1308
	M3	358.06581	1308
	M4	359.06916	1308
	M5	360.07247	1308
	M6	361.07582	1307
	M7	362.07915	1307
**C5753**	M0	356.00493	879
	M1	357.00811	878
**C5768**	M0	356.98900	890
	M1	357.99235	889
	M2	358.99575	888
	M3	359.99824	888
	M4	361.00229	888
	M5	362.00568	888
	M6	363.00911	887
**C5769**	M0	356.98901	958
	M1	357.99237	959
	M2	358.99580	955
	M6	363.00911	956
**C5770**	M0	356.98894	1023
	M1	357.99230	1022
	M2	358.99559	1022
	M6	363.00902	1020
**C5802**	M0	358.98362	1742
	M1	359.98693	1741
**C5791**	M0	358.00910	2146
	M2	360.01581	2146
	M3	361.01916	2146
	M4	362.02250	2146

	M5	363.02588	2146
	M6	364.02926	2146
	M7	365.03266	2146
**C5820**	M0	359.98837	2177
	M2	361.99504	2176
	M3	362.99838	2177
	M4	364.00180	2177
	M5	365.00512	2177
	M6	366.00841	2176
**C5828**	M0	360.06943	802
	M3	363.07940	800
	M4	364.08276	800
	M5	365.08615	800
**C5871**	M0	362.96476	1192
	M1	363.96819	1191
**C5856**	M0	362.06561	785
	M1	363.06895	784
	M2	364.07232	782
	M3	365.07571	782
	M4	366.07905	782
	M5	367.08219	782
**C5860**	M0	362.10203	1568
	M2	364.10898	1570
	M3	365.11205	1567
	M4	366.11530	1568
	M5	367.11879	1568
**C5907**	M0	365.04958	1918
	M1	366.05293	1918
	M2	367.05633	1918
	M3	368.05964	1917
	M4	369.06293	1917
	M5	370.06628	1917
	M6	371.06968	1917
	M7	372.07302	1917
**C5855**	M0	362.04996	2217
	M5	367.06684	2212
**C5854**	M0	362.04993	2129
	M5	367.06673	2131
**C5864**	M0	362.60190	2315
	M5	367.61869	2314
	M6	368.62204	2313
	M7	369.62530	2316
	M8	370.62866	2313
	M9	371.63189	2316
**C5928**	M0	366.98867	1890
	M1	367.99203	1889

	M3	369.99836	1889
**C5880**	M0	363.03395	2107
	M5	368.05046	2109
**C5888**	M0	363.10348	2315
	M5	368.12040	2313
	M6	369.12370	2313
	M7	370.12703	2313
	M8	371.13032	2313
**C5890**	M0	363.60975	2288
	M5	368.62647	2289
	M6	369.62990	2286
	M7	370.63327	2286
	M8	371.63661	2286
	M9	372.63991	2286
	M10	373.64321	2286
**C5964**	M0	368.96800	1890
	M1	369.97136	1889
	M2	370.97442	1889
	M3	371.97805	1889
**C5965**	M0	368.99862	2118
	M1	370.00199	2118
	M2	371.00534	2118
	M3	372.00867	2118
	M4	373.01203	2118
	M5	374.01533	2118
	M6	375.01857	2118
	M7	376.02199	2118
**C5954**	M0	368.11907	167
	M2	370.12577	167
	M7	375.14264	167
**C6000**	M0	370.97784	1741
	M1	371.98127	1741
	M2	372.98455	1741
	M3	373.98785	1741
	M4	374.99122	1741
	M5	375.99412	1741
	M6	376.99795	1741
**C6023**	M0	372.03921	2146
	M1	373.04257	2146
	M2	374.04595	2146
	M3	375.04925	2146
	M4	376.05265	2146
	M5	377.05602	2146
	M6	378.05938	2146
	M7	379.06265	2146
**C6015**	M0	371.53756	2146

	M1	372.54024	2146
	M2	373.54436	2146
	M3	374.54770	2146
	M4	375.55103	2146
	M5	376.55435	2146
	M6	377.55767	2146
	M7	378.56104	2146
	M8	379.56424	2146
**C6037**	M0	372.99933	516
	M1	374.00264	516
**C6005**	M0	371.02174	2068
	M3	374.03193	2069
	M5	376.03851	2069
**C6025**	M0	372.06939	1827
	M2	374.07620	1826
	M3	375.07958	1826
	M4	376.08285	1826
	M5	377.08610	1827
**C6065**	M0	374.56359	2058
	M2	376.57042	2058
	M3	377.57362	2058
	M4	378.57700	2058
	M5	379.58041	2057
	M6	380.58365	2057
	M7	381.58700	2057
**C6055**	M0	374.06196	2058
	M3	377.07208	2058
	M4	378.07534	2058
	M5	379.07876	2058
	M6	380.08199	2057
	M7	381.08530	2057
	M8	382.08849	2057
**C6101**	M0	376.94388	2088
	M1	377.94727	2088
	M2	378.95082	2088
	M3	379.95408	2087
	M4	380.95754	2088
	M6	382.96412	2087
**C6108**	M0	377.08489	143
	M1	378.08835	143
	M2	379.09155	149
	M3	380.09477	149
	M4	381.09806	138
	M5	382.10188	141
	M6	383.10509	144
	M7	384.10858	147

	M8	385.11209	147
	M10	387.11870	143
	M11	388.12204	142
	M12	389.12519	142
**C6158**	M0	380.01466	1917
	M1	381.01835	1917
	M2	382.02147	1917
	M3	383.02479	1917
	M4	384.02807	1918
	M5	385.03127	1918
	M6	386.03480	1917
	M7	387.03792	1917
**C6174**	M0	380.93884	1741
	M1	381.94214	1741
	M3	383.94872	1741
**C6198**	M0	382.04589	2275
	M2	384.05245	2271
	M3	385.05587	2275
	M4	386.05931	2274
	M5	387.06237	2269
	M6	388.06536	2267
	M7	389.06861	2267
**C6220**	M0	383.05127	2190
	M1	384.05496	2190
	M2	385.05818	2190
	M3	386.06155	2190
	M4	387.06490	2190
	M5	388.06828	2190
	M6	389.07170	2190
	M7	390.07491	2190
**C6203**	M0	382.10331	289
	M2	384.11049	289
	M0	382.29660	2275
	M2	384.30334	2274
	M3	385.30656	2274
	M4	386.31002	2274
	M5	387.31309	2272
	M6	388.31609	2269
	M7	389.31924	2267
**C6221**	M0	383.09952	290
	M1	384.10303	290
	M2	385.10630	289
	M3	386.10968	290
	M7	390.12323	290
**C6249**	M0	384.80498	2274
	M1	385.80836	2274

	M2	386.81160	2272
	M3	387.81460	2268
	M4	388.81768	2267
	M5	389.82083	2267
**C6252**	M0	384.99374	1729
	M1	385.99714	1729
	M2	387.00037	1730
	M3	388.00357	1729
	M4	389.00731	1730
**C6274**	M0	386.01546	2082
	M1	387.01884	2082
	M2	388.02218	2082
	M3	389.02561	2082
	M4	390.02872	2082
	M5	391.03208	2082
	M6	392.03548	2081
	M7	393.03882	2081
	M8	394.04198	2080
**C6295**	M0	386.97307	959
	M1	387.97643	959
	M3	389.98311	959
**C6296**	M0	386.97871	1743
	M1	387.98210	1743
	M2	388.98510	1742
	M3	389.98873	1742
**C6297**	M0	386.99943	1190
	M1	388.00252	1189
	M2	389.00582	1188
	M4	391.01281	1187
	M5	392.01613	1187
**C6298**	M0	386.99934	999
	M1	388.00280	997
	M2	389.00621	997
	M3	390.00944	997
	M4	391.01282	996
	M5	392.01625	996
**C6275**	M0	386.01527	1733
	M2	388.02190	1733
	M3	389.02534	1733
	M4	390.02880	1735
	M5	391.03212	1735
	M6	392.03525	1735
	M7	393.03850	1734
**C6212**	M0	382.77177	2132
	M6	388.79187	2133
	M7	389.79526	2132

**C6312**	M0	387.08080	1493
	M1	388.08476	1493
	M2	389.08843	1492
	M3	390.09148	1492
	M4	391.09484	1492
	M5	392.09861	1492
	M6	393.10172	1492
	M7	394.10448	1492
**C6209**	M0	382.43738	2132
	M7	389.46084	2132
**C6346**	M0	388.94404	2162
	M1	389.94740	2162
	M2	390.95067	2160
	M3	391.95395	2161
	M4	392.95728	2159
	M5	393.96043	2158
**C6359**	M0	389.03465	634
	M1	390.03822	634
	M2	391.04152	634
	M3	392.04495	634
	M4	393.04843	634
	M5	394.05201	634
	M6	395.05516	634
	M7	396.05853	634
**C6306**	M0	387.04413	557
	M1	388.04747	557
	M3	390.05368	557
	M4	391.05752	557
	M5	392.06116	557
	M6	393.06452	557
	M7	394.06765	556
	M8	395.07158	555
**C6373**	M0	389.08494	802
	M1	390.08832	802
	M2	391.09148	800
	M3	392.09514	800
	M4	393.09856	800
	M5	394.10186	800
	M6	395.10518	800
	M7	396.10845	799
**C6423**	M0	391.06424	272
	M1	392.06743	272
	M0	388.06422	557
	M4	392.07744	553
	M5	393.08085	552
	M6	394.08427	552

	M7	395.08760	553
**C6397**	M0	390.08004	526
	M2	392.08682	527
	M3	393.09023	526
	M4	394.09346	526
	M5	395.09684	527
**C6307**	M0	387.04408	2068
	M6	393.06438	2070
**C6428**	M0	391.10872	177
**C6428**	M1	392.11216	177
	M2	393.11548	177
	M3	394.11893	177
	M4	395.12022	177
	M6	397.12881	177
	M7	398.13232	177
	M8	399.13579	177
	M12	403.14903	177
**C6494**	M0	394.94495	894
	M1	395.94825	892
**C6404**	M0	390.12261	2213
	M8	398.14956	2212
	M9	399.15297	2212
**C6532**	M0	397.10707	2132
	M2	399.11396	2132
	M3	400.11731	2133
	M4	401.12065	2133
	M5	402.12393	2133
	M6	403.12726	2133
	M7	404.13042	2133
	M9	406.13706	2132
**C6535**	M0	397.44127	2132
	M2	399.44840	2133
	M3	400.45173	2133
	M4	401.45510	2133
	M5	402.45840	2133
	M6	403.46167	2133
	M7	404.46485	2132
	M9	406.47144	2133
**C6523**	M0	396.77268	2132
	M1	397.77542	2133
	M3	399.78285	2133
	M4	400.78620	2133
	M5	401.78949	2133
	M6	402.79285	2133
	M7	403.79609	2133
	M8	404.79933	2133

	M9	405.80264	2133
**C6558**	M0	399.00944	2121
	M1	400.01285	2121
**C6542**	M0	398.01567	917
	M1	399.01920	915
	M2	400.02236	915
**C6560**	M0	399.03729	2252
	M2	401.04440	2252
	M3	402.04768	2252
	M4	403.05107	2252
	M5	404.05443	2252
	M6	405.05782	2251
	M7	406.06100	2253
**C6566**	M0	399.09456	280
	M1	400.09812	281
	M2	401.10135	280
	M3	402.10480	281
	M7	406.11803	280
**C6565**	M0	399.09456	178
	M2	401.10135	178
**C6592**	M0	400.88430	1890
	M1	401.88764	1890
**C6593**	M0	401.01509	2146
	M1	402.01977	2146
	M2	403.02216	2146
	M3	404.02557	2146
	M4	405.02897	2146
	M5	406.03233	2146
	M6	407.03574	2146
	M7	408.03904	2146
	M8	409.04231	2147
	M9	410.04523	2147
**C6550**	M0	398.54484	2172
	M4	402.55835	2172
	M5	403.56190	2173
	M6	404.56512	2173
**C6618**	M0	402.01033	2117
	M1	403.01311	2118
	M2	404.01688	2116
	M3	405.02024	2116
	M4	406.02376	2116
	M5	407.02705	2116
	M6	408.03052	2116
	M7	409.03385	2116
	M8	410.03696	2111
	M9	411.03958	2111

**C6617**	M0	402.01037	1818
	M1	403.01393	1818
	M2	404.01727	1818
	M3	405.02060	1818
	M4	406.02388	1818
	M5	407.02723	1818
	M6	408.03059	1817
	M7	409.03367	1816
**C6595**	M0	401.01526	2201
	M1	402.01992	2200
	M2	403.02208	2196
	M3	404.02536	2196
	M4	405.02865	2196
	M5	406.03194	2196
	M6	407.03524	2195
**C6636**	M0	402.99452	1872
	M1	403.99782	1872
	M2	405.00125	1871
	M3	406.00457	1871
**C6634**	M0	402.99448	2208
	M1	403.99805	2208
	M2	405.00128	2208
	M3	406.00457	2208
	M4	407.00797	2207
	M5	408.01128	2208
	M6	409.01466	2208
	M7	410.01754	2203
	M8	411.02095	2205
	M9	412.02337	2203
**C6601**	M0	401.05982	634
	M3	404.06986	633
	M4	405.07326	634
	M5	406.07655	634
	M6	407.07993	634
	M7	408.08328	632
	M8	409.08649	633
**C6638**	M0	403.01159	1945
	M1	404.01500	1946
	M2	405.01845	1946
	M3	406.02184	1946
	M4	407.02520	1946
	M5	408.02855	1946
**C6652**	M0	403.11020	2125
	M1	404.11377	2125
	M2	405.11699	2126
	M3	406.12036	2125

	M4	407.12373	2125
	M5	408.12709	2125
	M6	409.13043	2125
	M7	410.13376	2125
	M8	411.13705	2125
	M9	412.14040	2125
**C6670**	M0	404.05311	1708
	M1	405.05669	1708
	M2	406.05997	1708
	M3	407.06332	1708
	M4	408.06669	1708
	M5	409.07010	1708
	M6	410.07350	1708
	M7	411.07671	1709
**C6677**	M0	404.09599	164
	M1	405.09942	164
	M2	406.10279	164
	M3	407.10636	164
	M6	410.11628	162
	M7	411.11958	164
	M8	412.12375	162
	M12	416.13629	162
**C6741**	M0	406.95511	1979
	M1	407.95845	1979
	M2	408.96156	1979
	M3	409.96476	1979
**C6698**	M0	405.04343	888
	M3	408.05365	886
	M4	409.05697	886
	M5	410.06030	886
	M6	411.06367	886
	M7	412.06696	886
	M8	413.07024	885
**C6723**	M0	406.07521	755
	M1	407.07940	754
	M2	408.08349	752
	M3	409.08685	752
	M4	410.09009	752
	M5	411.09270	752
	M6	412.09551	751
	M7	413.09914	750
	M8	414.10251	750
**C6722**	M0	406.06497	755
	M1	407.06790	754
	M3	409.07475	753
**C6800**	M0	410.02688	2004

	M1	411.03068	2004
	M2	412.03369	2004
	M3	413.03701	2004
	M4	414.04039	2004
	M5	415.04375	2004
	M6	416.04700	2004
	M7	417.05022	2003
	M8	418.05366	2003
**C6776**	M0	408.93406	1741
	M3	411.94414	1741
**C6801**	M0	410.02696	2185
	M1	411.03101	2185
	M2	412.03393	2185
	M3	413.03719	2185
	M4	414.04046	2185
	M5	415.04377	2185
	M6	416.04714	2185
	M7	417.05051	2184
	M8	418.05390	2184
**C6869**	M0	415.10232	443
	M2	417.10920	443
**C6878**	M0	416.98941	1742
	M1	417.99274	1742
**C6881**	M0	417.04518	1076
	M2	419.05182	1076
**C6902**	M0	418.89503	1742
	M1	419.89828	1742
**C6904**	M0	419.02662	1829
	M1	420.03021	1829
	M2	421.03351	1829
	M3	422.03679	1829
	M4	423.04020	1829
	M5	424.04352	1829
	M6	425.04689	1829
	M7	426.05021	1829
	M8	427.05339	1829
**C6885**	M0	417.05492	634
	M2	419.06128	633
	M3	420.06471	635
	M4	421.06819	634
	M5	422.07155	634
	M6	423.07494	634
	M7	424.07818	633
	M8	425.08141	633
	M9	426.08461	633
**C6925**	M0	421.00607	1935

	M1	422.00965	1935
	M2	423.01290	1936
	M3	424.01626	1935
	M4	425.01963	1935
	M5	426.02292	1934
	M6	427.02624	1933
**C6915**	M0	420.06046	530
	M2	422.06703	530
	M3	423.07021	530
	M4	424.07372	530
**C6918**	M0	420.09103	1035
	M2	422.09764	1037
	M7	427.11441	1033
**C6930**	M0	421.07489	674
	M2	423.08167	674
**C6952**	M0	423.02967	2193
	M1	424.03316	2193
**C6931**	M0	421.07501	505
	M3	424.08544	503
	M4	425.08846	504
	M5	426.09175	503
	M6	427.09505	503
	M7	428.09844	503
	M8	429.10176	503
	M9	430.10506	503
**C6942**	M0	422.08724	2001
	M2	424.09389	2002
	M3	425.09714	2002
	M4	426.10052	2001
	M5	427.10386	2002
	M6	428.10719	2002
	M7	429.11054	2002
	M8	430.11398	2001
	M9	431.11739	2001
	M10	432.12091	2001
	M11	433.12454	2001
	M12	434.12819	2001
**C6984**	M0	426.02185	2187
	M1	427.02543	2187
	M2	428.02868	2187
	M3	429.03194	2187
	M4	430.03534	2187
	M5	431.03855	2187
	M6	432.04204	2187
	M7	433.04543	2187
	M8	434.04876	2187

	M9	435.05170	2185
**C7030**	M0	429.06496	183
	M1	430.06842	183
	M2	431.07171	181
	M3	432.07507	181
	M7	436.08857	181
**C7043**	M0	431.03587	519
	M1	432.03926	519
	M2	433.04261	519
**C7063**	M0	433.05168	514
	M1	434.05528	514
	M2	435.05895	513
	M3	436.06209	511
	M4	437.06527	511
	M5	438.06851	511
	M6	439.07180	511
	M7	440.07536	511
	M8	441.07847	511
**C7091**	M0	435.06623	652
	M2	437.07295	652
**C7119**	M0	436.95472	2088
	M1	437.95825	2088
	M2	438.96223	2088
**C7120**	M0	436.96642	1980
	M1	437.96976	1981
	M6	442.98583	1980
**C7131**	M0	437.09927	150
	M1	438.10255	149
**C7092**	M0	435.09056	799
	M3	438.10058	797
	M4	439.10381	797
	M5	440.10732	802
	M6	441.11068	802
	M7	442.11401	802
	M8	443.11744	802
**C7171**	M0	439.08638	139
	M1	440.08993	139
	M2	441.09150	144
	M3	442.09583	147
	M7	446.11010	140
	M11	450.12350	142
	M12	451.12686	142
**C7199**	M0	441.04039	2193
	M1	442.04376	2192
	M11	452.07826	2201
**C7212**	M0	442.01707	2267

	M1	443.02067	2266
	M2	444.02373	2266
	M3	445.02709	2266
	M4	446.03039	2266
	M5	447.03374	2266
	M6	448.03708	2266
	M7	449.04039	2262
	M8	450.04372	2260
	M9	451.04674	2259
	M0	437.08663	2184
	M6	443.10669	2184
	M7	444.11014	2183
	M8	445.11355	2184
	M9	446.11673	2184
	M10	447.12000	2184
	M11	448.12351	2184
**C7157**	M0	438.58384	2157
	M4	442.59710	2157
	M5	443.60061	2157
	M6	444.60398	2157
	M7	445.60727	2156
	M8	446.61061	2156
	M9	447.61392	2157
**C7213**	M0	442.01705	2217
	M1	443.02058	2217
	M2	444.02367	2217
	M3	445.02716	2217
	M4	446.03049	2216
	M5	447.03376	2217
	M6	448.03719	2217
	M7	449.04044	2217
**C7169**	M0	439.08553	2157
	M6	445.10552	2156
	M7	446.10881	2156
	M8	447.11217	2156
**C7204**	M0	441.09150	144
	M1	442.09583	147
	M3	444.10375	152
	M4	445.10703	152
**C7248**	M0	444.03281	1179
	M3	447.04336	1177
**C7307**	M0	448.96562	1773
	M1	449.96889	1773
**C7287**	M0	447.02128	876
	M3	450.03139	870
	M4	451.03472	870

	M5	452.03816	870
	M6	453.04152	870
**C7203**	M0	441.09059	139
	M1	442.09583	147
	M12	453.13084	141
**C7343**	M0	453.06641	2180
	M1	454.07026	2180
	M2	455.07316	2180
	M3	456.07655	2180
	M4	457.07996	2180
	M5	458.08329	2180
	M6	459.08666	2180
**C7358**	M0	455.00555	1773
	M1	456.00890	1773
	M3	458.01542	1773
**C7338**	M0	453.02052	1959
	M3	456.03031	1958
	M4	457.03376	1957
	M5	458.03712	1957
	M6	459.04041	1957
	M7	460.04383	1957
	M8	461.04707	1957
**C7360**	M0	455.09739	2207
	M1	456.10078	2207
	M2	457.10412	2207
	M3	458.10752	2207
	M4	459.11094	2207
	M5	460.11420	2207
	M6	461.11756	2207
	M7	462.12101	2206
	M8	463.12424	2206
	M9	464.12760	2206
	M10	465.13102	2204
	M11	466.13432	2204
	M12	467.13785	2204
**C7388**	M0	458.92712	1982
	M1	459.93009	1982
**C7390**	M0	458.99473	1738
	M1	459.99811	1738
	M2	461.00143	1738
	M4	463.00800	1738
**C7392**	M0	459.03110	1089
	M1	460.03448	1089
	M2	461.03775	1089
	M3	462.04106	1088
	M4	463.04440	1088

	M5	464.04785	1087
	M6	465.05120	1087
	M7	466.05452	1086
	M8	467.05769	1085
**C7389**	M0	458.96204	1936
	M2	460.96825	1936
	M3	461.97162	1936
**C7410**	M0	461.01624	961
	M1	462.01958	962
**C7435**	M0	463.09713	515
	M1	464.10059	515
	M2	465.10352	515
	M3	466.10713	515
	M4	467.11065	515
**C7430**	M0	462.97413	1827
	M3	465.98418	1827
**C7436**	M0	463.10299	1494
	M3	466.11279	1492
	M4	467.11612	1493
**C7469**	M0	465.98250	2080
	M1	466.98591	2080
	M2	467.98922	2080
	M3	468.99259	2080
	M4	469.99594	2080
	M5	470.99920	2080
	M6	472.00258	2080
	M7	473.00584	2080
	M8	474.00881	2079
**C7425**	M0	462.06697	2173
	M5	467.08353	2173
	M6	468.08688	2173
	M7	469.08983	2173
	M8	470.09347	2173
	M9	471.09612	2173
	M10	472.09939	2173
**C7473**	M0	466.09798	167
	M2	468.10478	166
	M7	473.12122	165
**C7502**	M0	468.12952	1828
	M1	469.13295	1828
	M2	470.13625	1828
	M3	471.13971	1828
	M4	472.14314	1828
	M5	473.14654	1828
	M6	474.15005	1828
	M7	475.15329	1828

	M8	476.15659	1827
	M9	477.15984	1827
	M10	478.16308	1827
**C7522**	M0	470.93997	1814
	M1	471.94338	1814
	M2	472.94678	1814
	M3	473.95009	1814
	M4	474.95352	1814
	M5	475.95689	1814
	M6	476.96020	1814
	M7	477.96353	1814
**C7548**	M0	473.01056	1737
	M1	474.01392	1736
	M2	475.01727	1736
	M3	476.02053	1736
	M4	477.02397	1736
	M5	478.02726	1736
	M6	479.03053	1736
**C7525**	M0	471.03114	1089
	M3	474.04115	1088
	M4	475.04471	1087
	M5	476.04802	1087
	M6	477.05136	1086
	M7	478.05462	1086
	M8	479.05788	1085
	M9	480.06096	1085
**C7564**	M0	474.05902	1890
	M2	476.06625	1891
	M3	477.06933	1891
	M4	478.07253	1891
	M5	479.07584	1890
	M6	480.07920	1890
	M7	481.08254	1890
	M8	482.08604	1890
	M9	483.08959	1890
	M10	484.09333	1890
	M11	485.09721	1890
**C7530**	M0	471.09233	2101
	M6	477.11235	2099
	M7	478.11586	2099
	M8	479.11928	2099
	M9	480.12253	2099
	M10	481.12576	2099
**C7570**	M0	474.17319	456
	M1	475.17671	456
	M2	476.17848	456

	M3	477.18321	456
	M4	478.18657	456
	M5	479.18998	456
	M6	480.19331	455
	M7	481.19672	455
	M8	482.20013	455
	M9	483.20331	455
	M10	484.20687	455
	M11	485.21014	455
	M12	486.21354	454
	M13	487.21693	454
**C7628**	M0	479.04587	1935
	M1	480.04913	1934
	M2	481.05261	1935
	M3	482.05608	1935
	M4	483.05941	1935
	M5	484.06269	1934
	M6	485.06606	1934
**C7651**	M0	480.98233	2200
	M1	481.98604	2200
	M2	482.98891	2200
	M3	483.99218	2200
	M4	484.99533	2200
	M5	485.99879	2200
	M6	487.00205	2199
	M7	488.00541	2199
**C7658**	M0	481.97752	2116
	M1	482.98099	2117
	M2	483.98428	2116
	M3	484.98751	2116
	M4	485.99088	2116
	M5	486.99422	2116
	M6	487.99754	2116
	M7	489.00089	2116
	M8	490.00412	2116
	M9	491.00621	2111
**C7669**	M0	482.96167	2209
	M1	483.96510	2210
	M2	484.96837	2210
	M3	485.97165	2209
	M4	486.97497	2209
	M5	487.97834	2209
	M6	488.98169	2209
	M7	489.98492	2209
	M8	490.98792	2210
	M9	491.99024	2204

**C7678**	M0	483.29652	146
	M1	484.30016	146
**C7663**	M0	482.09752	755
	M2	484.10413	753
	M3	485.10756	753
**C7716**	M0	486.92216	1773
	M1	487.92512	1773
**C7722**	M0	487.05549	518
	M1	488.05904	518
	M3	490.06528	518
**C7723**	M0	487.06273	480
	M1	488.06614	480
	M2	489.06952	480
**C7754**	M0	489.99400	2185
	M1	490.99761	2185
	M2	492.00070	2185
	M3	493.00408	2185
	M4	494.00745	2185
	M5	495.01082	2185
	M6	496.01418	2185
	M7	497.01735	2185
	M8	498.02074	2184
**C7752**	M0	489.11882	1494
	M3	492.12862	1493
	M4	493.13202	1493
**C7771**	M0	491.09818	1494
	M1	492.10147	1494
	M2	493.10480	1494
	M3	494.10828	1493
	M4	495.11161	1493
	M5	496.11484	1493
	M6	497.11810	1493
**C7787**	M0	492.91335	884
	M1	493.91661	881
	M6	498.93344	881
**C7779**	M0	492.06983	1741
	M1	493.07323	1741
	M2	494.07609	1741
	M3	495.07964	1741
	M4	496.08308	1741
	M5	497.08659	1741
	M6	498.08995	1741
	M7	499.09337	1741
	M8	500.09676	1742
	M9	501.10048	1742
	M10	502.10429	1742

**C7801**	M0	493.10789	1022
	M1	494.11134	1019
	M0	494.04753	1863
	M1	495.05083	1863
	M2	496.05410	1863
	M3	497.05737	1862
	M4	498.06078	1862
	M5	499.06404	1862
	M6	500.06731	1862
	M7	501.07076	1862
	M8	502.07402	1862
	M9	503.07740	1861
	M10	504.08039	1861
**C7828**	M0	495.12198	675
	M1	496.12542	675
	M2	497.12860	675
	M3	498.13218	675
	M4	499.13554	675
	M5	500.13877	675
	M6	501.14218	674
	M7	502.14533	674
	M8	503.14866	675
**C7831**	M0	495.18368	225
	M1	496.18718	225
	M2	497.19043	225
	M3	498.19385	224
	M4	499.19723	224
	M5	500.20061	224
	M6	501.20391	224
	M7	502.20723	224
	M8	503.21063	224
	M9	504.21400	224
	M10	505.21729	224
	M11	506.22063	225
	M12	507.22397	226
**C7847**	M0	497.02761	1934
	M1	498.03105	1934
	M2	499.03453	1934
	M3	500.03775	1934
	M4	501.04112	1933
**C7913**	M0	502.03084	1708
	M2	504.03734	1709
	M3	505.04085	1710
	M4	506.04400	1700
**C7945**	M0	504.08787	557
	M1	505.09085	558

	M2	506.09407	557
**C7962**	M0	505.98900	2187
	M1	506.99234	2187
	M2	507.99573	2187
	M3	508.99900	2187
	M4	510.00234	2187
	M5	511.00564	2187
	M6	512.00906	2187
	M7	513.01225	2187
	M8	514.01563	2187
	M9	515.01883	2187
	M10	516.02078	2185
	M35	541.10485	2184
	M36	542.10831	2184
	M37	543.11176	2184
	M38	544.11487	2184
	M39	545.11791	2184
**C7953**	M0	505.05944	1944
	M2	507.06653	1945
	M3	508.06984	1944
	M4	509.07314	1945
**C7980**	M0	507.03155	1773
	M1	508.03495	1773
	M2	509.03833	1773
	M3	510.04169	1773
	M4	511.04509	1773
	M5	512.04844	1773
	M6	513.05167	1773
	M7	514.05505	1773
**C7943**	M0	504.06816	1790
	M5	509.08488	1789
	M6	510.08825	1789
	M7	511.09164	1789
	M8	512.09521	1789
	M9	513.09879	1789
	M10	514.10196	1789
**C8012**	M0	508.11335	756
	M2	510.12014	752
	M3	511.12340	752
**C8046**	M0	510.39918	2276
	M1	511.40361	2275
	M2	512.40696	2275
	M3	513.41035	2275
	M4	514.41362	2274
	M5	515.41705	2274
	M6	516.42020	2275

	M7	517.42334	2269
	M8	518.42639	2268
	M9	519.42939	2267
**C8031**	M0	509.73164	2275
	M1	510.73333	2275
	M2	511.73817	2275
	M3	512.74151	2275
	M4	513.74478	2274
	M5	514.74810	2275
	M6	515.75142	2275
	M7	516.75461	2274
	M8	517.75770	2269
	M9	518.76076	2267
	M10	519.76392	2267
**C8038**	M0	510.06591	2275
	M2	512.07245	2275
	M3	513.07579	2275
	M4	514.07916	2274
	M5	515.08259	2274
	M6	516.08587	2274
	M7	517.08898	2269
	M8	518.09206	2269
	M9	519.09508	2267
	M10	520.09879	2267
**C8082**	M0	513.07695	1824
	M1	514.08042	1824
	M2	515.08359	1824
	M3	516.08714	1824
	M4	517.09044	1824
	M5	518.09377	1824
	M6	519.09699	1824
	M7	520.10042	1824
	M8	521.10372	1824
	M9	522.10688	1823
	M10	523.11024	1820
	M11	524.11348	1820
	M12	525.11668	1820
**C8087**	M0	513.13272	650
	M1	514.13568	650
	M3	516.14268	650
	M4	517.14595	650
	M5	518.14935	650
**C8135**	M0	518.06877	895
	M2	520.07526	894
	M4	522.08215	892
	M5	523.08542	892

	M6	524.08868	891
	M7	525.09167	890
**C8143**	M0	519.05282	892
	M1	520.05608	892
	M2	521.05954	890
	M3	522.06292	890
	M4	523.06572	890
	M5	524.06952	890
	M6	525.07281	890
	M7	526.07630	889
	M8	527.07961	889
	M9	528.08276	888
	M10	529.08589	888
	M12	531.09295	889
**C8162**	M0	520.91785	2211
	M1	521.92113	2210
	M2	522.92455	2211
	M3	523.92773	2211
	M4	524.93075	2210
	M5	525.93402	2203
	M6	526.93737	2204
**C8152**	M0	519.93373	2117
	M2	521.94035	2117
	M3	522.94345	2116
	M4	523.94695	2111
	M5	524.95037	2111
**C8145**	M0	519.05279	1023
	M2	521.05965	1022
	M3	522.06282	1023
	M4	523.06625	1023
	M5	524.06955	1022
	M6	525.07285	1022
	M7	526.07615	1021
	M8	527.07942	1021
	M9	528.08270	1021
**C8181**	M0	521.98406	2268
	M1	522.98751	2268
	M2	523.99077	2269
	M3	524.99404	2268
	M4	525.99741	2268
	M5	527.00070	2268
	M6	528.00402	2268
	M7	529.00736	2268
	M8	530.01061	2269
	M9	531.01358	2267
**C8170**	M0	521.06373	1827

	M1	522.06717	1828
	M2	523.07078	1829
	M3	524.07414	1829
	M4	525.07739	1828
	M5	526.08057	1827
**C8173**	M0	521.10306	540
	M1	522.10631	539
	M2	523.10939	539
	M3	524.11274	539
	M4	525.11621	539
**C8213**	M0	524.00103	2050
	M1	525.00341	2051
	M2	526.00753	2052
	M3	527.01081	2052
	M4	528.01407	2051
	M5	529.01717	2051
	M6	530.01960	2051
**C8156**	M0	520.07444	705
	M5	525.09153	706
	M6	526.09484	706
	M7	527.09812	705
	M8	528.10150	706
	M9	529.10482	705
**C8194**	M0	523.04362	1934
	M2	525.05032	1935
	M3	526.05380	1934
**C8183**	M0	522.04252	1959
	M4	526.05573	1957
	M6	528.06253	1957
	M7	529.06590	1957
	M8	530.06920	1957
	M9	531.07247	1957
	M10	532.07575	1956
**C8235**	M0	525.02282	1935
	M1	526.02617	1935
	M2	527.02970	1935
	M3	528.03308	1934
	M4	529.03646	1934
	M5	530.03985	1934
	M6	531.04325	1933
**C8208**	M0	523.16885	2163
	M1	524.17227	2163
	M4	527.18223	2163
	M5	528.18566	2163
	M6	529.18900	2163
	M7	530.19240	2163

	M8	531.19572	2163
	M9	532.19908	2163
	M10	533.20248	2163
	M11	534.20564	2163
	M12	535.20899	2163
	M13	536.21256	2163
	M14	537.21589	2163
	M15	538.21921	2163
	M16	539.22258	2163
**C8227**	M0	524.62703	2174
	M1	525.62977	2174
	M5	529.64370	2174
	M6	530.64705	2174
	M7	531.65045	2174
	M8	532.65378	2175
	M9	533.65722	2175
	M10	534.66040	2175
	M11	535.66387	2175
**C8249**	M0	525.12864	2174
	M5	530.14548	2174
	M6	531.14873	2174
	M7	532.15213	2174
	M8	533.15553	2175
	M9	534.15886	2175
	M10	535.16220	2174
**C8311**	M0	529.18927	155
	M6	535.20925	157
	M7	536.21266	157
	M8	537.21599	157
	M9	538.21929	157
	M10	539.22264	157
	M11	540.22598	157
	M12	541.22927	157
	M13	542.23268	156
**C8345**	M0	533.07826	676
	M1	534.08138	677
	M3	536.08807	676
	M4	537.09134	676
**C8363**	M0	535.11905	482
	M1	536.12255	481
	M2	537.12615	481
	M3	538.12865	481
	M4	539.13224	481
	M5	540.13603	481
	M6	541.13932	481
	M7	542.14274	481

**C8334**	M0	531.12413	1494
	M6	537.14390	1503
	M7	538.14737	1503
	M8	539.15071	1504
	M9	540.15404	1504
	M10	541.15733	1504
	M11	542.16053	1504
**C8412**	M0	539.08334	938
	M1	540.08654	938
	M2	541.08976	937
	M3	542.09330	937
	M4	543.09664	938
**C8436**	M0	540.98309	2087
	M1	541.98631	2087
**C8475**	M0	543.94535	2187
	M1	544.94825	2187
	M2	545.95186	2187
	M3	546.95524	2187
	M4	547.95856	2187
	M5	548.96162	2187
	M6	549.96514	2185
**C8465**	M0	542.95656	1526
	M1	543.95928	1526
	M3	545.96616	1526
	M4	546.96957	1526
	M5	547.97280	1525
	M6	548.97602	1525
**C8521**	M0	551.11406	525
	M1	552.11740	525
	M2	553.12037	525
**C8517**	M0	550.11852	755
	M1	551.12146	755
	M3	553.12850	752
**C8536**	M0	554.12898	511
	M1	555.13236	511
	M2	556.13517	511
	M3	557.13873	503
	M4	558.14172	510
	M5	559.14567	511
**C8554**	M0	557.00455	894
	M2	559.01119	892
**C8584**	M0	561.95209	2051
	M1	562.95425	2052
	M2	563.95805	2052
	M3	564.96147	2052
	M4	565.96422	2051

**C8605**	M0	564.17470	172
	M1	565.17813	172
	M2	566.18108	170
	M3	567.18472	172
	M4	568.18816	172
	M5	569.18969	171
	M7	571.19824	171
	M8	572.20148	172
	M9	573.20488	172
	M10	574.20601	171
	M12	576.21464	170
	M13	577.21823	172
**C8612**	M0	565.04429	1959
	M1	566.04778	1959
	M2	567.05123	1959
	M3	568.05448	1959
	M4	569.05779	1959
	M5	570.06110	1959
	M6	571.06443	1959
	M7	572.06777	1959
	M8	573.07113	1958
	M9	574.07442	1958
	M10	575.07772	1958
	M11	576.08111	1958
	M12	577.08435	1958
	M13	578.08710	1958
**C8591**	M0	563.06491	1863
	M1	564.06786	1862
	M4	567.07772	1862
	M5	568.08116	1862
	M6	569.08467	1862
	M7	570.08769	1862
	M8	571.09151	1862
	M9	572.09484	1862
	M10	573.09812	1862
	M11	574.10135	1862
	M12	575.10457	1861
**C8688**	M0	573.07080	481
	M1	574.07409	480
	M2	575.07730	480
**C8708**	M0	577.08366	517
	M1	578.08715	516
	M2	579.09050	516
	M6	583.10366	516
**C8728**	M0	579.95118	2117
	M1	580.95372	2117

	M2	581.95713	2117
	M3	582.96025	2116
	M4	583.96349	2116
**C8718**	M0	579.02369	2186
	M1	580.02656	2185
	M3	582.03363	2186
	M4	583.03668	2186
	M5	584.04010	2186
	M6	585.04348	2185
	M7	586.04697	2185
	M8	587.05013	2185
	M9	588.05347	2185
	M10	589.05682	2185
**C8733**	M0	580.80531	1478
	M9	589.83520	1482
	M10	590.83865	1482
**C8758**	M0	586.00997	2090
	M6	592.02991	2092
	M7	593.03310	2092
	M8	594.03635	2092
**C8780**	M0	591.08111	509
	M1	592.08479	509
	M0	592.13623	451
	M1	593.13966	449
	M2	594.14299	449
	M3	595.14623	449
**C8769**	M0	588.07159	1031
	M3	591.08124	1030
	M4	592.08454	1029
	M7	595.09503	1031
	M8	596.09818	1031
	M9	597.10148	1031
**C8811**	M0	596.16141	2132
	M1	597.16398	2132
	M2	598.16840	2133
	M3	599.17183	2133
	M4	600.17510	2133
	M5	601.17844	2133
	M6	602.18169	2133
	M7	603.18503	2133
	M8	604.18835	2133
	M9	605.19166	2133
	M10	606.19494	2133
	M11	607.19815	2133
	M12	608.20146	2132
	M13	609.20452	2132

	M14	610.20780	2133
	M15	611.21101	2133
**C8806**	M0	595.66002	2132
	M1	596.66268	2132
	M2	597.66526	2132
	M3	598.67012	2133
	M4	599.67348	2133
	M5	600.67676	2133
	M6	601.68011	2133
	M7	602.68343	2133
	M8	603.68671	2133
	M9	604.69002	2133
	M10	605.69332	2133
	M11	606.69660	2133
	M12	607.69990	2133
	M13	608.70296	2132
	M15	610.70937	2132
**C8798**	M0	594.09764	1731
	M8	602.12435	1729
	M10	604.13104	1729
**C8880**	M0	606.07089	1863
	M1	607.07425	1863
	M2	608.07774	1863
	M3	609.08103	1863
	M4	610.08435	1863
	M5	611.08763	1863
	M6	612.09099	1863
	M7	613.09429	1863
	M8	614.09761	1863
	M9	615.10094	1863
	M10	616.10420	1862
	M11	617.10756	1862
	M12	618.11087	1862
	M13	619.11418	1862
	M14	620.11721	1862
	M15	621.11990	1861
**C8872**	M0	605.13144	550
	M1	606.13448	549
	M3	608.14117	548
**C8876**	M0	605.67301	2067
	M9	614.70301	2068
	M10	615.70640	2068
	M11	616.70981	2069
	M12	617.71295	2068
**C8844**	M0	601.13558	144
	M1	602.13879	144

	M18	619.19538	141
**C8989**	M0	620.01645	2098
	M5	625.03321	2097
	M6	626.03665	2098
	M7	627.03998	2097
	M8	628.04333	2098
	M9	629.04665	2098
	M10	630.04985	2098
**C8990**	M0	620.05013	2169
	M6	626.07027	2169
	M7	627.07349	2169
	M8	628.07698	2169
	M9	629.08025	2169
	M10	630.08350	2169
	M11	631.08671	2168
**C9024**	M0	628.12975	1829
	M1	629.13265	1829
	M4	632.14261	1830
	M5	633.14625	1830
	M6	634.14952	1829
	M7	635.15294	1829
	M8	636.15635	1829
	M9	637.15970	1829
	M10	638.16309	1829
	M11	639.16642	1829
	M12	640.16982	1829
	M13	641.17314	1829
	M14	642.17633	1829
	M15	643.17941	1828
**C9092**	M0	638.02750	2181
	M1	639.03034	2181
	M2	640.03384	2182
	M3	641.03720	2182
	M4	642.04046	2182
	M5	643.04385	2181
	M6	644.04712	2181
	M7	645.05067	2181
	M8	646.05383	2181
	M9	647.05729	2181
	M10	648.06050	2181
**C9076**	M0	636.09251	1186
	M5	641.10921	1184
	M6	642.11269	1183
	M7	643.11601	1183
	M8	644.11931	1182
	M9	645.12262	1182

	M10	646.12597	1182
	M11	647.12920	1181
	M12	648.13237	1182
**C9144**	M0	645.14984	2133
	M5	650.16642	2133
	M6	651.16989	2133
	M7	652.17314	2133
	M8	653.17634	2132
**C9137**	M0	644.64829	2133
	M1	645.65075	2132
	M5	649.66480	2133
	M6	650.66821	2133
	M7	651.67150	2133
	M8	652.67477	2133
	M9	653.67817	2133
**C9193**	M0	654.97181	893
	M2	656.97852	891
	M7	661.99496	890
**C9227**	M0	663.07575	184
	M3	666.08561	182
**C9236**	M0	670.67111	259
	M10	680.70382	253
	M12	682.71001	253
	M13	683.71291	249
**C9277**	M0	683.92841	2188
	M2	685.93488	2187
	M3	686.93822	2188
	M4	687.94154	2188
	M5	688.94463	2187
**C9384**	M0	728.22452	2288
	M13	741.26797	2286
	M14	742.27154	2286
	M15	743.27477	2286
	M16	744.27808	2286
	M17	745.28157	2286
	M18	746.28477	2286
	M19	747.28804	2286
	M20	748.29143	2286
**C9391**	M0	733.20743	261
	M1	734.21027	261
	M10	743.24080	256
	M11	744.24420	254
	M12	745.24768	254
	M13	746.25118	252
**C9398**	M0	735.69836	232
	M1	736.69873	232

	M5	740.71277	231
	M7	742.72006	229
	M8	743.72306	229
	M9	744.72668	230
	M10	745.72989	230
**C9393**	M0	733.70915	260
	M8	741.73586	263
	M9	742.73933	262
	M10	743.74259	255
	M11	744.74595	253
	M12	745.74942	253
**C9429**	M0	744.08037	2146
	M1	745.08313	2146
	M4	748.09343	2146
	M5	749.09641	2146
	M6	750.10022	2146
	M7	751.10331	2146
	M8	752.10675	2146
	M9	753.11010	2146
	M10	754.11357	2146
	M11	755.11698	2146
	M12	756.12024	2146
	M13	757.12348	2146
**C9453**	M0	749.12892	2058
	M1	750.13204	2058
	M8	757.15528	2057
	M9	758.15872	2057
	M10	759.16231	2057
	M11	760.16556	2057
	M12	761.16881	2057
	M13	762.17221	2057
	M14	763.17566	2057
	M15	764.17884	2057
**C9499**	M0	765.09651	2276
	M6	771.11578	2269
	M7	772.11955	2270
	M8	773.12299	2269
	M9	774.12622	2269
	M10	775.12938	2269
	M11	776.13267	2268
	M12	777.13586	2268
	M13	778.13897	2268
	M14	779.14187	2267
	M15	780.14502	2267
**C9501**	M0	765.59778	2276
	M5	770.61438	2270

	M6	771.61776	2272
	M7	772.62104	2270
	M8	773.62455	2269
	M9	774.62766	2269
	M10	775.63092	2268
	M11	776.63419	2267
	M12	777.63724	2268
	M13	778.64028	2268
	M14	779.64343	2267
**C9565**	M0	779.07775	893
	M8	787.10350	889
	M9	788.10706	889
	M10	789.11065	889
	M11	790.11398	889
**C9592**	M0	794.71144	613
	M1	795.71432	613
	M5	799.72809	612
	M6	800.73136	611
	M7	801.73463	612
	M8	802.73822	611
	M9	803.74139	611
	M10	804.74482	611
	M11	805.74803	611
	M12	806.75130	611
	M13	807.75474	610
	M14	808.75809	608
	M15	809.76141	607
	M16	810.76455	607
**C9595**	M0	795.21306	612
	M1	796.21578	612
	M4	799.22627	611
	M5	800.22952	612
	M6	801.23304	612
	M7	802.23649	612
	M8	803.23966	611
	M9	804.24302	612
	M10	805.24650	611
	M11	806.24969	610
	M12	807.25307	611
	M13	808.25621	609
	M14	809.25977	607
	M15	810.26295	607
**C9585**	M0	790.68669	1377
	M11	801.72337	1380
	M12	802.72671	1380
	M13	803.73005	1380

	M14	804.73328	1378
**C9588**	M0	791.18827	1378
	M10	801.22169	1380
	M11	802.22505	1379
	M12	803.22847	1380
	M13	804.23153	1379
**C9623**	M0	803.10026	519
	M1	804.10352	519
	M3	806.10994	519
**C9601**	M0	798.07635	2252
	M1	799.07896	2252
	M7	805.09916	2252
	M8	806.10281	2252
	M9	807.10608	2251
	M10	808.10950	2252
	M11	809.11276	2252
	M12	810.11599	2252
	M13	811.11908	2252
**C9657**	M0	810.71034	696
	M1	811.71283	696
	M4	814.72336	694
	M5	815.72710	694
	M6	816.73033	694
	M7	817.73365	694
	M8	818.73719	694
	M9	819.74063	694
	M10	820.74389	694
	M11	821.74720	693
	M12	822.75048	693
	M13	823.75367	693
	M14	824.75701	694
	M15	825.76035	694
	M16	826.76367	694
	M17	827.76691	694
	M18	828.77028	694
**C9655**	M0	810.20868	696
	M1	811.21150	696
	M2	812.21394	696
	M5	815.22516	694
	M6	816.22857	694
	M7	817.23210	694
	M8	818.23551	694
	M9	819.23876	694
	M10	820.24212	694
	M11	821.24555	693
	M12	822.24879	693

	M13	823.25211	694
	M14	824.25545	694
	M15	825.25870	694
	M16	826.26205	694
	M17	827.26535	694
	M18	828.26863	694
**C9646**	M0	808.61884	1217
	M1	809.62171	1217
	M13	821.66236	1213
	M14	822.66569	1213
	M15	823.66896	1212
	M16	824.67221	1212
	M17	825.67536	1211
	M18	826.67870	1211
	M19	827.68198	1213
**C9663**	M0	814.23363	241
	M1	815.23661	241
	M11	825.27044	243
	M12	826.27386	243
	M13	827.27726	243
	M14	828.28059	243
	M15	829.28403	244
	M17	831.29197	243
**C9665**	M0	814.73531	241
	M10	824.76849	243
	M11	825.77212	243
	M12	826.77549	244
	M13	827.77895	244
	M14	828.78225	244
	M15	829.78567	244
	M17	831.79338	244
**C9743**	M0	832.72358	245
	M1	833.72359	245
	M5	837.73767	239
	M7	839.74607	246
	M8	840.74896	246
	M9	841.75239	245
	M10	842.75563	246
	M11	843.75883	244
	M12	844.76222	245
	M13	845.76607	250
	M14	846.76929	249
	M15	847.77232	249
	M16	848.77521	247
	M17	849.77827	245
	M18	850.78145	243

	M19	851.78488	243
	M20	852.78837	242
	M21	853.79179	242
**C9786**	M0	853.04761	2051
	M1	854.05039	2051
	M5	858.06414	2052
	M6	859.06749	2051
	M7	860.07066	2051
	M8	861.07408	2051
	M9	862.07767	2051
	M10	863.08083	2050
	M11	864.08413	2051
	M12	865.08735	2050
	M13	866.09053	2050
	M14	867.09370	2050
**C9768**	M0	847.17434	700
	M11	858.21078	696
	M12	859.21424	696
	M13	860.21723	695
	M14	861.22035	695
	M15	862.22343	695
	M16	863.22625	695
**C9794**	M0	856.21552	1293
	M1	857.21852	1293
	M7	863.23878	1289
	M8	864.24223	1289
	M9	865.24552	1289
	M10	866.24894	1288
	M11	867.25227	1291
	M12	868.25558	1294
	M13	869.25896	1293
	M14	870.26233	1293
	M15	871.26562	1292
	M16	872.26896	1293
**C9795**	M0	856.71724	1293
	M7	863.74053	1289
	M8	864.74394	1289
	M9	865.74728	1289
	M10	866.75040	1288
	M11	867.75397	1294
	M12	868.75730	1294
	M13	869.76061	1293
	M14	870.76386	1292
	M15	871.76732	1293
	M16	872.77042	1292
**C9840**	M0	871.71278	1477

	M1	872.71572	1477
	M2	873.71834	1477
	M5	876.72942	1476
	M6	877.73280	1475
	M7	878.73630	1475
	M8	879.73956	1475
	M9	880.74290	1475
	M10	881.74625	1475
	M11	882.74953	1475
	M12	883.75293	1475
	M13	884.75624	1474
	M14	885.75949	1478
	M15	886.76280	1481
	M16	887.76608	1481
	M17	888.76938	1481
	M18	889.77264	1481
**C9843**	M0	872.21446	1477
	M1	873.21716	1477
	M4	876.22778	1475
	M5	877.23122	1475
	M6	878.23456	1475
	M7	879.23790	1475
	M8	880.24116	1475
	M9	881.24457	1475
	M10	882.24795	1475
	M11	883.25124	1474
	M12	884.25455	1474
	M13	885.25791	1474
	M14	886.26107	1474
	M15	887.26447	1481
	M16	888.26773	1481
	M17	889.27094	1480
**C9929**	M0	891.11605	522
	M1	892.11926	522
**C9944**	M0	893.13083	518
	M1	894.13368	518
**C9936**	M0	891.73647	687
	M1	892.73886	688
	M6	897.75606	686
	M8	899.76301	685
	M9	900.76646	685
	M10	901.76988	686
	M11	902.77330	686
	M12	903.77648	685
	M13	904.77971	685
	M14	905.78323	686

	M15	906.78658	685
	M16	907.78978	685
	M17	908.79312	685
	M18	909.79638	685
	M19	910.79967	685
**C9905**	M0	887.20991	1495
	M1	888.21259	1495
	M12	899.25013	1504
	M13	900.25346	1504
	M14	901.25693	1503
	M15	902.26051	1503
	M16	903.26446	1503
**C9932**	M0	891.23470	687
	M1	892.23771	687
	M2	893.24011	688
	M8	899.26144	685
	M9	900.26482	685
	M10	901.26829	685
	M11	902.27168	686
	M12	903.27504	686
	M13	904.27827	686
	M14	905.28157	686
	M15	906.28488	685
	M16	907.28817	685
	M17	908.29153	685
	M18	909.29474	685
	M19	910.29807	684
	M20	911.30137	685
**C9949**	M0	895.25974	242
	M13	908.30293	244
	M14	909.30638	243
	M15	910.30982	243
	M16	911.31331	243
	M17	912.31693	243
	M18	913.32001	243
**C10004**	M0	913.24786	245
	M1	914.24888	245
	M5	918.26185	237
	M14	927.29403	247
	M15	928.29722	248
	M16	929.30039	247
	M17	930.30344	246
	M18	931.30652	245
	M19	932.30973	244
	M20	933.31348	242
**C10098**	M0	971.09617	1935

	M1	972.09930	1935
	M7	978.11840	1936
	M8	979.12282	1935
	M9	980.12564	1935
	M10	981.12879	1935
	M11	982.13169	1934
	M12	983.13485	1934
	M13	984.13889	1934
	M14	985.14202	1933
	M15	986.14493	1933
**C10087**	M0	968.23623	1480
	M1	969.23889	1480
	M13	981.27989	1484
	M14	982.28299	1484
	M15	983.28631	1484
	M16	984.28981	1484
	M18	986.29636	1484
